# JAZ Repressors: Potential Involvement in Nutrients Deficiency Response in Rice and Chickpea

**DOI:** 10.3389/fpls.2015.00975

**Published:** 2015-11-10

**Authors:** Ajit P. Singh, Bipin K. Pandey, Priyanka Deveshwar, Laxmi Narnoliya, Swarup K. Parida, Jitender Giri

**Affiliations:** ^1^National Institute of Plant Genome Research, Jawaharlal Nehru UniversityNew Delhi, India; ^2^Department of Botany, Sri Aurobindo College, University of DelhiNew Delhi, India

**Keywords:** jasmonates, nutrient deficiency, root, gene expression, jas degron, TIFY

## Abstract

Jasmonates (JA) are well-known phytohormones which play important roles in plant development and defense against pathogens. Jasmonate ZIM domain (JAZ) proteins are plant-specific proteins and act as transcriptional repressors of JA-responsive genes. JA regulates both biotic and abiotic stress responses in plants; however, its role in nutrient deficiency responses is very elusive. Although, JA is well-known for root growth inhibition, little is known about behavior of *JAZ* genes in response to nutrient deficiencies, under which root architectural alteration is an important adaptation. Using protein sequence homology and a conserved-domains approach, here we identify 10 novel *JAZ* genes from the recently sequenced Chickpea genome, which is one of the most nutrient efficient crops. Both rice and chickpea *JAZ* genes express in tissue- and stimuli-specific manners. Many of which are preferentially expressed in root. Our analysis further showed differential expression of *JAZ* genes under macro (NPK) and micronutrients (Zn, Fe) deficiency in rice and chickpea roots. While both rice and chickpea *JAZ* genes showed a certain level of specificity toward type of nutrient deficiency, generally majority of them showed induction under K deficiency. Generally, *JAZ* genes showed an induction at early stages of stress and expression declined at later stages of macro-nutrient deficiency. Our results suggest that *JAZ* genes might play a role in early nutrient deficiency response both in monocot and dicot roots, and information generated here can be further used for understanding the possible roles of JA in root architectural alterations for nutrient deficiency adaptations.

## Introduction

Jasmonates (JAs) form a family of oxylipin phytohormones, derived from oxidation of 18 and 16 carbon tri-unsaturated fatty acids (Wasternack and Kombrink, 2010). These phytohormones are known to regulate a wide-range of processes including spikelet development (Cai et al., 2014), senescence (He et al., 2002), root growth (Staswick et al., 1992), communication (both interplant and intra-plant for defense) (Okada et al., 2014) and defense responses against biotic stress (Feys et al., 1994) through degradation of JA signaling repressor proteins (JAZs) (Kazan and Manners, 2012). JA-Isoleucine (JA-Ile), a bioactive form of JA, binds to its receptor complex consisting of CORONATINE-INSENSITIVE1 (COI1), an F-box E3-ubiquitin ligase protein and JAZ repressor (Yan et al., 2009). This COI-JA-Ile complex interacts with JAZ proteins which contain at least two conserved regions, namely, TIFY and Jas at N and C terminal, respectively. The TIFY motif of the JAZ proteins mediates homo- and heteromeric interactions (Chini et al., 2009; Pauwels and Goossens, 2011), whereas the Jas motif is necessary for interaction of JAZ proteins with COI1 in the presence of JA-Ile (Xie et al., 1998), leading to the degradation of JAZ repressors through 26S proteasomal pathway. The Jas motif also mediates interaction with MYC2 (a bHLH transcription factor regulating JA responsive genes) and facilitates inhibition of MYC2 activity (Chini et al., 2007). Therefore, in the absence of JA-Ile, a JAZ protein remains bound to MYC2, and inhibits the transcription of JA responsive genes. Interaction of JAZ with COI^SCF^ complex leads to the degradation of JAZ proteins resulting in release of MYC transcription factors and thus allowing JA responsive genes to be transcribed (Chini et al., 2007; Thines et al., 2007). A few JAZ proteins in Arabidopsis also have an EAR (Ethylene-responsive element binding factor-associated Amphiphilic Repression) motif which allows direct binding of JAZ proteins to TOPLESS LIKE (TPL) without involvement of the Novel Interactor of JAZ (NINJA), an adapter protein. TIFY domains of a few JAZ proteins are also involved in the interaction with NINJA, which further recruits TPL through its EAR motif (Pauwels et al., 2010). Moreover, TPL recruits Histone Deacetylases (HDA6 & HDA19) which further suppress the gene expression via chromatin remodeling (Zhou et al., 2005; Wu et al., 2008).

Overexpression of *JAZ* without Jas motif (*JAZ1*-Δ*Jas*) resulted in male-sterile plants (Thines et al., 2007). Similarly, overexpression of the truncated splice variant of *AtJAZ10* (AtJAZ10.4) which was resistant to COI^SCF^-mediated degradation, produced male-sterile plants (Chung and Howe, 2009). This observation was again validated by Cai et al. (2014) showing that OsJAZ1 could regulate the expression of E-class genes such as *OsMADS1, OsMADS7*, and *OsMADS8* which have roles in inflorescence and spikelet development, resulting in defected spikelet development in rice. It was further shown that Arabidopsis overexpressing *JAZ1*-Δ*Jas* has reduced host resistance to feeding by *S. exigua* larvae (Chung et al., 2008). Moreover, most of JA signaling genes like *AtJAZ1-10* were found to be upregulated on herbivore feeding. These results indicate a direct role of JAZ proteins in defense and plant development. In addition, recently, a few reports have also linked JA signaling with potassium (K) and Phosphorus (P) deficiency response (Chacón-López et al., 2011; Shankar et al., 2013, Takehisa et al., 2013; Wu et al., 2015).

Balanced mineral nutrients supply is critical for optimal growth and development of plants. Each mineral nutrient plays a critical role in the physiological and developmental aspects of plants (Marschner, 1995). Nutrient-deficiency responses are controlled by many factors including phytohormones. For example, cytokinins (CKs) negatively regulate the Pi (Phosphate) starvation response (Martín et al., 2000), abscisic acid (ABA) regulates both sulfur homeostasis and Pi-starvation responses (PSR) (Jiang and Zhang, 2001). While auxins seem to interact/regulate with signaling pathways for the homeostasis of many nutrients including nitrogen (N), phosphorus (P), sulfur (S), and potassium (K) (Franco-Zorrilla et al., 2004; Ticconi and Abel, 2004; Ashley et al., 2006; Kopriva, 2006; Zhang et al., 2007). Further, auxins regulate root hair and lateral root development under Pi deficiency to increase the root absorption area (López-Bucioet al., 2003). Cytokinins, on the other hand, regulate metabolic changes under nitrogen deficiency (Sakakibara, 2006). Therefore, phytohormones control both physiological and architectural adaptations for nutrient homeostasis.

JA is well-known for inhibiting root elongation (Staswick et al., 1992, Wasternack and Hause, 2013) and plays a key role in root meristem alteration under P deficiency (Chacón-López et al., 2011). Further, transcriptome analysis has revealed that many JA responsive genes including JA biosynthetic genes (*OsAOS1, OsLOX2*, and *OsLOX3*) and JAZ family genes (*OsJAZ2*, -*5*, and -*9*) are induced under K deficiency (Takehisa et al., 2013). As many nutritional deficiencies also modulate root system architecture (RSA) to enhance the acquisition of essential nutrients (Lynch, 2011), it becomes rational to study the behavior of JA signaling genes, especially *JAZ* repressors under both macro and micro nutrients deficiency. Previously, 12 JAZ proteins have been identified in *Arabidopsis thaliana* (Thines et al., 2007) while 15 in *Oryza sativa* (Ye et al., 2009), but there was no report for JAZ proteins in *Cicer arietinum*, a legume which is efficient in nutrient homeostasis (Schulze et al., 2006; Varshney et al., 2013). In this study, we have identified 10 JAZ proteins in the recently sequenced chickpea genome, examined their phylogenetic relationships and studied expression patterns of *JAZ* genes in rice and chickpea under macro (N, P, K) and micro (Fe, Zn) nutrients deficiency. Our results showed the structural and functional conservation of JAZ repressors in monocots and dicots, and their differential behavior under macro and micro mineral-deficiency suggested a potential role of JA in plant nutrient homeostasis.

## Material and methods

### Identification of JAZ proteins in rice and chickpea

Rice and Arabidopsis known JAZ proteins were obtained from previous studies (Vanholme et al., 2007; Ye et al., 2009). These protein sequences were then used as queries to search for potential JAZ proteins in other organisms, namely, *Physcomitrella patens, Brassica rapa, Linum usitatissimum, Zea mays, Medicago truncatula, Manihot esculenta, Populus trichocarpa, Ricinus communis, Solanum tuberosum*, and *Solanum lycopersicum* using BLASTP in their respective databases (http://phytozome.jgi.doe.gov/pz/portal.html). Protein sequences, so obtained, were scanned for the presence of TIFY and Jas domain using SMART (http://smart.embl-heidelberg.de/) and interpro (http://www.ebi.ac.uk/interpro/). Proteins with both TIFY and Jas domains were retained for further analysis. After removal of redundant hits, 165 unique proteins (Supplementary text 1) with both TIFY and Jas domain were aligned and a Hidden Markov Model (HMM) was generated using HMMER 3.0 (http://cryptogenomicon.org/2010/03/28/hmmer-3-0/). This HMM was then used for HMMER searches in the rice and chickpea protein databases. For chickpea, both desi (http://nipgr.res.in/CGAP/home.php) and kabuli (http://www.icrisat.org/gt-bt/ICGGC/homepage.htm) genomes were searched (*p* = e^−50^). All the protein sequences obtained were again searched for non-redundant hits and only unique hits were scanned for the presence of TIFY and Jas motifs. Final sequences were considered as potential JAZ proteins in rice and Chickpea.

### Structural analysis of CaJAZ and OsJAZ proteins and genes

The protein sequences were analyzed in SMART (http://smart.embl-heidelberg.de/) and aligned in ClustalX (http://www.ebi.ac.uk/Tools/msa/clustalo/) to confirm the presence of TIFY and Jas motifs. The TIFY (TIF(F/Y)XG) domain was extracted from SMART and INTERPRO databases while the Jas domain was identified manually from the CCT domain having SLX_2_FX_2_KRX_2_RX_5_PY as the conserved amino acid motif. MEME (Multiple Expectation Maximization for Motif Elicitation, Bailey et al., 2009) was used to further identify the additional motifs in identified rice and chickpea JAZs. For nucleotide level investigation, *JAZ* genes were visualized in rice genome database and cDNA and genomic sequences were aligned manually. Chickpea *JAZ* information, like chromosomal location, genomic DNA sequences, exon and intron structures, protein sequences and coding sequences, were obtained from the CGAP database (http://nipgr.res.in/CGAP/home.php). Phylogenetic trees were generated for JAZ protein sequences of *Arabidopsis thaliana* (12), *Oryza sativa* (15), *Physcomitrella patens* (4), *Brassica rapa* (24), *Linum usitatissimum* (8), *Zea mays* (27), *Medicago truncatula* (9), *Manihot esculenta* (19), *Populus trichocarpa* (10), *Ricinus communis* (7), *Solanum tuberosum* (14), *Solanum lycopersicum* (6), and *Cicer aeriantum* (10). All protein sequences were aligned with ClustalX and an unrooted phylogenetic tree was generated using MEGA 6.06 (Molecular Evolutionary Genetics Analysis), with the neighbor-joining method. Phylogenetic trees were visualized using MEGA 6.06 software with bootstrap values from 1000 replicates at each branch (Tamura et al., 2007).

### Promoter sequence analysis of *OsJAZ* and *CaJAZ* genes

To identify the putative *cis*-acting elements in a promoter region, 2 kb region upstream of the start codon was scanned in the Plant *Cis*-acting Regulatory DNA Elements database (http://www.dna.affrc.go.jp/PLACE/) for both rice and chickpea *JAZ*s.

### Ka/Ks analysis of *OsJAZ* and *CaJAZ* genes

For estimation of non-synonymous (Ka) and synonymous (Ks) substitution rates, the aligned amino acid sequences and their corresponding cDNA sequences of rice and chickpea *JAZ* genes conserved across the plant species, were analyzed using CODEML in the PAML interface tool of PAL2NAL (http://www.bork.embl.de/pal2nal).

### Plant growth conditions and different nutrient deficiency treatments

Rice (*Oryza sativa* var PB1) seeds were surface-sterilized by 0.1% mercuric chloride for 10 min and, thereafter, washed five-times with sterile water and germinated on wet filter paper for 2 days in the dark at 37°C. Uniformly germinated rice seedlings were transferred to liquid culture medium (Yoshida et al., 1976) with the following composition: NH_4_NO_3_(1.40 mM), NaH_2_PO_4_ (0.32 mM), K_2_SO_4_ (0.51 mM), CaCl_2_.2H_2_O (1 mM), MgSO_4_.7H_2_O (1.7 mM), H_3_BO_3_ (19 μM), ZnSO_4_.7H_2_O (0.15 μM), CuSO_4_.5H_2_O (0.15 μM), (NH4)_6_MO_4_O_2_.4H_2_O (0.015 μM), Citric Acid (70.75 μM), Na-Fe-EDTA (60 μM), and MnCl_2_.4H_2_O (9.46 μM). Seedlings were transferred to complete media for the control and nutrient solution carrying lower concentration of NH_4_NO_3_(14 μM) for N deficiency (-N), NaH_2_PO_4_ (3.2 μM)for P deficiency (-P), K_2_SO_4_(5.1 μM) for K deficiency, ZnSO_4_.7H_2_O (0.0015 μM) for Zn deficiency (-Zn), and Na-Fe-EDTA (0.6 μM) for Fe deficiency treatment. Seedlings were grown in a growth chamber maintained at 16 h photoperiod, 30/28°C day and night temperature, 280–300 μM photons/m^2^/s photon density and ~70% relative humidity.

Chickpea (var. ICC4958 desi) seeds were surface-sterilized with 70% ethanol for 2 min followed by 2% sodium hypochlorite carrying a drop of tween-20 treatment for 20 min. Thereafter, seeds were again surface-sterilized with 0.1% HgCl_2_ for 1 min and washed with sterile water 5 times to remove the surfactants. Surface-sterilized seeds were soaked in water overnight and then transferred to wet germination paper for 2 days in dark. Uniformly, germinated seedlings were transferred to aerated liquid culture medium (1/4th Hoagland) with 696.9 μM Ca(NO_3_)_2_, 1.02 μM MgSO_4_.7H_2_O, 1.5 μM KNO_3_, 459.9 μM H_3_BO_4_, 3.0 μM CuSO_4_, 63.05 μM MnCl_2_.4H_2_O, 1.38 μM Na_2_MoO_4_, 7.9 μM ZnSO_4_, 252.1 μM NaH_2_PO_4_.2H_2_O, 57.8 μM Na-Fe-EDTA, 252.1 μM NH_4_Cl for control. For N deficiency, seedlings were grown in Hoagland solution having KNO_3_ and CaNO_3_ replaced by equimolar concentration of K_2_SO_4_ and CaCl_2_, respectively. For P deficiency, seedlings were grown in Hoagland solution having 2.52 μM NaH_2_PO_4_. For K deficiency, seedlings were placed in nutrient medium supplemented with 0.01 μM K_2_SO_4_ and KNO_3_ was replaced by equimolar concentration of NH_4_NO_3._ For Zn and Fe deficiency seedlings were placed in nutrient solutions containing 0.07 μM ZnSO_4_ and 0.57 μM Na-Fe-EDTA, respectively. Chickpea seedlings were raised in a chamber maintained at 12/12 h photoperiod, 23/18°C, 200–300 μM photons/m^2^/s photon density and ~70% relative humidity. Media were changed after every 2 days and pH was maintained everyday around 5.5 for both rice and chickpea.

In order to study the JA-inducible expression of chickpea *JAZ* genes, 12-days-old seedlings were treated with 100 μM Methyl-Jasmonate in liquid growth media for variable periods. The experiment was performed with three biological replicates.

### Expression analysis of *JAZ* genes under different nutrient deficiencies in rice and chickpea roots

#### Sample collection, RNA extraction, and cDNA preparation

Root tissues were collected at 7 days (early response) and 15 days (late response) after stress treatment. Tissues were frozen immediately in liquid nitrogen for further analysis. Sample collection was done during 2–3 p.m. every time to minimize the possible circadian effects. Experiments were repeated in three biological replicates. Total RNA from root was extracted using the TRIzol® method according to manufacturer's instruction, and further treated with DNAse to avoid genomic DNA contamination. cDNA was synthesized from 1 μg total RNA using a High Capacity cDNA Reverse Transcription Kit (Applied Biosystems) according to manufacturer's instructions.

#### Primer designing and qRT-PCR

Primers for quantitative real-time PCR (qRT-PCR) were designed from coding region using PRIMER EXPRESS version 2.0 (PE Applied Biosystems™, USA) with default parameters. Each primer pair was checked for its specificity for its respective gene using BLAST tools of NCBI and TIGR databases. qRT-PCR was performed with cDNA using Fast SYBR® Green Master Mix to detect the quantity of double stranded product in Applied Bio systems 7500 Fast Real-Time PCR. Quantitative assays were performed in triplicates for each sample. The relative gene expression was calculated using the ^ΔΔCt^ method. *Ubiquitin5* (Os01g0328400) and Elongation Factor 1-alpha (AJ004960) were used as endogenous controls for rice and chickpea, respectively. A student's *t*-test was used for testing level of significance. Primer sequences for all the genes are listed in Table S1.

Tissue-specific expression patterns of CAJAZs were obtained from chickpea transcriptome database (CTDB; http://www.nipgr.res.in/ctdb.html) and the expression levels are provided as RPM (Reads per Million) values. While microarray data for *OsJAZs* was taken from rice expression database (http://www.ricearray.org/).

#### CaJAZ6 cloning and expression in onion epidermal cells

The *CaJAZ6* sequence, obtained from chickpea database (http://nipgr.res.in/CGAP/home.php), was used for primer designing to amplify the ORF. Amplified sequence was confirmed using DNA sequencing for accuracy, and cloned in entry vector (pENTR™-DTOPO®). The ORF was then moved into the binary vector, pSITE3CA using LR reaction to produce YFP:CAJAZ6 fusion protein. DNA-coated gold particles were used for particle bombardment in onion epidermal cells as described (Giri et al., 2011). CaJAZ6 was visualized as YFP:CAJAZ6 fusion protein under fluorescence microscope (Nikon eclipse 80i).

## Results

### *JAZ* genes in rice and chickpea

Fifteen and twelve *JAZ* genes were reported earlier in rice and Arabidopsis, respectively (Thines et al., 2007; Ye et al., 2009). The Chickpea genome, a dicot like Arabidopsis, has been sequenced recently (Jain et al., 2013; Varshney et al., 2013). After comprehensive data mining in the rice genome for JAZ proteins, we did not find any new members of the family. Whereas, in chickpea, we identified 10 JAZ proteins using the protein blast and HMM searches. The identified chickpea JAZs were named according to their homology with Arabidopsis JAZs (Table 1; Figure S1). The chromosomal localization of *JAZs* was analyzed with Oryzabase for rice. Fifteen *JAZs* were located on six chromosomes. Five *OsJAZ* genes were present on chromosome 3, two each on chromosome 4 and 7, one on chromosome 8, two on chromosome 9, and three on chromosome 10 (Figure S2). Expansion of the rice *JAZ* gene family was also aided by tandem gene duplication as five genes are located in two duplicated blocks (*OsJAZ9-11* on chr 3; *OsJAZ12-13* on chr 10). Chromosomal positioning of *CaJAZ* genes showed that *CaJAZ10* was present on chromosome 2, *CaJAZ3b* on chromosome 6, *CaJAZ6* on chromosome 7 while three genes, *CaJAZ1b, CaJAZ12b*, and *CaJAZ3a* were present on chromosome 8 (Table 1). Four genes (*CaJAZ3c, 1a, 12a*, and *8*) were present on scaffolds (scaffold02277, 03027, 03745, and 06768, respectively).

**Table 1:** **Structural and coding details of chickpea *JAZ* genes**.

**JAZ name**	**Protein ID**	**Chr**.	**Physical position**		**CTDB id**	**Domains/motifs**	**CDS length**	**No. of exons**	**aa length**	**pI**	**Protein MW (Kda)**	**AL**
CaJAZ10	Ca_01810.1	Ca_LG_2	12,357,154	12,363,824	TC11585	TIFY, Jas	901	8	300	6.73	32.94	1
CaJAZ3b	Ca_06739.1	Ca_LG_6	3,957,625	3,960,995	TC04335	TIFY, Jas	1021	7	346	8.71	36.421	3
CaJAZ6	Ca_07919.1	Ca_LG_7	8,049,102	8,052,009	TC17994	TIFY, Jas	625	5	208	9.78	23.2999	1
CaJAZ1b	Ca_08149.1	Ca_LG_8	3,475,024	3,476,860	TC10471	TIFY, Jas	676	5	225	9.35	24.48	1
CaJAZ12b	Ca_08181.1	Ca_LG_8	3,823,275	3,826,085	TC18764	TIFY, Jas	541	5	180	9.55	19.606	1
CaJAZ3a	Ca_08733.1	Ca_LG_8	10,385,422	10,391,976	TC05885	TIFY, Jas	1153	7	384	9.29	41.613	2
CaJAZ3c	Ca_17203.1	Scaffold02277	11,984	17,289	TC11159	TIFY, Jas	1009	7	336	9.21	35.339	3
CaJAZ1a	Ca_18081.1	Scaffold03027	3316	6294	TC11369	TIFY, Jas	769	5	256	9.11	27.732	1
CaJAZ12a	Ca_18578.1	Scaffold03745	8598	11,757	TC18345	TIFY, Jas	625	5	208	8.26	22.247	2
CaJAZ8	Ca_19748.1	Scaffold06768	4292	7024	-	TIFY, Jas	412	3	137	9.1	15.663	1

*CDS, coding sequence; aa, amino acids; MW, molecular weight; AL, alternate splicing*.

Intron number varies from 0-6 in rice *JAZ* genes. Three *OsJAZ* genes, namely, *OsJAZ9, OsJAZ10*, and *OsJAZ13* are intron-less (Table S2). The intron later-theory correlates the increased intron numbers with complex regulation and therefore, a more recent origin of gene (Roy and Gilbert, 2006). In rice *JAZ* genes, either *OsJAZ9* or *OsJAZ10* is the founder member since they lack intron and are duplicated partners with high sequence identity. *OsJAZ3* and *OsJAZ4* with highest number of introns (6) might have evolved recently (Figure S3). Surprisingly, we didn't find any *JAZ* without introns in chickpea. The lowest number of introns was in *CaJAZ8* (2 introns) (Table 1; Figure S3).

### Phylogenetic relationship and comparative analysis of JAZ proteins and genes

**Figure 1:**
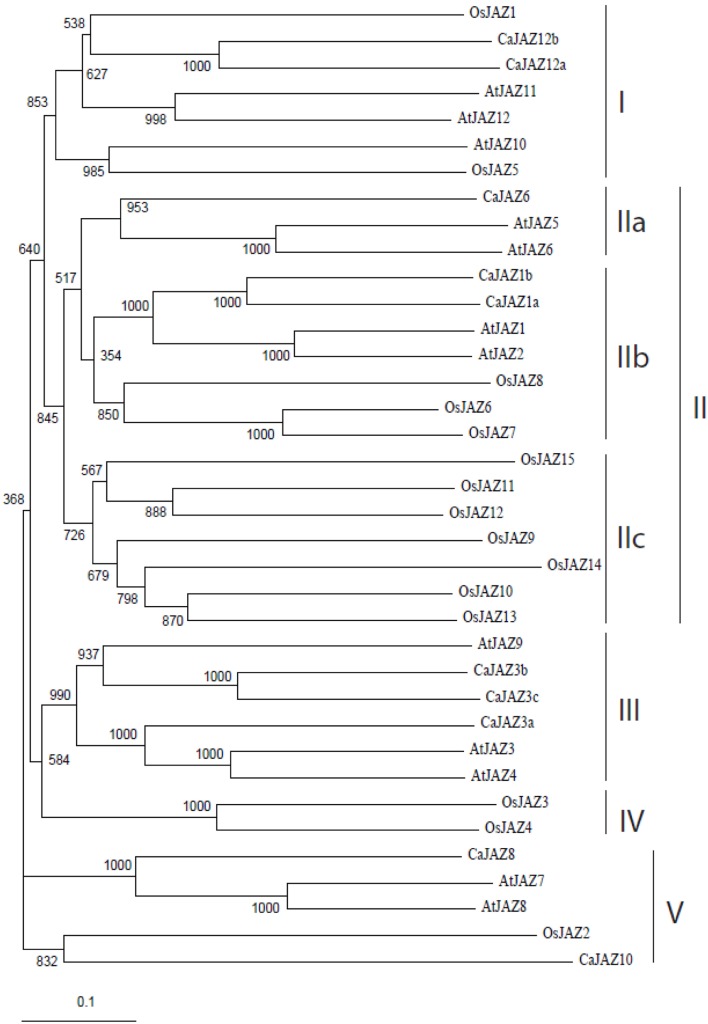
**Unrooted phylogenetic tree of rice, chickpea, and Arabidopsis JAZ proteins**. Amino acid sequences of JAZ proteins were aligned in Clustal X and phylogenetic tree was constructed using NJ method. Scale bar represents amino acid substitution rate, bootstrap values are mentioned at each node.

To study the phylogeny of JAZ proteins, N-J tree of rice, Arabidopsis and chickpea JAZs was analyzed and the reliability was tested by bootstrap analysis for 1000 replicates. Rice, chickpea and Arabidopsis JAZs formed five well-defined clades (bootstrap value >50%). Clade 3 was formed exclusively by chickpea and Arabidopsis proteins with bootstrap values greater than 93%. This clade comprised of AtJAZ3, 4, 9, and CaJAZ 3b, 3a, 3c revealing their homologous nature (Figure 1). Clade 1 also revealed the same result having chickpea and rice proteins in the same clade except OsJAZ1 and OsJAZ5 which showed homology with CaJAZ (12b and 12a) and AtJAZ (10 and 12), respectively. While Clade 2C contains exclusively OsJAZ proteins (which include OsJAZ9, 10, 11, 12, 13, 14, and 15) separating them from chickpea and Arabidopsis.

### Protein architecture of JAZ repressors in rice and chickpea

**Figure 2:**
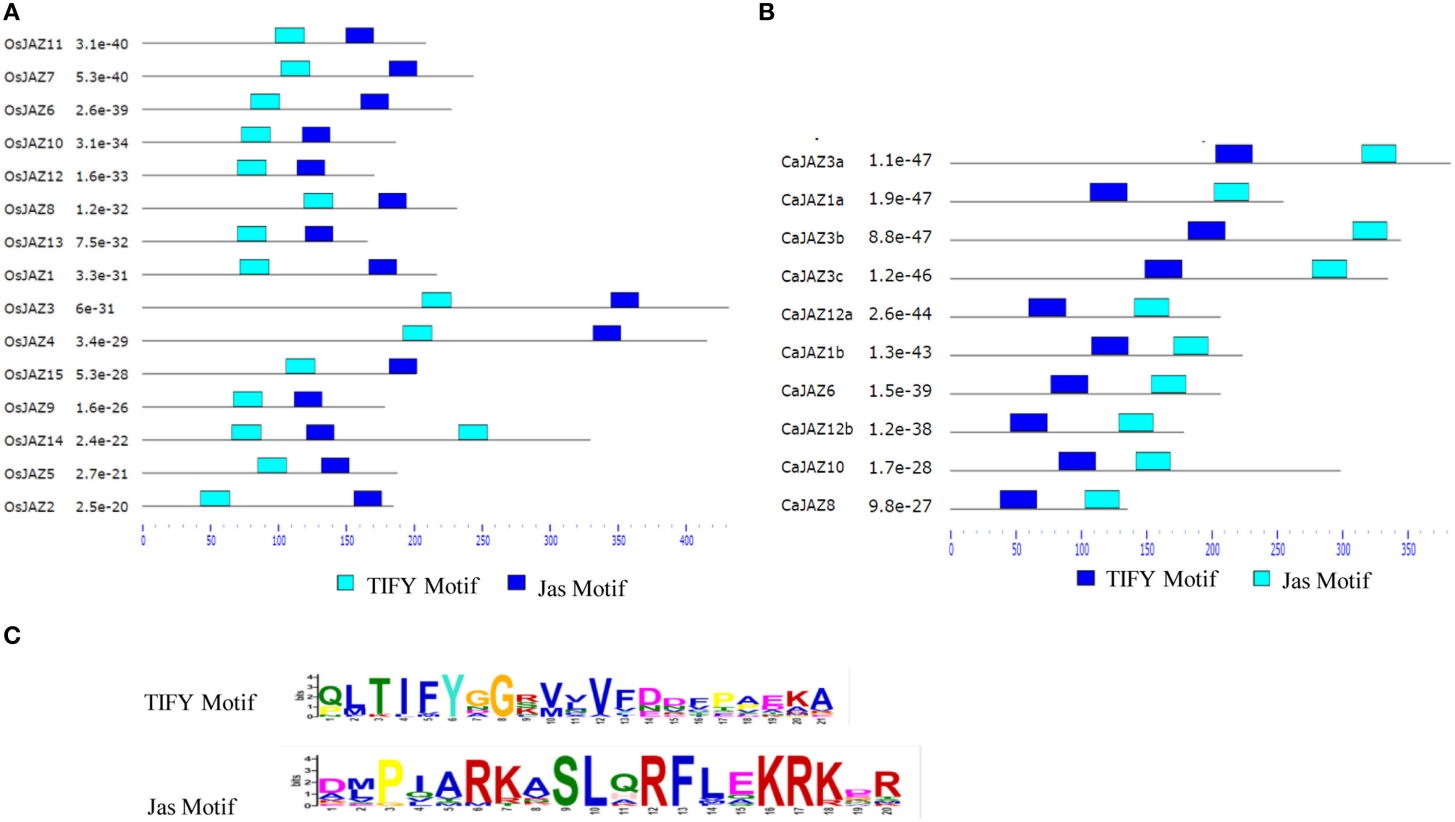
**Putative conserved motif distribution in (A) OsJAZ and (B) CaJAZ proteins**. Domains of OsJAZ and CaJAZ proteins were investigated using the MEME web server (www.meme-suite.org). Color blocks represent the position of motifs on corresponding proteins. **(C)** The consensus sequence of TIFY and Jaz motif from chickpea and rice JAZ proteins.

Arabidopsis JAZs possess the TIFY and Jas conserved domains at the N- and C-terminus, respectively. These domains are essential for their repressor activity. Therefore, we scanned the newly identified CaJAZs and rice JAZs in the MEME web server for the presence of these domains. Two putative conserved motifs TIFY (acc. PF06200) and Jas domain (acc. PF09425) were detected in both CaJAZ and OsJAZ proteins (Figure 2, Table S3). Single TIFY and Jas motifs were present in every protein except OsJAZ14 which contained two TIFY motifs. It was also observed that all JAZ proteins have TIFY domain at their N-terminus while Jas domain were at C-termini (Figure 2).

The TIFY domain contains 28 amino acids with a conserved TIF(F/Y)XG as core motif (Bai et al., 2011). However, we noticed a few variations of this core sequence in rice and Chickpea JAZs (CaJAZ10,-1b, -12b, -1a, -8; OsJAZ5, -14, -15). Therefore, we aligned TIFY domains from 165 JAZ proteins (identified from diverse organisms) and found that the TIFY motif has variable amino acids in different proteins. Out of 165 proteins, TIFY variations were found in 46 proteins, while three proteins lack TIFY. The remaining 116 proteins contain TIFYXG as a conserved motif (Figure 2C, Figure S4). The secondary-structure prediction analysis in Arabidopsis showed that the TIFY domain usually forms a beta-beta-alpha motif. Besides TIFY, the Jas motif is important for the interactions of JAZs with both MYC2 and COI-SCF E3 ubiquitin ligase complex for repressor degradation. It forms the JAZ degron and is characterized by a highly conserved SLX_2_FX_2_KRX_2_RX_5_PY consensus sequence and a conserved region of 5 amino acids (LPIAR as in AtJAZ1) at the N terminus (Sheard et al., 2010). The JAZ degron promotes JAZ-COI interaction (Shyu et al., 2012). It was further found that two basic amino acids (R, H, or K) in this region are very important for JA-Ile mediated COI-JAZ interaction; however, their absence does not affect the interaction of MYC2 with JAZ (Melotto et al., 2008). We found that these two basic amino acids are highly conserved in the loop region of Chickpea and rice Jas motifs (Figure S5).

### *cis*-acting elements in the promoter region of *JAZ* genes

To gain the further insights on the regulatory mechanisms of *JAZ* genes, their putative promoter region was analyzed for identification of binding sites of transcription factors involved in regulation of different nutrient stress responses. A variety of putative *cis*-elements were identified in rice *JAZ* genes. These include *P1BS*, a PHR1-binding sequence involved in regulation of PSR (phosphate starvation response) genes, IRO20S element which is an iron responsive element regulating iron responsive genes, GLMHVCHORD element, associated with nitrogen signaling and AMMORESIIUDCRNIA1 element which regulates the genes encoding nitrate reductase (Table S4). All nutrient deficiency related *cis*-acting elements were also present in *CaJAZ*, although variable in number, indicating a conservation of regulatory network for *JAZ* genes for nutrient deficiency between rice and chickpea (Table S5). Other than nutrient deficiency related *cis*-elements, both rice and chickpea JAZs genes upstream region also showed *cis*-elements related to development and environment stimuli (Tables S4, S5).

### Validation of *CaJAZ* as JA-responsive/signaling genes

**Figure 3:**
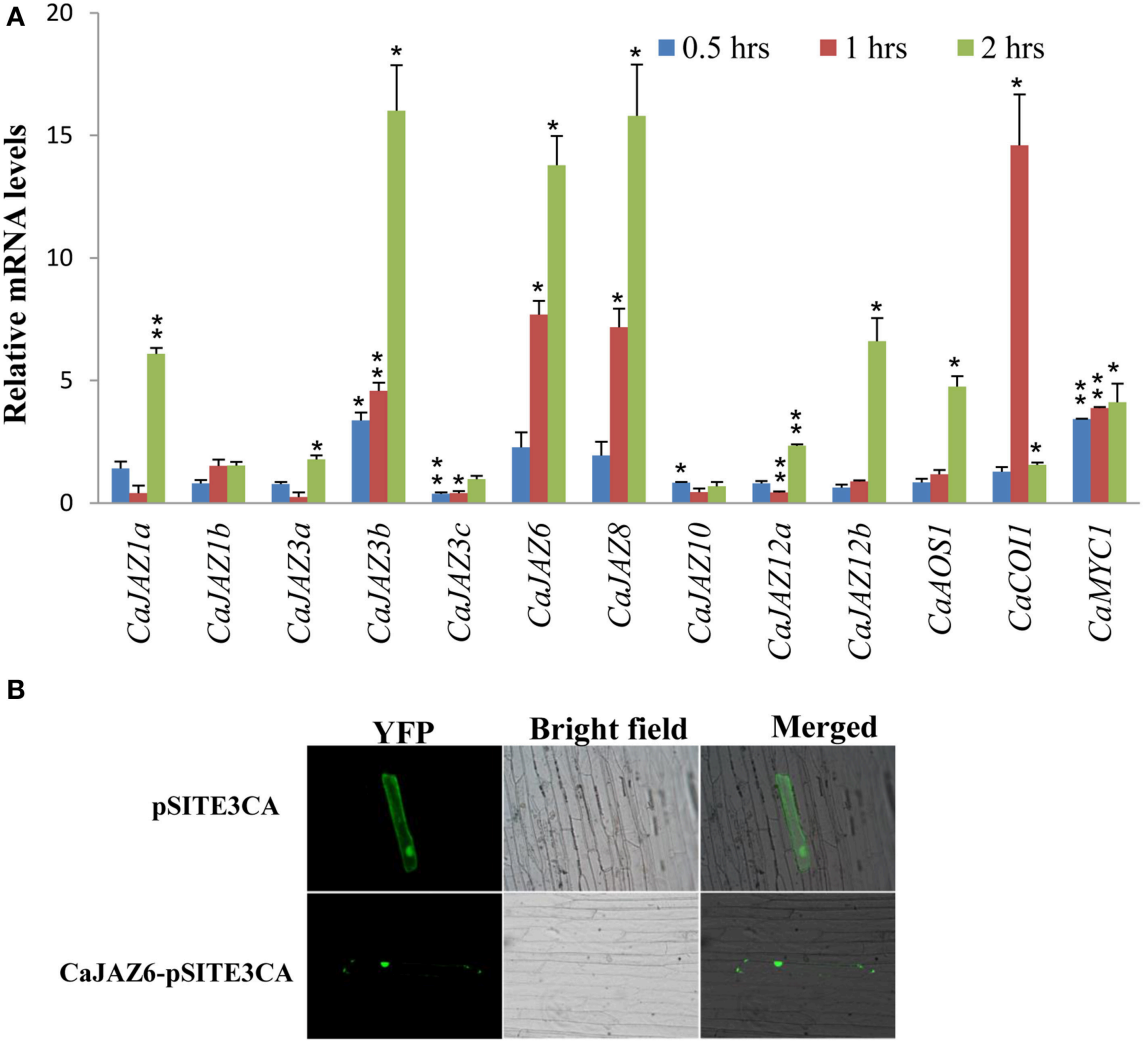
**Expression profile of *CaJAZs, CaAOS1, CaCOI1*, and *CaMYC1* genes in response to JA treatment (A) and subcellular localization of YFP:CaJAZ6 fusion protein (B)**. Expression was studied in methyl JA-treated root tissue using qRT-PCR. Chickpea *EF1-alpha* gene was used as endogenous control. Error bars indicate standard error of mean. Particle bombardment method was used for transforming the onion epidermal cells using DNA coated gold particles. Transformed cells were visualized under a florescence microscope. (^*^p < 0.05, ^**^p < 0.01).

**Figure 4:**
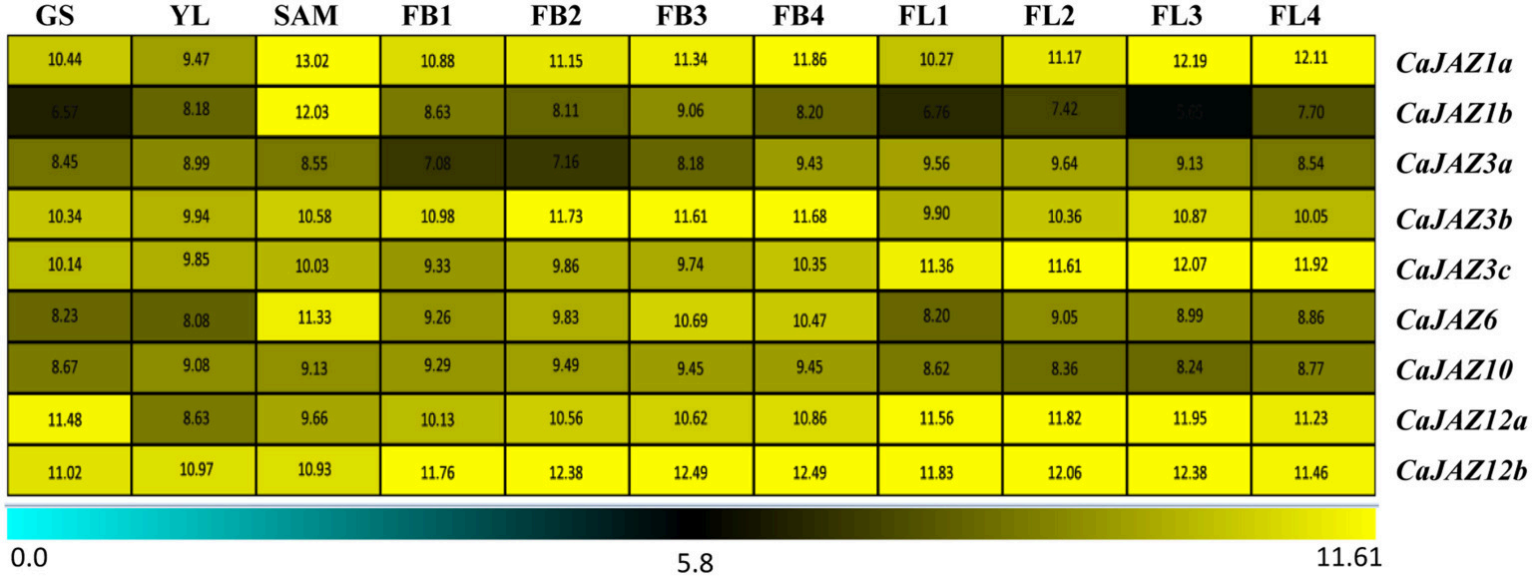
**Gene expression profile for *CaJAZ* genes in germinating seedling (GS), young leaf (YL), and 8 week stages of flower development [flower bud (4 mm; FB1), flower bud (6 mm; FB2), flower bud (8 mm; FB3), flower bud (8–10 mm; FB4), flower (unopened; FL1), flower (opened; FL2), flower (mature; FL3), and flower (drooped; FL4)]**. Expression data was retrieved from CTDB (http://www.nipgr.res.in/ctdb.html). Scale bar is showing relative transcript levels. Numbers on boxes show RAM (reads per million) values.

Both rice and Arabidopsis *JAZ*s showed transcriptional responses to JA-treatment (Chini et al., 2007; Ye et al., 2009). Our analysis on identified CaJAZs, showed presence of all conserved domains as reported in earlier known JAZs. Therefore, we analyzed the expression of *CaJAZ*s in response to JA for further confirmation. The expression levels of Jasmonate-associated genes (*CaJAZ, CaAOS1, CaCOI1*, and *CaMYC1* genes) were analyzed in response to JA treatment using qRT-PCR. As expected, all genes exhibited a differential expression pattern on treatment with Me-JA (Figure 3). We further confirmed the subcellular localization of YFP:CaJAZ6 fusion protein. As expected, YFP:CaJAZ6 localized to nucleus in onion epidermal cells (Figure 4), although some signal was also seen in membranes. This suggests that identified *JAZ* sequences in chickpea represent the true JAZ proteins and are involved in JA signaling.

### Expression profiling of *JAZs* genes in developmental stages and under different abiotic stresses

Tissue-specific expression profiling of *CaJAZs* showed their expression in all tissues, namely, shoot, root, mature leaf, flower bud, and young pod (Figure 4; Figure S6), indicating their vital roles in growth and development as reported earlier in rice and Arabidopsis (Cai et al., 2014). *CaJAZ12b* expressed in all the tissues. While *CaJAZ1b* and *-6* showed preferential expression in shoot apical meristem. *CaJAZ3a* and *-10* did not show high expression in any of the tissue. Their differential expression in different tissues indicates their possible roles in that particular tissue; however, some redundancy in the function still exists.

**Figure 5:**
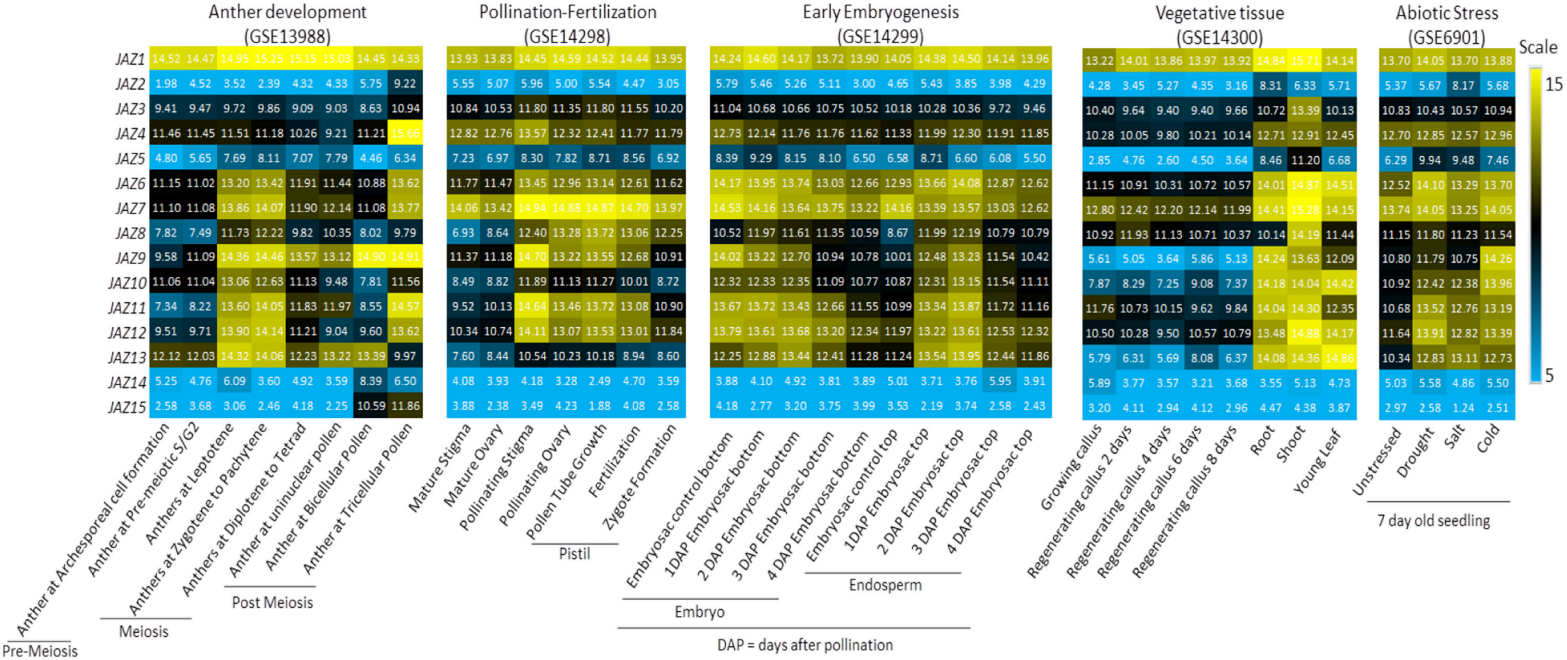
**Expression of rice JAZs in various developmental stages and in response to abiotic stress**. *JAZ* expression is examined during reproductive development, vegetative tissues, and under abiotic stress conditions. Reproductive stages are further divided into anther development, pollination to fertilization of pistil and early embryogenesis stages. Heatmaps are made on average log expression values obtained from rice Affymetrix expression data (indicated in white font). Experiment IDs of the microarray-experiments used for analysis are indicated on top of the heatmaps. Gene names for respective expression are shown on the extreme left.

Microarray expression data of *OsJAZs* indicate that the majority of them are differentially expressed in different tissues at reproductive and vegetative stages (Figure 5). These genes also showed responsiveness to cold, salt and drought stresses. All *OsJAZs* appear to have lower expression in callus cells. *OsJAZ1*, -**3**, and -**4** are highly expressed in most tissues, and are little affected by drought, salt, and cold. *OsJAZ2*, -**14**, and -**15** appear to be specific to late meiosis, though the first of these is also elevated in salt stress. *OsJAZ5* is most expressed in shoots but is elevated in roots in drought and salt stress, while *OsJAZ8* is shoot-specific and its root transcription is unaffected by the 3 stresses (Figure 5). Their expression patterns indicate the possible roles of JA in plant development and also in response to environmental stresses. Interestingly, most of the *JAZ*s were also expressed in roots. So far only *OsJAZ1* and *9* have been reported to play roles in root alteration and abiotic stress responses (Ye et al., 2009; Cai et al., 2014). Therefore, it would be interesting to delineate the different functions of remaining *JAZ* genes in plant growth and development as well as response to biotic and abiotic stresses.

### Expression profiles of *JAZ* genes under nutrient deficiency response

**Figure 6:**
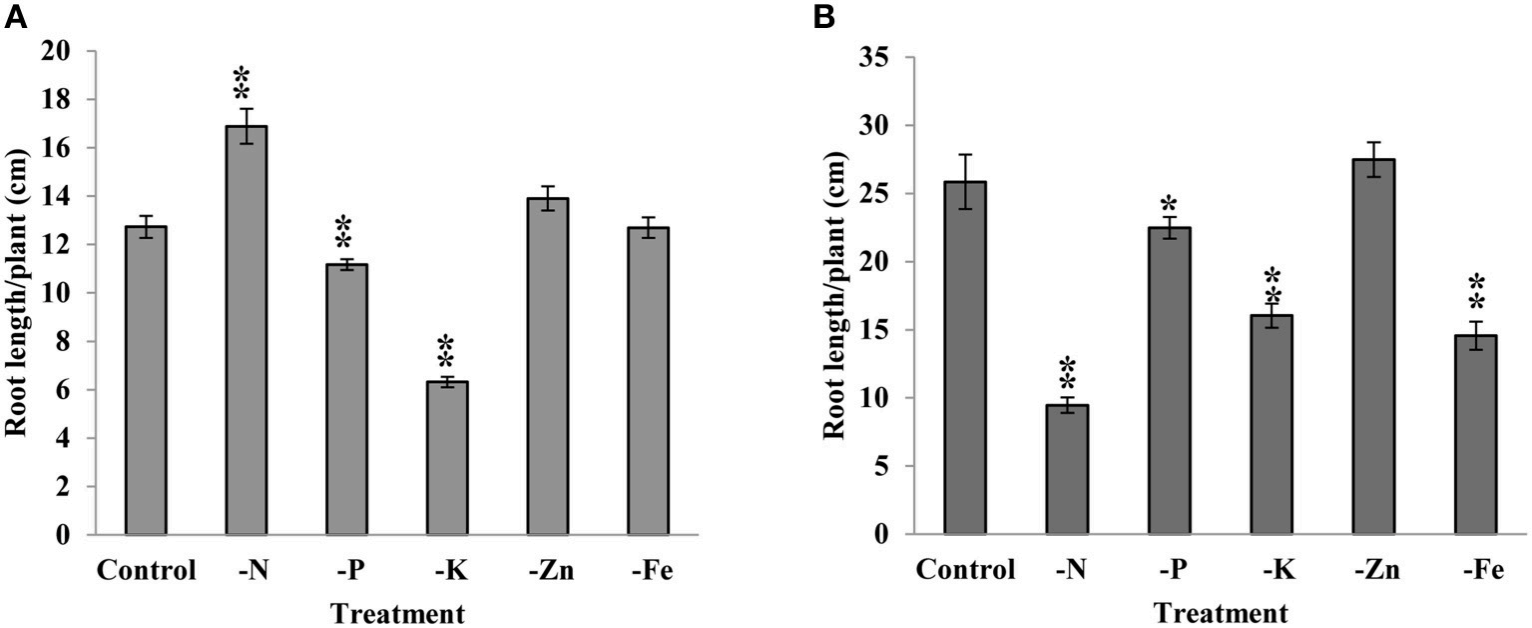
**Root length of rice (A) and Chickpea (B) under different nutrient stresses recorded after 15 days of treatment**. Length of longest root was used for measurement. Each bar represents the average of 20 plants/treatment with standard error. “^*^” indicates *p*-values.

Most of the nutrient deficiencies are sensed at root tips, which often lead to root-architecture modulation. Therefore, we studied the expression patterns of *JAZ* genes under selected macro and micro nutrients deficiency in root tissue. Root lengths of rice and chickpea were recorded after 15 days of growth under N, P, K, Fe, and Zn deficiency. We found significant decrease in root length under P and K deficiency and significant increase under N starvation in rice seedlings. However, chickpea root was significantly reduced under N, P, K, and Fe deficiency. Zn deficiency does not influence the root length of rice and chickpea (Figure 6). Expression patterns of *JAZs* were studies under these nutrients deficiency at early (7 days) and late (15 days) stages to get insights into their possible involvement in regulating the plant response.

#### N deficiency

**Figure 7:**
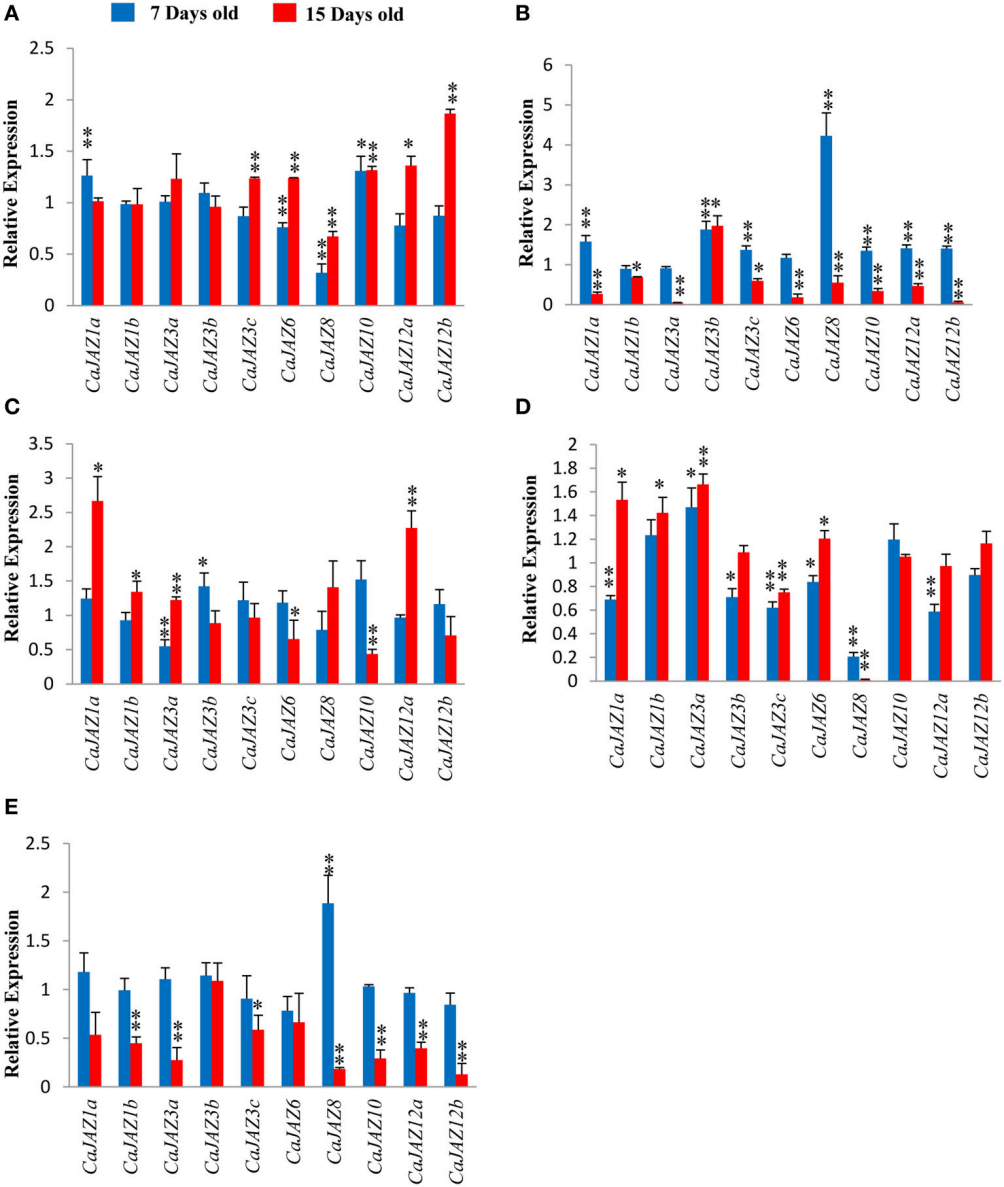
**Relative expression profile of *CaJAZ* genes under (A) N, (B) P, (C) K, (D) Fe, and (E) Zn deficiency after 7 (early response) and 15 days (late response) of the respective stress treatment in chickpea root**. qRT -PCR was used for quantitation of gene expression. Each bar shows the average of three biological replicates. Relative mRNA levels in treated plants were calculated using unstressed plants as control. *EF1-alpha* gene was taken as endogenous control. (^*^p < 0.05, ^**^p < 0.01).

**Figure 8:**
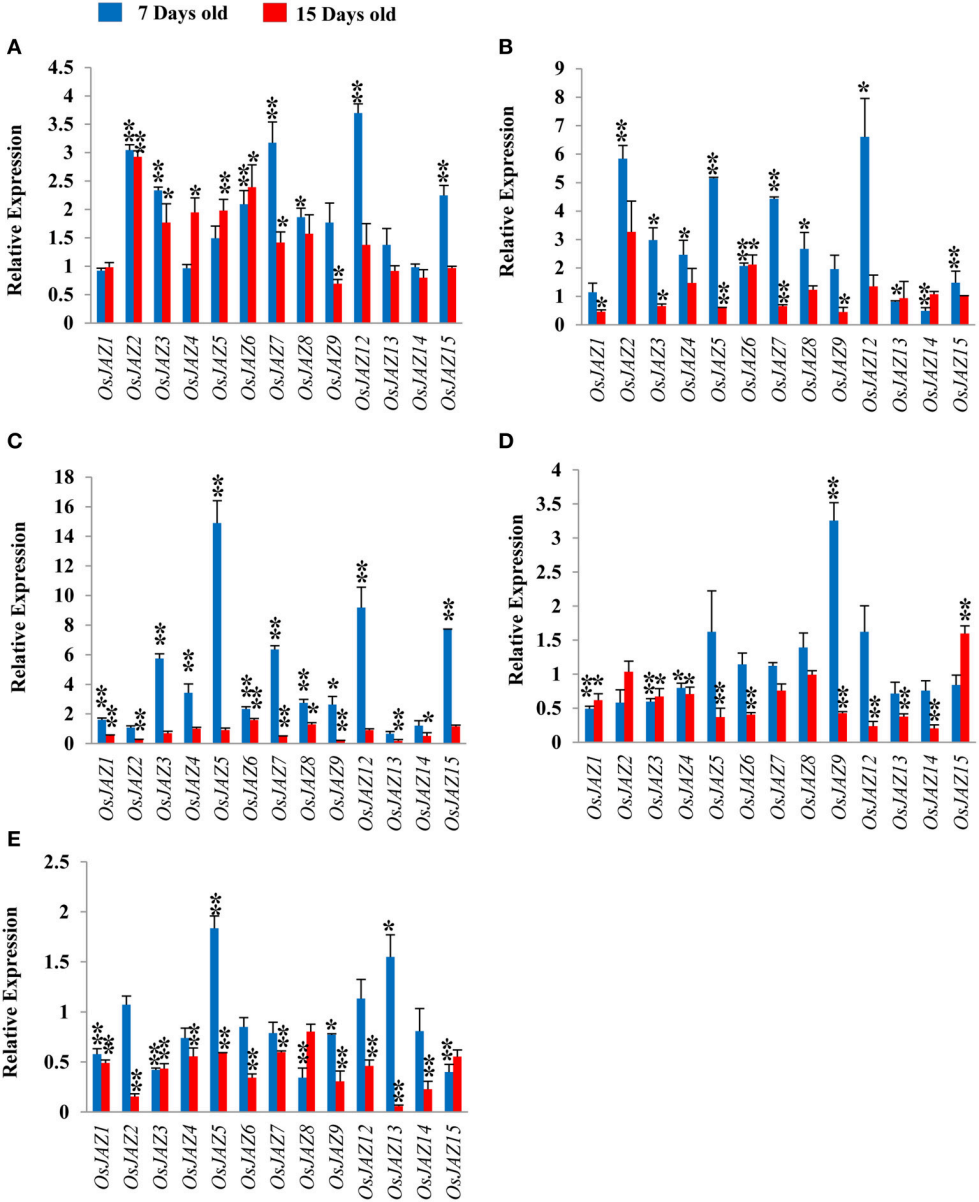
**Relative expression profile of *OsJAZ* genes under (A) N, (B) P, (C) K, (D) Fe, and (E) Zn deficiency after 7 (early response) and 15 days (late response) of respective stress treatment in rice root**. qRT-PCR was used for quantitation of gene expression. Each bar shows the average of three biological replicates. Relative mRNA levels in treated plants were calculated using unstressed plants as control. *Ubiquitin5* gene was taken as endogenous control. (^*^p < 0.05, ^**^p < 0.01).

Transcript profiling of chickpea *JAZs* showed upregulation of *CaJAZ10* and *CaJAZ1a* and downregulation for *CaJAZ6* and *CaJAZ8* (Figure 7) in response to early N deficiency. *CaJAZ10* was upregulated throughout the N starvation while *CaJAZ8* was found to be downregulated (Figure 7). Thus, *CaJAZ10* and *CaJAZ8* are both early and late responsive genes under N deficiency. We also found *CaJAZ6*, -**12b**, -**3c**, and -**12a** being upregulated at 15 days only, confirming them as late N deficiency responsive genes. Moreover, *CaJAZ3b*, -**1b**, -**3a** were unchanged throughout the experimental duration (Figure 7). In rice, *OsJAZ1* was not induced under N deficiency, however; most of the other *OsJAZ* genes were upregulated at both 7 days and 15 days (Figure 8).

#### P deficiency

P deficiency influenced the expression dynamics of most of chickpea and rice *JAZ* genes. *CaJAZ10, CaJAZ3b, CaJAZ12b, CaJAZ3c, CaJAZ1a, CaJAZ12a*, and *CaJAZ8* were upregulated under P deficiency at 7 days and none was downregulated. *CaJAZ3b* remained upregulated even after 15 days of low P stress while all other *CaJAZ* genes were significantly downregulated (Figure 7). Rice *JAZ* genes *OsJAZ13* and *OsJAZ14* were downregulated, *OsJAZ1* and *OsJAZ15* were non-responsive while all other *JAZ* genes were highly upregulated under P deficiency at 7 days of treatment (Figure 8). Interestingly, majority of them were also downregulated on 15th day of treatment.

#### K deficiency

Under K deficiency, most of *CaJAZ* genes were late responsive in nature. *CaJAZ3b* was upregulated while *CaJAZ3a* was downregulated at early stage (Figure 7). On the other hand, 4 genes were upregulated at 15 days. It is noteworthy that *CaJAZ1a* remains upregulated throughout the K deficiency. Many other genes also showed a trend of differential expression but it was statistically non-significant. However, most of rice *JAZ* genes were upregulated under K deficiency. Interestingly, rice genes followed a common pattern (Up- at 7 days followed by downregulation at 15 days; Figure 8). Noticeably, five genes *OsJAZ3*, -**4**, -**5**, -**8**, and -**15** were nonresponsive at 15 days of K deficiency, however; these genes were upregulated at 7 days of K deficiency. Therefore, *OsJAZ3, OsJAZ4, OsJAZ5, OsJAZ12*, and *OsJAZ15* were exclusively early responsive.

#### Fe deficiency

The expression analysis of chickpea *JAZs* showed that majority of genes (*CaJAZ3b*, -**6**, -**3c**, -**1a**, -**12a**, and -**8**) were downregulated at 7 days of Fe deficiency. Only *JAZ3a* was upregulated after 7 days and remained so even after 15 days (Figure 7). Besides *CaJAZ3a*, three more genes, namely, *CaJAZ6*, -*1b*, -*1a* also showed transcript induction at 15 days of deficiency, indicates their role in late response. Further, *CaJAZ3c* and -*8* remained downregulated throughout the experiment. Expression analysis of *OsJAZ* genes showed largely either downregulation or non-responsiveness at both the stages of Fe deficiency. Only *OsJAZ15* showed upregulation at 15 days stage. Although, a few genes (*OsJAZ5*, -**8**, -**9**, and -**12**) showed marginal upregulation at 7 days, they were again significantly downregulated at the late stage of stress. Further, most of the rice *JAZ* genes were downregulated at 15 days of Fe deficiency except three which were either unchanged (*OsJAZ2*, -**8**) or upregulated (*OsJAZ15*) after 15 days of Fe deficiency (Figure 8).

#### Zn deficiency

Expression analysis of Chickpea *JAZ* genes showed that all but one gene were unaffected at the 7th day of Zn deficiency, whereas *CaJAZ10*, -**1b**, -**12b**, -**3a**, -**3c**, -**12a**, and -**8** were downregulated at 15th days. We found only one gene (*CaJAZ8*) being upregulated at 7 days of Zn deficiency. Downregulation of most genes at 15 days of treatment indicated their involvement in late response (Figure 7). Expression analysis of most rice *JAZ* genes showed a high level of concordance with chickpea expression pattern under Zn deficiency. The majority of the genes were either downregulated or non-responsive at 7 days of Zn deficiency. Only *OsJAZ5* and *13* were upregulated at 7 days (Figure 8). Similarly, all rice *JAZs* were found downregulated in response to Zn deficiency at 15 days. Moreover, Zn deficiency has severely affected *OsJAZ2* (75% downregulation) and *OsJAZ13* (95% downregulation) which are the most downregulated genes in rice.

## Discussion

Plant adaptations to nutrient deficiency largely involve root architectural and physiological adjustments. We found significant decreases in rice root length under P and K deficiency and significant increases under N starvation. However, chickpea root length was significantly reduced under N, P, K, and Fe deficiency. It is noteworthy here that root elongation/reduction under nutrient deficiency is also dependent on genotype (Fageria et al., 1988). Noticeably, nutritional deficiencies also induce the biosynthesis of oxylipins and glucosinolates, as reported under K deficiency (Troufflard et al., 2010). These compounds are known precursors of JA; indicating roles for JA-mediated signaling in nutritional deficiency responses in plants. Further, most of the JA biosynthetic genes were found downregulated in a transcriptome study of the *lpi* (low phosphate insensitive) mutant (Chacón-López et al., 2011) in Arabidopsis. Application of Me-JA (Methyl Jasmonate) resulted in negative regulation of the expression of *FRO2* (Ferric Reduction Oxidase2), *IRT1* (Iron Regulated Transporter1) and *FIT* (Fer-like Iron deficiency induced Transcription factor) genes (Maurer et al., 2011), revealing a JA role in Fe deficiency responses. These studies indicate a role of JA in nutrient deficiency response in root. JAZs are repressor of JA signaling and also have nutrient responsive *cis*-element in their putative promoter regions. Therefore, we studied JAZ-gene behavior in the roots of rice and Chickpea as they serve the primary site for local sensing of nutrient availability in the surrounding environment.

### JAZ proteins in rice and chickpea

Rice and Arabidopsis contain 15 and 12 *JAZ* genes, respectively (Thines et al., 2007; Ye et al., 2009). While chickpea genome size is ~5 times bigger than Arabidopsis, the number of *JAZ* genes (10) identified is less, probably because the genome sequence is incomplete (Jain et al., 2013; Varshney et al., 2013). Although, JAZ varied in the composition of intron/exons, one intriguing feature of rice *JAZs* was the presence of long intergenic regions (LIR) implying a complex regulation of these JA-responsive genes (Figure S7). Their diverse expression patterns in development/stimuli-specific manners further support this notion. Out of three tandemly duplicated genes (*OsJAZ9*-**11**), -**9**, and -**10** lack introns but show opposite gene orientation (Jiang et al., 2013). *JAZ11* shared the gene orientation with *JAZ10* but had two introns. Further, *OsJAZ10* did not differentially express under nutrient deficiency. A similar discrepancy was observed for *JAZ13* and -*14*. This indicates an architectural and functional divergence in tandemly duplicated JAZs.

Almost all of the JAZs identified here contain conserved TIFY and Jas domain at their N- and C-terminal ends. The amino acid composition in the motifs is also largely conserved. TIFY domains mediate homo and hetero-dimerization interactions within JAZ proteins. It also mediates interaction between JAZ proteins and MYC transcription factors (Bai et al., 2011). The Jas motif is essential for JA-mediated receptor-repressor complex degradation for activation of JA signaling. The presence of these highly similar domains and architecture indicates the conserved nature of JAZ proteins in monocots and dicots, as reported in other gene families (Giri et al., 2013). Further, active JA is perceived by the JAZ-COI co-receptor complex and an alpha helix formed by the Jas degron may provide a low affinity anchor for JAZ proteins to dock on COI to form a JAZ-COI co-receptor complex. The substitution mutation of F (Phenylalanine) by A (alanine) in Jas degron disrupts JAZ1-COI1 interaction (Sheard et al., 2010). We found few JAZs with slight variations at Jas domain while OsJAZ14 encodes a truncated Jas domain. The multiple alignment of the Jas motif from various organisms (Supplementary text 1) also showed variable amino acid sequences. A comprehensive activity analysis of such JAZs would confirm the effects of these variations on JA-signaling. Further, the EAR motif which is involved in the regulation of JA-responsive genes via chromatin remodeling (Zhou et al., 2005; Wu et al., 2008; Berr et al., 2010) was also present in three rice JAZs (OsJAZ2, OsJAZ8, and OsJAZ13) and one chickpea (CaJAZ8). This further indicates the similar roles for rice and Chickpea JAZs in complex cellular signaling, mediated by JA.

Molecular phylogenetic analysis revealed that chickpea proteins are closer to Arabidopsis than that of rice. This could be due to the fact that both Arabidopsis and chickpea are dicots while rice is a monocot (Lee et al., 2011). In a phylogenetic tree of all 165 JAZ proteins from different organisms, rice, and chickpea JAZs were randomly distributed in different clades (Figure S8). While it shows the conservation of JAZ protein in diverse plants but a species level specification is not visible. The estimation of Ka/Ks substitution rates of rice and chickpea *JAZ* genes conserved across 11 other monocot and dicot species revealed that a larger fraction (~76%) of such genes contained Ka/Ks < 1.0, indicating a negative/purifying selection pressure (Table 2). The remaining conserved genes had Ka/Ks > 1.0 and thus are under positive selection pressure. This is in good concordance with the substitution ratio of non-synonymous to synonymous SNPs (Ka/Ks < 1.0) documented earlier in multiple plant species (Parida et al., 2012; Victoria et al., 2012; Varshney et al., 2013). The Ka/Ks was lowest in the *JAZ* gene-pairs conserved between *C. arietinum* and *M. truncatula* (0.29), followed by *O. sativa* and *Z. mays* (0.35) genes and highest between *C. arietinum* vs. *P. patens* (1.60). Collectively, the Ka/Ks estimates implicate the evolutionary closeness and divergence among 13 plant species based on rice and chickpea *JAZ* gene family, which is consistent with a number of previous studies (Lee et al., 2011; Zeng et al., 2014).

**Table 2:** **Ka/Ks measured in rice and chickpea *JAZ* genes conserved among 11 other plant species**.

**Plant species**	**Ka/Ks**	
	***Oryza sativa***	***Cicer arietinum***
*Ricinus communis*	1.1	0.77
*Zea mays*	0.35	0.98
*Solanum tuberosum*	1.21	0.73
*Solanum lycopersicum*	1.24	0.71
*Populus trichocarpa*	1.31	1.35
*Physcomitrella patens*	1.54	1.6
*Medicago truncatula*	0.93	0.29
*Manihot esculenta*	1.15	0.81
*Linum usitatissimum*	1.1	0.75
*Cicer arietinum*	0.95	1
*Brassica rapa*	1.08	0.7
*Arabidopsis thaliana*	0.89	0.65
*Oryza sativa*	1	0.95

All *CaJAZs* exhibited a differential expression on Me-JA treatment. Three genes, namely, *CaJAZ3b*, -**6**, and -**8** also showed a similar expression maxima with *CaCOI1*. However, induction of JAZs by JA alone does not confirm their roles in JA signaling as JAZs with truncated JAs domain are also induced by JA (Ye et al., 2009). Nuclear localization of CaJAZ6, as also reported for rice and Arabidopsis JAZs, and the presence of highly conserved domains further confirmed the true nature of CaJAZ and their role in JA signaling.

A variety of potential *cis*-elements are present in 2 kb upstream region of *JAZ* genes. Few of them are known to be involved in nutrient deficiency responsive gene expression. Presence of these nutrient responsive motifs also encouraged us to explore the differential expression analysis of *JAZ* repressors under different nutrient stresses. *P1BS* element associated with low P responsive genes (Sobkowiak et al., 2012) was detected in six genes (*OsJAZ1*, -*2*, -*4*, -*6*, -*11*, and -*14*) promoter sequences. Noticeably, *OsJAZ2*, -*4*, and -**6** (having 4 copies of *P1BS* elements) showed significant induction under low P while *OsJAZ11* having only one copy did not express under low P. Similarly, role of copy number was also observed for CaJAZs under P stress. Further, the presence of AMMORESIIUDCRNIA1 and GLMHVCHORD elements, related to nitrogen signaling (Loppes and Radoux, 2001) in *OsJAZ2* and *OsJAZ3*, corroborates their higher upregulation under N deficiency in rice. Interestingly, all *OsJAZ* containing IRO20S elements (Ogo et al., 2006) were significantly downregulated after 15 days of Fe deficiency. Moreover, JA application is known to downregulate the Fe deficiency responsive genes. Therefore, JAZs may be involved in this process. However, the intricate connections between Fe deficiency responsive marker genes and JA signaling machinery need to be established via interaction studies between JAZ repressors and IRO2 genes (a bHLH transcription factor) through ChIP-PCR or EMSA assay to further understand Fe homeostasis in rice.

### Expression of rice and chickpea *JAZs* under mineral nutrient deficiency

Both rice and chickpea *JAZs* showed significant differential expression under five selected nutrient's deficiencies, and a few common trends also emerged between them (Table S6). They are induced early, and suppressed at a later stage of P deficiency. Similarly, rice JAZs followed an initial up- and later down regulation pattern under K deficiency. *CaJAZs* on the other hand, showed a late upregulation. Interestingly, a P and Fe interaction was observed, JAZs that were induced by P were largely suppressed under Fe deficiency. While N deficiency induced their expression at both early and late stages, they were commonly suppressed under Zn deficiency. This high degree of similarity between rice and chickpea JAZs in terms of expression indicates their functional conservation between monocot and dicot. This further strengthens their emerging role in regulation of nutrient deficiency response in plants (Wu et al., 2015). Interestingly, tandemly duplicated rice *JAZs* followed different expression under these conditions; indicating on sub- or neo-functionalization as observed for other gene families (Vij et al., 2008).

Soil N deficiency is one of the severe problems to derail the overall plant growth and yield (Hirel et al., 2007; Kraiser et al., 2011). Among the various adaptations, RSA alteration through decrease in primary root length and increase in lateral root elongation has been observed (López-Bucioet al., 2003; Gruber et al., 2013). The decreased primary root length in chickpea here reflects the behavior reported in Arabidopsis under N deficiency (Gruber et al., 2013). However, increased root length of rice under N deficiency is also in agreement with an earlier report (Zhang et al., 2015). Further, N deficiency also induces the accumulation of JA in maize seedlings (Schmelz et al., 2003). In a recent study, JA biosynthetic genes like *OPR1, LOX5*, and *AOS* were differentially expressed under N deficient conditions in barley (Comadira et al., 2015). In our experiments, *OsJAZs* were also upregulated under N starvation irrespective of stress duration. The upregulation of *OsJAZ* genes pertains to the repression of JA signaling which in turn can increase the root elongation for N uptake. Chickpea has a different mechanism of N homeostasis which might be causing its different root behavior. However, functional validation of this hypothesis would delineate the molecular mechanisms of root elongation through JAZ repressors under N deficiency in rice. Our co-expression analysis revealed that *CaJAZ12b* and *CaJAZ12a* co-express under N, P, and Zn deficiency which strongly correlates to their high degree of sequence similarity and noticeably their branch length is very close to each other in the phylogenetic tree. Similarly in rice, two groups of *OsJAZs* (*OsJAZ3*, -**4**, and *OsJAZ6*, -**7**) having high sequence similarity showed similar expression patterns under N, K, and Fe deficiency and are nearest neighbors, phylogenetically.

Under P deficiency we found a significant decrease in primary root length of chickpea which corresponds to the Arabidopsis phenotype (Pandey et al., 2013). P deficiency inhibits the primary root growth, increases lateral root length and enhances root hair length and density (Lynch, 2011; Niu et al., 2012). These adaptations increase the root surface area to enhance the P acquisition. JA signaling through its downstream components is also known to influence the RSA (Troufflard et al., 2010). In our expression analysis most of the *CaJAZ* and *OsJAZ* genes were upregulated at 7 days of low P stress; however, as the stress was prolonged for 15 days, expression of most of JAZs were subsided. This may be due to the fact that root undergoes rapid local sensing under P deficiency as it comes in contact with low P containing medium (Svistoonoff et al., 2007). *JAZ* genes are also induced transiently and degraded quickly while conveying the signal to downstream genes (Thines et al., 2007). These expression patterns of *JAZs* were also evident in N and K macronutrient deficiency in rice, indicating a potential role for them in low P signaling.

K in soil combines with silicates and other molecules to form insoluble compounds and become unavailable to plants (Gruber et al., 2013; Meena et al., 2014). Root growth inhibition is a well-known response to both K deficiency and JA application (Troufflard et al., 2010). It has been reported that many JA biosynthetic genes like Lipoxygenase (*LOX*), Allene Oxide Synthase (*AOS*), and Allene Oxide Cyclase (*AOC*) were upregulated under K deficiency (Armengaud et al., 2004; Troufflard et al., 2010; Shankar et al., 2013; Takehisa et al., 2013), suggesting an active role of JA in root growth development and architecture modulation under K deficiency. We also found similar root growth inhibition of chickpea and rice seedlings under K deficiency. However, any role of JAZ repressors remains largely elusive. In earlier transcriptome studies of rice root under K deficiency, *OsJAZ9* was downregulated while *OsJAZ12* and -**13** were found to be upregulated (Takehisa et al., 2013). We also found similar behavior of *OsJAZ9* and *OsJAZ12* in our experiments. It is noteworthy that most *OsJAZs* were upregulated after 7 days of all three macronutrient (N, P, K) deficiency. However, the induction of *OsJAZs* was highest under K deficiency. This indicates that they are early responsive and suppress the JA signaling to alter the RSA of the rice for increasing nutrient uptake. Further, *OsJAZ6* is upregulated at an approximately constant level under NPK deficiency throughout the experiment. This gene is an interesting candidate for N, P, and K starvation studies involving the JA machinery. Chickpea on the other hand, showed mixed expression patterns and therefore, needs to be investigated further.

Being an important constituent of the electron transport system, iron (Fe) deficiency affects the plant growth and yield (Kobayashi and Nishizawa, 2012; Maathuis and Diatloff, 2013). We found a significant reduction in primary root length of chickpea under Fe deficiency, which is in agreement with earlier observations in Arabidopsis (Gruber et al., 2013). It has been further reported that P deficiency enhances Fe availability in root and shoot (Zheng et al., 2009; Rai et al., 2015). Interestingly, we also noticed that while JAZs were induced by low P they were largely suppressed under Fe deficiency. However, the actual role of JAZs remains to be investigated in this interaction. Most of the rice *JAZ* genes are either down regulated or nonresponsive under Fe deficiency. Noticeably, it has been reported that JA application suppresses the Fe deficiency responsive marker genes *IRT1, FRO2*, and *FIT* in Arabidopsis (Maurer et al., 2011). This behavior of *OsJAZs* indicates that Fe deficiency negatively affects the regulation of JA signaling. In our expression analysis most of the rice and chickpea *JAZ* genes were down regulated at 15 days of Zn deficiency. Incidentally, JA accumulation under Zn deficiency has been reported in *Sorghum bicolor* (Li et al., 2013). Therefore, we tested the expression level of JA biosynthetic genes *OsAOS2* and *CaAOS1* under Zn deficiency (Figure S9). While *CaAOS1* was upregulated at both 7 days and 15 days of Zn deficiency, *OsAOS1* showed marginal upregulation at 7 days but downregulated at 15 days stage. Although it supports our hypothesis to a large extent, quantification of JA in rice and Chickpea under Zn deficiency would reveal the role of JAZs. Nevertheless, these observations collectively indicate the probable roles of JAZ repressors in nutrient signaling in chickpea and rice.

OsJAZ2 showed high sequence similarity with CaJAZ10. Interestingly, these two genes followed almost identical expression patterns under all nutrient deficiencies. This makes OsJAZ2 a true homolog of CaZAJ10. However, OsJAZ1 lies in the same clade with CaJAZ12b and 12a but they did not follow similar expression trends suggesting a divergent regulation. Both rice and chickpea *JAZs* also showed diverse organ- and tissue-specific expressions. Many of them are also induced by cold, salt and drought stress. OsJAZ9 and AtMYC2 have already been shown to regulate the salinity and drought stress tolerance (Abe et al., 2003; Seo et al., 2011; Wu et al., 2015). Therefore, it would be interesting to investigate the role of JAZs in molecular coordination between JA and these abiotic stresses.

## Conclusions

We have identified 10 *JAZ* repressor genes from the newly sequenced chickpea genome and investigated their roles in mineral nutrient deficiency response. Early induction of *JAZ*s in root indicates their signaling roles leading to the adaptation to nutrient deficiency, probably via RSA alterations. Our findings add to the emerging roles of JAs in plant nutrient deficiency response via JAZ repressors, and provide a novel resource to study its applications in crop improvement.

## Author contributions

AS and BP conducted the experiments and wrote the manuscript. JG conceived the idea, designed the project, analyzed data, and wrote the manuscript. PD did microarray based expression analysis and helped in manuscript writing. LN helped in conducting various experiments. SP contributed to concept and helped in manuscript writing.

### Conflict of interest statement

The authors declare that the research was conducted in the absence of any commercial or financial relationships that could be construed as a potential conflict of interest.

## References

[B1] Abe H. Urao T. Ito T. Seki M. Shinozaki K. Yamaguchi-Shinozaki K. (2003). Arabidopsis AtMYC2 (bHLH) and AtMYB2 (MYB) function as transcriptional activators in abscisic acid signaling. Plant Cell 15, 63–78. 10.1105/tpc.006130 12509522 PMC143451

[B2] Armengaud P. Breitling R. Amtmann A. (2004). The potassium-dependent transcriptome of *Arabidopsis* reveals a prominent role of jasmonic acid in nutrient signalling. Plant Physiol. 136, 2556–2576. 10.1104/pp.104.046482 15347784 PMC523322

[B3] Ashley M. K. Grant M. Grabov A. (2006). Plant responses to potassium deficiencies: a role for potassium transport proteins. J. Exp. Bot. 57, 425–436. 10.1093/jxb/erj034 16364949

[B4] Bai Y. Meng Y. Huang D. Qi Y. Chen M. (2011). Origin and evolutionary analysis of the plant-specific TIFY transcription factor family. Genomics 98, 128–136. 10.1016/j.ygeno.2011.05.002 21616136

[B5] Bailey T. L. Boden M. Buske F. A. Frith M. Grant C. E. Clementi L.. (2009). MEME SUITE: tools for motif discovery and searching. Nucl. Acids Res. 37, W202–W208. 10.1093/nar/gkp335 19458158 PMC2703892

[B6] Berr A. McCallum E. J. Alioua A. Heintz D. Heitz T. Shen W. H. (2010). Arabidopsis histone methyltransferase SET DOMAIN GROUP8 mediates induction of the jasmonate/ethylene pathway genes in plant defense response to necrotrophic fungi. Plant Physiol. 154, 1403–1414. 10.1104/pp.110.161497 20810545 PMC2971616

[B7] Cai Q. Yuan Z. Chen M. Yin C. Luo Z. Zhao X.. (2014). Jasmonic acid regulates spikelet development in rice. Nat. Commun. 5, 3476. 10.1038/ncomms4476 24647160

[B8] Chacón-López A. Ibarra-Laclette E. Sánchez-Calderón L. Gutiérrez-Alanís D. Herrera-Estrella L. (2011). Global expression pattern comparison between low phosphorus insensitive 4 and WT Arabidopsis reveals an important role of reactive oxygen species and jasmonic acid in the root tip response to phosphate starvation. Plant Signal. Behav. 6, 382–392. 10.4161/psb.6.3.14160 21368582 PMC3142420

[B9] Chini A. Fonseca S. Chico J. M. Fernández-Calvo P. Solano R. (2009). The ZIM domain mediates homo- and heteromeric interactions between Arabidopsis JAZ proteins. Plant J. 59, 77–87. 10.1111/j.1365-313X.2009.03852.x 19309455

[B10] Chini A. Fonseca S. Fernández G. Adie B. Chico J. M. Lorenzo O.. (2007). The JAZ family of repressors is the missing link in jasmonate signalling. Nature 448, 666–671. 10.1038/nature06006 17637675

[B11] Chung H. S. Howe G. A. (2009). A critical role for the TIFY motif in repression of jasmonate signaling by a stabilized splice variant of the JASMONATE ZIM-domain protein JAZ10 in *Arabidopsis*. Plant Cell 21, 131–145. 10.1105/tpc.108.064097 19151223 PMC2648087

[B12] Chung H. S. Koo A. J. K. Gao X. Jayanty S. Thines B. Jones A. D.. (2008). Regulation and function of Arabidopsis *JASMONATE ZIM*-domain genes in response to wounding and herbivory. Plant Physiol. 146, 952–964. 10.1104/pp.107.115691 18223147 PMC2259048

[B13] Comadira G. Rasool B. Karpinska B. Morris J. Verrall S. R. Hedley P.. (2015). Nitrogen deficiency in barley (*Hordeum vulgare*) seedlings induces molecular and metabolic adjustments that trigger aphid resistance. J. Exp. Bot. 66, 3639–3655. 10.1093/jxb/erv276 26038307 PMC4463806

[B14] Fageria N. K. Wright R. J. Baligar V. C. (1988). Rice cultivar evaluation for phosphorus use efficiency. Plant Soil 111, 105–109. 10.1007/BF02182043

[B15] Feys B. J. F. Benedetti C. E. Penfold C. N. Turner J. G. (1994). Arabidopsis mutants selected for resistance to the phytotoxin coronatine are male sterile, insensitive to methyl jasmonate and resistant to a bacterial pathogen. Plant Cell 6, 751–759. 10.1105/tpc.6.5.751 12244256 PMC160473

[B16] Franco-Zorrilla J. M. González E. Bustos R. Linhares F. Leyva A. Paz-Ares J. (2004). The transcriptional control of plant responses to phosphate limitation. J. Exp. Bot. 55, 285–293. 10.1093/jxb/erh009 14718495

[B17] Giri J. Dansana P. K. Kothari K. S. Sharma G. Vij S. Tyagi A. K. (2013). SAPs as novel regulators of abiotic stress response in plants. BioEssays 35, 639–648. 10.1002/bies.201200181 23640876

[B18] Giri J. Vij S. Dansana P. K. Tyagi A. K. (2011). Rice A20/AN1 zinc-finger containing stress-associated proteins (SAP1/11) and a receptor-like cytoplasmic kinase (OsRLCK253) interact via A20 zinc-finger and confer abiotic stress tolerance in transgenic Arabidopsis plants. New Phytol. 191, 721–732. 10.1111/j.1469-8137.2011.03740.x 21534973

[B19] Gruber B. D. Giehl R. F. Friedel S. von Wirén N. (2013). Plasticity of the Arabidopsis root system under nutrient deficiencies. Plant Physiol. 163, 161–179. 10.1104/pp.113.218453 23852440 PMC3762638

[B20] He Y. Fukushige H. Hildebrand D. F. Gan S. (2002). Evidence supporting a role of jasmonic acid in Arabidopsis leaf senescence. Plant Physiol. 128, 876–884. 10.1104/pp.010843 11891244 PMC152201

[B21] Hirel B. Le Gouis J. Ney B. Gallais A. (2007). The challenge of improving nitrogen use efficiency in crop plants: towards a more central role for genetic variability and quantitative genetics within integrated approaches. J. Exp. Bot. 58, 2369–2387. 10.1093/jxb/erm097 17556767

[B22] Jain M. Misra G. Pate R. K. Priya P. Jhanwar S. Khan A. W.. (2013). A draft genome sequence of the pulse crop chickpea (*Cicer arietinum* L.). Plant J. 74, 715–729. 10.1111/tpj.12173 23489434

[B23] Jiang M. Zhang J. (2001). Effect of abscisic acid on active oxygen species, antioxidative defence system and oxidative damage in leaves of maize seedlings. Plant Cell Physiol. 42, 1265–1273. 10.1093/pcp/pce162 11726712

[B24] Jiang S. Y. González J. M. Ramachandran S. (2013). Comparative genomic and transcriptomic analysis of tandemly and segmentally duplicated genes in rice. PLoS ONE 8:e63551. 10.1371/journal.pone.0063551 23696832 PMC3656045

[B25] Kazan K. Manners J. M. (2012). JAZ repressors and the orchestration of phytohormone crosstalk. Trends Plant Sci. 17, 22–31. 10.1016/j.tplants.2011.10.006 22112386

[B26] Kobayashi T. Nishizawa N. K. (2012). Iron uptake, translocation, and regulation in higher plants. Annu. Rev. Plant Biol. 63, 131–152. 10.1146/annurev-arplant-042811-105522 22404471

[B27] Kopriva S. (2006). Regulation of sulfate assimilation in Arabidopsis and beyond. Ann. Bot. 97, 479–495. 10.1093/aob/mcl006 16464881 PMC2803671

[B28] Kraiser T. Gras D. E. Gutiérrez A. G. González B. Gutiérrez R. A. (2011). A holistic view of nitrogen acquisition in plants. J. Exp. Bot. 62, 1455–1466. 10.1093/jxb/erq425 21239377 PMC3137434

[B29] Lee E. K. Cibrian-Jaramillo A. Kolokotronis S. O. Katari M. S. Stamatakis A. Ott M.. (2011). A functional phylogenomic view of the seed plants. PLoS Genet. 12:e1002411. 10.1371/journal.pgen.1002411 22194700 PMC3240601

[B30] Li Y. Zhang Y. Shi D. Liu X. Qin J. Ge Q.. (2013). Spatial-temporal analysis of zinc homeostasis reveals the response mechanisms to acute zinc deficiency in Sorghum bicolor. New Phytol. 200, 1102–1115. 10.1111/nph.12434 23915383

[B31] López-Bucio J. Cruz-Ramírez A. Herrera-Estrella L. (2003). The role of nutrient availability in regulating root architecture. Curr. Opin. Plant Biol. 6, 280–287. 10.1016/S1369-5266(03)00035-9 12753979

[B32] Loppes R. Radoux M. (2001). Identification of short promoter regions involved in the transcriptional expression of the nitrate reductase gene in *Chlamydomonas reinhardtii* Plant. Mol. Biol. 45, 215–227. 10.1023/A:1006401312916 11289512

[B33] Lynch J. P. (2011). Root phenes for enhanced soil exploration and phosphorus acquisition: tools for future crops. Plant Physiol. 156, 1041–1049. 10.1104/pp.111.175414 21610180 PMC3135935

[B34] Maathuis F. J. Diatloff E. (2013). Roles and functions of plant mineral nutrients. Methods Mol. Biol. 953, 1–21. 10.1007/978-1-62703-152-3_1 23073873

[B35] Marschner H. (1995). Mineral Nutrition of Higher Plants, 2nd Edn. London: Academic Press.

[B36] Martín A. C. del Pozo J. C. Iglesias J. Rubio V. Solano R. de la Peña A.. (2000). Influence of cytokinins on the expression of phosphate starvation-responsive genes in Arabidopsis. Plant J. 24, 559–567. 10.1046/j.1365-313x.2000.00893.x 11123795

[B37] Maurer F. Müller S. Bauer P. (2011). Suppression of Fe deficiency gene expression by jasmonate. Plant Physiol. Bioch. 49, 530–536. 10.1016/j.plaphy.2011.01.025 21334215

[B38] Meena V. S. Maurya B. R. Verma J. P. (2014). Does a rhizospheric microorganism enhance K+ availability in agricultural soils? Microb. Res. 169, 337–347. 10.1016/j.micres.2013.09.003 24315210

[B39] Melotto M. Mecey C. Niu Y. Chung H. S. Katsir L. Yao J.. (2008). A critical role of two positively charged amino acids in the Jas motif of Arabidopsis JAZ proteins in mediating coronatine- and jasmonoyl isoleucine-dependent interactions with the COI1 F-box protein. Plant J. 55, 979–988. 10.1111/j.1365-313X.2008.03566.x 18547396 PMC2653208

[B40] Niu Y. F. Chai R. S. Jin G. L. Wang H. Tang C. X. Zhang Y. S. (2012). Responses of root architecture development to low phosphorus availability: a review. Ann. Bot. 112, 391–408. 10.1093/aob/mcs285 23267006 PMC3698383

[B41] Ogo Y. Itai R. N. Nakanishi H. Inoue H. Kobayashi T. Suzuki M.. (2006). Isolation and characterization of IRO2, a novel iron-regulated bHLH transcription factor in graminaceous plants. Plant J. 51, 366–377. 10.1111/j.1365-313X.2007.03149.x 16887895

[B42] Okada K. Abe H. Arimura G. (2014). Jasmonates induce both defense responses and communication in monocotyledonous and dicotyledonous plants. Plant Cell Physiol. 56, 16–27. 10.1093/pcp/pcu158 25378688

[B43] Pandey B. K. Mehra P. Giri J. (2013). Phosphorus starvation response in plants and opportunities for crop improvement, in Climate Change and Plant Abiotic Stress Tolerance, eds Tuteja N. Gill S. S. (Weinheim: Wiley-VCH Verlag GmbH & Co. KGaA). 10.1002/9783527675265.ch37

[B44] Parida S. K. Mukerji M. Singh A. K. Singh N. K. Mohapatra T. (2012). SNPs in stress-responsive rice genes: validation, genotyping, functional relevance and population structure. BMC Genomics 13:426. 10.1186/1471-2164-13-426 22921105 PMC3562522

[B45] Pauwels L. Barbero G. F. Geerinck J. Tilleman S. Grunewald W. Pérez A. C.. (2010). NINJA connects the co-repressor TOPLESS to jasmonate signalling. Nature 464, 788–791. 10.1038/nature08854 20360743 PMC2849182

[B46] Pauwels L. Goossens A. (2011). The JAZ proteins: a crucial interface in the jasmonate signaling cascade. Plant Cell 23, 3089–3100. 10.1105/tpc.111.089300 21963667 PMC3203442

[B47] Rai V. Sanagala R. Sinilal B. Yadav S. Sarkar A. K. Dantu P. K.. (2015). Iron availability affects phosphate deficiency-mediated responses, and evidences of cross talk with auxin and zinc in arabidopsis. Plant Cell Physiol. 56, 1107–1123. 10.1093/pcp/pcv035 25759329

[B48] Roy S. W. Gilbert W. (2006). The evolution of spliceosomal introns: patterns, puzzles and progress. Nat. Rev. Genet. 7, 211–221. 10.1038/nrg1807 16485020

[B49] Sakakibara H. (2006). Cytokinins: activity, biosynthesis, and translocation. Annu. Rev. Plant Biol. 57, 431–449. 10.1146/annurev.arplant.57.032905.105231 16669769

[B50] Schmelz E. A. Alborn H. T. Engelberth J. Tumlinson J. H. (2003). Nitrogen deficiency increases volicitin-induced volatile emission, jasmonic acid accumulation, and ethylene sensitivity in maize. Plant Physiol. 133, 295–306. 10.1104/pp.103.024174 12970495 PMC196606

[B51] Schulze J. Temple G. Temple S. J. Beschow H. Vance C. P. (2006). Nitrogen fixation by white lupin under phosphorus deficiency. Annu. Bot. 98, 731–740. 10.1093/aob/mcl154 16855013 PMC2806177

[B52] Seo J. S. Joo J. Kim M. J. Kim Y. K. Nahm B. H. Song S. I.. (2011). OsbHLH148, a basic helix-loop-helix protein, interacts with OsJAZ proteins in a jasmonate signaling pathway leading to drought tolerance in rice. Plant J. 65, 907–921. 10.1111/j.1365-313X.2010.04477.x 21332845

[B53] Shankar A. Singh A. Kanwar P. Srivastava A. K. Pandey A. Suprasanna P.. (2013). Gene expression analysis of rice seedling under potassium deprivation reveals major changes in metabolism and signaling components. PLoS ONE 8:e70321. 10.1371/journal.pone.0070321 23922980 PMC3726378

[B54] Sheard L. B. Tan X. Mao H. Withers J. Ben-Nissan G. Hinds T. R.. (2010). Jasmonate perception by inositol-phosphate-potentiated COI1–JAZ co-receptor. Nature 468, 400–405. 10.1038/nature09430 20927106 PMC2988090

[B55] Shyu C. Figueroa P. Depew C. L. Cooke T. F. Sheard L. B. Moreno J. E.. (2012). JAZ8 lacks a canonical degron and has an EAR motif that mediates transcriptional repression of jasmonate responses in Arabidopsis. Plant Cell 24, 536–550. 10.1105/tpc.111.093005 22327740 PMC3315231

[B56] Sobkowiak L. Bielewicz D. Malecka E. M. Jakobsen I. Albrechtsen M. Szweykowska-Kulinska Z.. (2012). The role of the P1BS element containing promoter-driven genes in Pi transport and homeostasis in plants. Front. Plant. Sci. 3:58. 10.3389/fpls.2012.00058 22639653 PMC3355690

[B57] Staswick P. E. Su W. Howell S. H. (1992). Methyl jasmonate inhibition of root growth and induction of a leaf protein are decreased in an Arabidopsis thaliana mutant. Proc. Natl. Acad. Sci. U.S.A. 89, 6837–6840. 10.1073/pnas.89.15.6837 11607311 PMC49599

[B58] Svistoonoff S. Creff A. Reymond A. Sigoillot-Claude C. Ricaud L. Blanchet A.. (2007). Root tip contact with low-phosphate media reprograms plant root architecture. Nat. Genet. 39, 792–796. 10.1038/ng2041 17496893

[B59] Takehisa H. Sato Y. Antonio B. A. Nagamura Y. (2013). Global transcriptome profile of rice root in response to essential macronutrient deficiency. Plant Signal. Behav. 8:e24409. 10.4161/psb.24409 23603969 PMC3907390

[B60] Tamura K. Dudley J. Nei M. Kumar S. (2007). MEGA4: molecular evolutionary genetics analysis (MEGA) software version 4.0. Mol. Biol. and Evol. 24, 1596–1599. 10.1093/molbev/msm092 17488738

[B61] Thines B. Katsir L. Melotto M. Niu Y. Mandaokar A. Liu G.. (2007). JAZ repressor proteins are targets of the SCF (COI1) complex during jasmonate signalling. Nature 448, 661–665. 10.1038/nature05960 17637677

[B62] Ticconi C. A. Abel S. (2004). Short on phosphate: plant surveillance and countermeasures. Trends Plant Sci. 9, 548–555. 10.1016/j.tplants.2004.09.003 15501180

[B63] Troufflard S. Mullen W. Larson T. R. Graham I. A. Crozier A. Amtmann A.. (2010). Potassium deficiency induces the biosynthesis of oxylipins and glucosinolates in Arabidopsis thaliana. BMC Plant Biol. 10:172. 10.1186/1471-2229-10-172 20701801 PMC3017790

[B64] Vanholme B. Grunewald W. Bateman A. Kohchi T. Gheysen G. (2007). The tify family previously known as ZIM. Trends Plant Sci. 12, 239–244. 10.1016/j.tplants.2007.04.004 17499004

[B65] Varshney R. K. Song C. Saxena R. K. Azam S. Yu S. Sharpe A. G.. (2013). Draft genome sequence of chickpea (*Cicer arietinum*) provides a resource for trait improvement. Nat. Biotechnol. 31, 240–246. 10.1038/nbt.2491 23354103

[B66] Victoria Fde. C. Bervald C. M. da Maia L. C. de Sousa R. O. Panaud O. de Oliveira A. C. (2012). Phylogenetic relationships and selective pressure on gene families related to iron homeostasis in land plants. Genome 12, 883–900. 10.1139/gen-2012-0064 23231606

[B67] Vij S. Giri J. Dansana P. K. Kapoor S. Tyagi A. K. (2008). The receptor-like cytoplasmic kinase (OsRLCK) gene family in rice: organization, phylogenetic relationship, and expression during development and stress. Mol. Plant 1, 732–750. 10.1093/mp/ssn047 19825577

[B68] Wasternack C. Hause B. (2013). Jasmonates: biosynthesis, perception, signal transduction and action in plantstress response, growth and development. An update to the 2007 review in Annals of Botany. Ann. bot. 111, 1021–1058. 10.1093/aob/mct067 23558912 PMC3662512

[B69] Wasternack C. Kombrink E. (2010). Jasmonates: structural requirements for lipid-derived signals active in plant stress responses and development. ACS Chem. Biol. 5, 63–77. 10.1021/cb900269u 20025249

[B70] Wu H. Ye H. Yao R. Zhang T. Xiong L. (2015). OsJAZ9 acts as a transcriptional regulator in jasmonate signaling and modulates salt stress tolerance in rice. Plant Sci. 232, 1–12. 10.1016/j.plantsci.2014.12.010 25617318

[B71] Wu K. Zhang L. Zhou C. Yu C. W. Chaikam V. (2008). HDA6 is required for jasmonate response, senescence and flowering in Arabidopsis. J. Exp. Bot. 59, 225–234. 10.1093/jxb/erm300 18212027

[B72] Xie D. X. Feys B. F. James S. Nieto-Rostro M. Turner J. G. (1998). COI1: an Arabidopsis gene required for jasmonate-regulated defense and fertility. Science 280, 1091–1094. 10.1126/science.280.5366.1091 9582125

[B73] Yan J. Zhang C. Gu M. Bai Z. Zhang W. Qi T.. (2009). The Arabidopsis CORONATINE INSENSITIVE1 protein is a jasmonate receptor. Plant Cell 21, 2220–2236. 10.1105/tpc.109.065730 19717617 PMC2751961

[B74] Ye H. Du H. Tang N. Li X. Xiong L. (2009). Identification and expression profiling analysis of TIFY family genes involved in stress and phytohormone responses in rice. Plant Mol. Biol. 71, 291–305. 10.1007/s11103-009-9524-8 19618278

[B75] Yoshida S. Forno D. A. Cock J. H. Gomez K. A. (1976). Laboratory Manual for Physiological Studies of Rice, Vol. 83 Manila: IRRI.

[B76] Zeng L. Zhang Q. Sun R. Kong H. Zhang N. Ma H. (2014). Resolution of deep angiosperm phylogeny using conserved nuclear genes and estimates of early divergence times. Nat. Commun. 5, 4956. 10.1038/ncomms5956 25249442 PMC4200517

[B77] Zhang Y. Tan L. Zhu Z. Yuan L. Xie D. Sun C. (2015). TOND1 confers tolerance to nitrogen deficiency in rice. Plant J. 81, 367–376. 10.1111/tpj.12736 25439309 PMC4329406

[B78] Zhang Z. Li Q. Li Z. Staswick P. E. Wang M. Zhu Y.. (2007). Dual regulation role of GH3.5 in salicylic acid and auxin signaling during Arabidopsis-Pseudomonas syringae interaction. Plant Physiol. 145, 450–464. 10.1104/pp.107.106021 17704230 PMC2048736

[B79] Zheng L. Huang F. Narsai R. Wu J. Giraud E. He F.. (2009). Physiological and transcriptome analysis of iron and phosphorus interaction in rice seedlings. Plant Physiol. 151, 262–274. 10.1104/pp.109.141051 19605549 PMC2735995

[B80] Zhou C. Zhang L. Duan J. Miki B. Wu K. (2005). HISTONE DEACETYLASE19 is involved in jasmonic acid and ethylene signaling of pathogen response in Arabidopsis. Plant Cell 17, 1196–1204. 10.1105/tpc.104.028514 15749761 PMC1087996

