# Supplementary material for: JAZ Repressors: Potential Involvement in Nutrients Deficiency Response in Rice and Chickpea
Source: Front Plant Sci. 2015 Nov 10;6:975. doi: 10.3389/fpls.2015.00975 (PMC4639613; doi:10.3389/fpls.2015.00975)
Supplement: Supplementary file 1 [file Table1.PDF]

Table S1 List of qRT-PCR primers used for Chickpea and Rice

Primer ID	Forward Primer 5'-3'	Reverse Primer 5'-3'
qCaJAZ1a	ATCGTTTGTGATCTGCCGATT	TCTTCTCCAGGAACCGATGAA
qCaJAZ1b	CAACCAGTAGGCTTGCCTCAA	GCTTCTCCAAAAACCGAGTCA
qCaJAZ3a	CAACCAGTAGGCTTGCCTCAA	GCTTCTCCAAAAACCGAGTCA
qCaJAZ3b	CGAAGTTGGCAGCTGGTGAT	GAGGAGGGTGGTGTATTTGCA
qCaJAZ3c	CAGGCTCGCAAAGCATCA	GACCCTTTCCTTGCGCTTCT
qCaJAZ6	CCCTCAGTTTGCCATGCTCTA	TGCACCTTTTCAGCAGGAATT
qCaJAZ8	CCGATCTTCGATGTCCAACA	CGGTGCTGCTGCTCATCTT
qCaJAZ10	GGCCGTGAAATGCATCCA	CCGGTTATTTTCGTCAGGAGAA
qCaJAZ12a	TGTTCCCACAAGGCCTTCTT	CTGCGGCGAAGCGATATTA
qCaJAZ12b	CCCTCAGTTTGCCATGCTCTA	TGCACCTTTTCAGCAGGAATT
qCaCOI1	TGGTTCTCGACTGCGTGATG	CACCGCGTCCCTGTCTTT
qCaMYC1	CCACCCCTGTTTGGATCTCA	CCCGCTCACAGGCAGAAAT
qCaAOS1	CAAATGCCGTTGATGAAATCA	CGGCACAGGTGGTTCAATT
qCaEF1-a	TCCACCACTTGGTCGTTTTG	CTTAATGACACCGACAGCAACAG
CaJAZ6_cds	CACCATGTCAACTTTCCCTAAT	TATGTTAAGGTCAAAGTGTTTGGAA

Rice

Primer Name	Forward Primer 5'-3'	Reverse Primer 5'-3'
qOsJAZ1	CAGCAGGTTGGTGAGCAAAG	TCCATCCCTGATGCTTCCAT
qOsJAZ2	CGAGGAGGAACTTGACCATCTT	CCTGTAGCTCGGTGACGTTGA
qOsJAZ3	CCTCAAGCTCGAAAAGCATCTC	GGCGCAACACTTGACACTCTT
qOsJAZ4	GAGTGCCAATGACAACAAGTCATC	TGATTCGTCGCGGTTGCT
qOsJAZ5	CGAGGCAACTAAAGCAAAAGGA	TGAGTGGCTCTTTGGCAAAATT
qOsJAZ6	TTGATGACTTCCCAGCTGAGAA	GCGCTGTGGAGGAACTCTTG
qOsJAZ7	GTGGGAAGGTGCTCGTGTTC	TGCTAGCCAGCTGCATCAAG
qOsJAZ8	GAAGGCTCAACAGCTGACCAT	TTGGTGGACGGGAAGTTCTC
qOsJAZ9	CGGTCGAGTTGGAAGATGGTT	GGTCAGGCTCGGCGAAAT
qOsJAZ10	CTTCCCCAACTTGCCTCCAA	TGAGTCGTGGGATTTGACGG
qOsJAZ11	CAGGTGAACCCCACTCTCA	TTTCCTTGTTTGATCCAGCTATC
qOsJAZ12	TGCCGATCGCGAGGAA	GGTTCGCTCGTTGTCGTGAT
qOsJAZ13	ACACGTCAGCTTTAATCCCATAATT	GAATAATCGTGCACTGTACAAATGC
qOsJAZ14	TACAGCGATTCGTGGAGAAGAGG	TGTGGTGGTGGTGGTGGTGG
qOsJAZ15	CCATTGACCATCGTCTACGGC	AGCAGCATGGGCGGCTCG
qOsCOI1a	CCAATGCGCTGCAAGACTTT	TCATACTTGGTGAGCTCTCCTACCT
qOsCOI1b	AGGATGCCGTGAACTGGAAT	TCCAGGGCCCCATTTGT
qOsCOI1c	GGTGCAAGGATACAAGGCATCT	GGTCGCACCATTGCCATT
qOsMYC1	ACCAGGGAAGACACCCAA	GGTATCAACATCAGAAGGCG
qOsMYC2	CTAGCGAGGAAACCCAATCG	CCATCCATCCATCCTAACAC
qOsAOS2	GGAGGAAGCTGCTGCAATAC	GGAGGTTGAAGCTTTGGTGA
qOsUBQ5	ACCACTTCGACCGCCACTACT	ACGCCTAAGCCTGCTGGTT

Table S2 Summary of gene/ protein structure of rice JAZ

JAZ Name	Locus ID	RAP ID	Domains/ Motifs	Kome ID	No of Exon	Gene Length Nucleotides	Protein length aa	pI	Protein MW KDa	Alternate Spilicing
OsJAZ1	LOC_Os04g55920	Os04g0653000	TIFY, Jas	AK069326.1<br>AK059441.1	5	654	218	7.4177	23.0182	2
OsJAZ2	LOC_Os07g05830	Os07g0153000	TIFY, Jas	----	2	558	186	10.3353	19.4866	0
OsJAZ3	LOC_Os08g33160	Os08g0428400	TIFY, Jas	AK060047.1<br>AK103459.1	7	1299	433	9.9441	45.3789	1
OsJAZ4	LOC_Os09g23660	Os09g0401300	TIFY, Jas	AK071001.1<br>AK065170.1<br>AK059728.1	7	1251	417	10.2335	43.6124	1
OsJAZ5	LOC_Os04g32480	Os04g0395800	TIFY, Jas	AK107750.1	5	567	189	10.5179	19.4301	0
OsJAZ6	LOC_Os03g28940	Os03g0402800	TIFY, Jas	AK106184.1<br>AK061842.1<br>AK099557.1	5	687	229	9.7149	24.0852	1
OsJAZ7	LOC_Os07g42370	Os07g0615200	TIFY, Jas	AK065604.1<br>AK068566.1	5	735	245	10.0764	25.539	2
OsJAZ8	LOC_Os09g26780	Os09g0439200	TIFY, Jas	AK108738.1	5	699	233	9.1744	24.0531	0
OsJAZ9	LOC_Os03g08310	Os03g0180800	TIFY, Jas	AK070649.1	1	540	180	10.1121	18.3952	0
OsJAZ10	LOC_Os03g08330	Os03g0181100	TIFY, Jas	AK067971.1	1	564	188	8.4558	19.7917	0
OsJAZ11	LOC_Os03g08320	Os03g0180900	TIFY, Jas	AK073589.1	3	630	210	5.4284	22.0349	0
OsJAZ12	LOC_Os10g25290	Os10g0392400	TIFY, Jas	AK061602.1	2	516	172	6.5311	18.2699	0
OsJAZ13	LOC_Os10g25230	Os10g0391400	TIFY, Jas	AK107854.1	1	501	167	9.7372	16.9502	0
OsJAZ14	LOC_Os10g25250	Os10g0392101/<br>Os10g0391801	TIFY (2), Jas	----	3	993	331	10.5871	35.1351	0
OsJAZ15	LOC_Os03g27900	Os03g0396500	TIFY, Jas	-----	2	609	203	10.4645	20.6228	0

Table S3 Summary of JAZ proteins identified in different organisms and different domains present in JAZ proteins

Organism	All JAZs Identified	No of JAZ Proteins with Single TIFY motif	Single Jas Motif	two TIFY motifs	Two Jas Motif	GATA	Zn Finger GATA	Zf DNL
Arabidopsis thaliana	12	11	11	1	1	----	----	----
Physcomitrella patens	4	4	4	----	----	2	2	----
Zea mays	27	27	27	----	----	----	3	1
Brassica rapa	24	24	24	----	----	2	2	----
Chickpea	10	10	10	----	----	----	1	----
Linum	8	8	8	----	----	----	----	----
Manihot esculenta	19	19	19	----	----	----	----	----
Medicago truncatula	9	9	9	----	----	----	----	----
Populus tricocarpa	10	10	10	----	----	----	----	----
Oryza sativa	15	14	15	1	----	----	----	----
Ricinus communis	7	7	7	----	----	----	----	----
Solanum lycopersicum	6	0	0	6	6	----	----	----
Solanum tuberosum	14	14	14	----	----	----	----	----

Table S4: *cis*-elements present in putative promoter sequence of rice *JAZ* genes

Element	JAZ1	JAZ2	JAZ3	JAZ4	JAZ5	JAZ6	JAZ7	JAZ8	JAZ9	JAZ10	JAZ11	JAZ12	JAZ13	JAZ14	JAZ15
-10PEHVPSBD	1	2	1	5	1	6	5	1	0	1	2	2	1	2	2
2SSEEDPROTBANAPA	0	0	0	0	1	1	0	0	0	0	0	1	0	0	0
-300ELEMENT	1	0	1	0	1	0	2	1	2	4	7	0	3	4	2
AACACOREOSGLUB1	0	1	0	0	2	3	1	0	1	1	0	1	0	1	1
ABADESI1	0	0	1	0	0	0	0	0	0	0	0	0	0	0	0
ABRE3OSRAB16	0	0	1	0	0	0	0	0	0	0	0	0	0	0	0
ABREATCONSENSUS	0	0	1	1	0	0	0	0	0	0	0	0	0	0	0
ABREATRD22	0	0	1	0	0	0	0	0	0	0	0	0	0	0	0
ABREAZMRAB28	0	0	0	1	0	0	0	0	0	0	0	0	0	0	0
ABRELATERD1	4	8	10	10	2	4	4	4	7	5	12	5	10	3	7
ABREOSRAB21	0	1	1	5	0	1	0	0	1	0	0	0	1	0	1
ABRERATCAL	1	8	8	10	1	2	2	4	5	2	6	3	3	2	3
ABREZMRAB28	0	0	4	4	0	0	0	0	0	0	0	0	0	0	0
ACGTABOX	6	0	0	2	0	0	0	0	8	0	2	0	6	2	0
ACGTABREMOTIFA2OSEM	1	0	3	4	0	1	2	1	1	1	1	0	2	0	0
ACGTATERD1	18	22	18	18	8	8	10	12	36	10	26	12	30	20	10
ACGTCBOX	0	2	0	0	0	0	0	0	2	0	0	0	0	0	0
ACGTSEED3	0	0	1	0	0	0	0	0	0	0	0	0	0	0	0
ACGTTBOX	0	0	0	0	0	2	0	0	0	2	0	0	0	6	0
AGCBOXNPGLB	0	0	0	0	1	0	0	0	0	0	0	0	1	0	0
AGL2ATCONSENSUS	0	1	0	0	0	0	0	0	0	0	0	0	0	0	0
AMMORESIIUDCRNIA1	0	0	1	0	0	0	0	0	0	0	0	0	0	0	0
AMYBOX1	2	1	1	3	1	1	3	0	2	1	1	2	0	2	2
ANAERO1CONSENSUS	1	2	1	3	1	1	2	1	0	2	2	3	2	2	1
ANAERO2CONSENSUS	3	0	1	0	1	0	4	0	3	1	0	0	1	0	3
ANAERO5CONSENSUS	0	0	0	0	0	0	0	0	0	0	0	0	0	0	1
ARE1	1	0	0	2	1	0	1	0	2	0	0	0	0	0	2
ARFAT	2	1	0	0	1	2	3	2	0	2	4	2	0	1	1
ARR1AT	27	21	28	15	31	32	18	25	20	12	22	20	19	20	16
ASF1MOTIFCAMV	6	3	2	3	0	1	0	3	5	3	5	2	6	3	2
AUXRETGA2GMGH3	1	0	0	0	0	0	0	0	0	0	0	0	0	0	0
BIHD1OS	4	8	3	5	2	5	3	3	3	4	3	3	6	3	3
BOXCPSAS1	0	0	0	0	1	0	0	0	0	0	0	0	1	1	0
BOXIINTPATPB	3	3	2	7	1	6	2	1	2	0	0	1	1	1	0
BOXIIPCCHS	1	0	1	1	0	1	0	0	1	1	0	0	2	0	0
BOXLCOREDCPAL	0	0	1	0	0	2	0	1	1	0	2	0	2	2	0
BP5OSWX	1	0	0	0	0	1	0	1	1	0	1	1	0	0	0
BS1EGCCR	0	0	2	0	0	0	0	0	0	2	1	0	0	2	0
CAATBOX1	28	28	32	23	30	48	23	23	11	19	16	31	21	24	13
CACGTGMOTIF	0	4	8	8	0	2	2	2	0	2	4	2	2	0	4
CACTFTPPCA1	35	26	30	42	14	27	40	31	27	22	26	31	29	29	28
CANBNNAPA	0	0	0	0	1	2	2	1	1	0	0	1	1	0	1
CAREOSREP1	1	1	1	0	1	1	0	1	4	1	1	2	2	2	2
CARGCW8GAT	4	4	2	10	0	8	4	2	0	2	6	4	0	0	4
CATATGGMSAUR	0	12	0	2	0	0	0	0	2	2	0	2	2	2	4
CBFHV	4	2	3	1	7	4	1	4	5	5	0	2	4	3	3
CCA1ATLHCB1	0	0	1	0	0	1	0	1	0	1	0	0	0	1	0
CCAATBOX1	5	7	2	6	4	11	5	7	1	3	3	3	6	2	3

CGACGOSAMY3	3	3	4	9	4	0	1	10	12	5	3	3	9	3	2
CGCGBOXAT	2	12	12	14	10	0	4	8	20	1	14	10	8	8	6
CIACADIANLELHC	0	0	4	1	1	1	1	1	1	3	0	3	2	1	4
CPBCSPOR	2	1	0	2	3	5	0	2	1	0	2	1	0	0	0
CPRFPCCHS	0	0	0	1	0	0	0	0	0	0	0	0	0	0	0
CRTDREHVCBF2	0	0	0	0	2	0	0	0	2	0	0	0	0	0	0
CTRMCAMV35S	0	0	1	1	0	0	2	7	0	0	0	4	0	0	0
CURECORECR	22	14	10	12	6	12	18	8	28	16	18	14	34	12	16
DOFCOREZM	24	28	19	23	30	29	33	18	21	21	27	28	21	16	23
DPBFCOREDCDC3	1	5	5	7	1	2	1	3	1	6	5	4	5	3	5
DRE2COREZMRAB17	0	0	2	1	3	0	1	3	0	1	0	0	0	1	0
DRECRTCOREAT	1	0	2	1	3	1	1	3	0	3	0	0	3	1	0
E2F1OSPCNA	0	0	0	0	2	0	0	0	2	2	0	2	0	2	0
E2FANTRNR	0	0	0	0	1	0	0	0	0	0	0	0	0	0	0
E2FBNTRNR	0	1	0	0	0	1	0	1	1	0	0	0	0	0	0
E2FCONSENSUS	0	2	0	2	2	1	3	2	4	2	2	0	0	1	1
EBOXBNNAPA	12	32	24	26	8	18	16	12	20	22	24	18	16	12	20
EECCRCAH1	7	2	4	2	5	2	5	5	3	0	5	2	3	3	2
ELRECOREPCRP1	2	3	4	1	1	0	2	0	0	0	0	0	2	1	0
EMBP1TAEM	0	0	0	1	0	0	0	0	0	0	0	0	0	0	0
ERELEE4	0	0	0	0	0	1	1	0	1	1	0	2	1	1	0
GADOWNAT	0	0	2	3	0	0	2	1	0	0	1	0	0	0	0
GARE1OSREP1	0	0	0	1	1	0	1	0	2	0	0	0	0	0	2
GARE2OSREP1	1	0	1	0	0	0	0	1	1	0	0	0	0	1	0
GAREAT	2	3	1	2	1	1	2	0	0	2	2	2	0	3	1
GATABOX	9	12	14	19	15	20	15	15	9	10	9	25	14	11	28
GBOXLERBCS	0	0	0	1	0	0	0	0	0	0	0	0	0	0	0
GCBP2ZMGAPC4	0	0	1	0	0	0	0	0	0	0	0	0	0	0	0
GCCCORE	0	0	0	2	6	1	0	1	0	0	2	1	1	5	0
GCN4OSGLUB1	0	1	0	0	0	1	0	0	0	0	0	0	1	0	0
GLMHVCHORD	0	1	0	0	0	0	0	0	0	0	0	0	0	0	0
GT1CONSENSUS	13	20	21	15	34	20	19	14	17	13	25	20	13	9	15
GT1CORE	0	1	0	0	1	0	1	1	0	3	1	1	1	2	0
GT1MOTIFPSRBCS	0	0	1	0	0	1	1	0	0	0	0	0	0	0	0
GTGANTG10	13	13	20	12	11	3	8	13	9	17	16	9	20	11	16
HDZIP2ATATHB2	0	0	1	3	0	0	0	0	0	0	0	0	0	0	0
HEXAMERATH4	2	0	1	1	1	0	0	5	1	1	0	0	1	0	0
HEXAT	1	0	0	0	0	0	0	0	0	0	0	0	1	0	0
HEXMOTIFTAH3H4	1	2	0	0	0	1	0	0	1	0	0	1	2	0	0
IBOX	0	0	10	7	6	12	7	6	5	5	8	5	6	3	10
IBOXCORE	2	6	9	6	6	11	6	5	4	4	6	5	6	3	8
IBOXCORENT	0	0	1	1	0	0	1	1	1	0	2	0	0	0	0
INRNTPSADB	3	3	4	1	6	1	4	6	2	2	1	2	2	0	1
INTRONLOWER	0	1	0	2	0	0	0	1	1	1	0	0	0	1	3
IRO2OS	0	2	5	5	0	0	1	0	0	0	0	0	0	0	1
L1BOXATPDF1	0	0	1	0	0	0	0	0	0	0	0	1	0	1	0
LEAFYATAG	0	0	0	0	0	1	0	0	0	0	0	1	0	0	0
LECPLEACS2	0	1	0	0	1	1	1	1	0	1	0	1	1	1	1
LRENPCABE	1	0	0	0	0	0	0	0	0	0	0	0	0	0	0
LTRE1HVBLT49	0	1	0	0	1	0	1	1	1	1	1	0	0	0	0
LTREATLTI78	0	0	1	1	0	0	0	1	0	0	0	0	0	1	0
LTRECOREATCOR15	2	0	4	3	3	2	6	4	2	8	2	0	8	2	0
MYB1AT	2	2	3	0	3	4	2	2	1	1	5	6	1	4	4
MYB1LEPR	0	0	0	0	1	1	0	0	0	0	0	0	0	0	0
MYB2AT	0	0	0	0	2	0	2	0	0	0	1	2	1	1	0

MYB2CONSENSUSAT	5	1	2	0	2	0	2	1	3	3	2	4	1	6	3
MYBATRD22	0	0	1	0	2	0	0	1	0	0	0	0	0	0	1
MYBCORE	11	3	2	2	5	1	4	2	13	7	6	6	3	8	12
MYBCOREATCYCB1	5	2	0	2	0	0	1	2	6	6	3	3	1	8	4
MYBGAHV	2	1	1	2	0	1	2	0	0	1	1	2	0	2	0
MYBPLANT	0	0	0	0	0	3	0	1	2	0	2	0	0	1	0
MYBPZM	0	2	1	3	1	3	3	2	6	5	4	2	3	4	3
MYBST1	1	2	3	2	3	5	5	4	0	1	2	6	1	3	7
MYCATERD1	2	4	1	4	0	0	1	1	5	1	6	2	4	1	1
MYCATRD22	2	4	1	4	0	0	1	1	5	1	6	2	4	1	1
MYCCONSENSUSAT	12	32	24	26	8	18	16	12	20	22	24	18	16	12	20
NAPINMOTIFBN	1	0	0	0	0	0	0	0	0	0	2	1	0	1	0
NODCON1GM	4	0	3	0	1	5	1	0	0	2	3	2	0	1	5
NODCON2GM	0	2	12	3	9	4	3	6	3	6	4	5	4	3	2
NTBBF1ARROLB	1	2	0	6	1	4	3	2	2	1	2	2	4	1	1
OCTAMERMOTIFTAH3H4	0	0	0	1	0	0	0	0	0	0	0	0	0	0	0
OSE1ROOTNODULE	4	0	3	0	1	5	1	0	0	2	3	2	0	1	5
OSE2ROOTNODULE	4	2	12	3	9	4	3	6	3	6	4	5	4	3	2
P1BS	4	4	0	2	0	2	0	0	0	0	2	0	0	2	0
PALBOXPPC	1	0	0	0	0	0	0	1	0	0	0	0	0	0	0
POLASIG1	3	6	5	10	8	1	5	7	3	3	7	6	9	5	0
POLASIG2	1	3	5	2	9	1	5	2	1	0	0	1	1	0	0
POLASIG3	2	1	6	8	7	3	6	0	2	1	3	5	7	3	0
POLLEN1LELAT52	13	14	12	13	22	4	16	8	13	3	17	17	9	10	11
PREATPRODH	0	2	0	0	1	1	0	0	1	1	1	3	1	0	0
PRECONSCRHSP70A	3	2	2	0	2	2	0	10	4	2	2	2	2	0	1
PROXBBNNAPA	0	0	0	0	1	1	0	0	0	0	0	0	0	0	0
PYRIMIDINEBOXHVEPB1	1	1	1	0	1	1	0	1	2	0	2	0	0	0	0
PYRIMIDINEBOXOSRAMY1A	3	3	0	0	4	0	3	2	1	2	1	4	2	1	2
QELEMENTZMZM13	1	1	1	0	0	1	0	2	0	0	1	1	3	1	1
RAV1AAT	6	7	3	2	7	6	4	1	5	10	2	2	1	4	9
RBCSCONSENSUS	1	2	0	2	1	0	1	1	0	0	1	0	2	0	0
REALPHALGLHCB21	2	0	1	2	3	4	5	1	4	1	6	6	0	2	1
RHERPATEXPA7	3	7	4	3	2	3	3	4	6	5	13	5	6	5	6
ROOTMOTIFTAPOX1	19	31	12	25	10	31	22	9	5	2	18	13	12	16	13
RYREPEATBNNAPA	0	2	3	1	0	3	1	0	6	0	4	3	1	6	3
RYREPEATLEGUMINBOX	0	1	0	0	0	3	0	0	4	0	4	2	1	4	1
RYREPEATVFLEB4	0	0	0	0	0	2	0	0	0	0	0	0	0	0	0
S1FBOXSORPS1L21	0	1	1	0	0	4	1	0	0	2	0	1	0	3	2
SBOXATRBCS	0	0	0	0	1	0	0	0	0	0	0	0	0	0	0
SEBFCONSSTPR10A	0	0	1	1	1	2	3	2	0	2	4	4	1	0	1
SEF1MOTIF	2	3	2	2	1	0	2	0	0	0	2	1	0	0	0
SEF4MOTIFGM7S	4	7	5	5	8	4	0	5	1	4	4	9	2	2	1
SITEIIATCYTC	1	1	3	5	1	1	1	1	2	0	1	1	14	2	0
SITEIOSPCNA	0	0	1	0	0	0	0	0	0	0	0	0	0	0	0
SORLIP1AT	4	2	4	3	1	4	0	3	5	4	3	0	4	7	1
SORLIP2AT	5	2	8	9	2	3	4	4	6	4	4	0	14	4	0
SORLREP3AT	0	1	0	0	0	0	0	0	0	0	0	1	0	1	1
SP8BFIBSP8BIB	0	0	1	1	0	0	0	0	0	0	1	2	0	1	0
SREATMSD	1	1	2	1	1	0	2	1	0	1	0	1	1	0	0
SURE1STPAT21	0	1	1	0	0	0	0	0	0	0	0	0	0	0	0
SURECOREATSULTR11	4	2	1	0	1	4	7	2	5	5	7	4	1	1	3
SV40COREENHAN	0	0	4	0	0	1	1	1	0	3	1	0	0	0	0
T/GBOXATPIN2	1	3	0	0	1	1	0	2	4	1	5	3	2	1	2
TAAAGSTKST1	4	8	5	7	7	6	6	4	3	3	8	4	5	2	4

TATABOX2	3	3	3	1	1	2	3	0	0	2	0	5	1	1	0
TATABOX3	0	2	1	1	0	0	2	1	1	1	3	0	0	0	0
TATABOX4	0	3	2	1	0	3	0	0	0	2	3	1	0	1	0
TATABOX5	0	4	7	8	9	1	9	6	2	4	5	5	9	3	2
TATABOXOSPAL	3	1	2	3	0	1	4	2	0	0	2	1	0	0	0
TATCCAOSAMY	1	1	0	1	1	2	2	2	0	1	0	1	1	0	0
TATCCAYMOTIFOSRAMY3D	0	1	0	0	0	0	1	1	0	1	0	0	1	0	0
TCA1MOTIF	0	2	0	0	0	0	0	0	0	1	0	0	0	0	0
TGACGTVMAMY	1	2	0	0	0	1	0	0	1	0	0	1	2	0	0
TRANSINITMONOCOTS	1	0	0	0	1	0	0	1	2	0	1	0	0	0	1
WBBOXPCWRKY1	1	2	2	1	0	1	3	0	0	1	1	0	1	1	0
WBOXATNPR1	6	8	6	3	1	3	3	0	2	3	4	1	3	5	1
WBOXHVISO1	5	1	6	2	1	1	3	2	0	1	5	2	3	7	2
WBOXNTCHN48	0	0	2	0	0	0	0	1	0	0	0	3	5	0	1
WBOXNTERF3	8	5	12	5	3	2	6	4	0	2	6	5	12	11	3
WRKY71OS	18	16	17	13	5	8	9	10	8	9	14	10	24	17	8
WUSATAG	0	1	0	0	0	2	0	1	0	0	0	0	0	0	0
XYLAT	0	1	0	0	0	0	0	0	0	0	0	1	1	0	0
ZDNAFORMINGATCAB1	0	0	0	0	1	0	1	0	0	0	0	0	0	0	0

Table S5 *cis*-elements present in putative promoter sequence of chickpea *JAZ* genes

Element	CaJAZ1a	CaJAZ1b	CaJAZ3a	CaJAZ3b	CaJAZ3c	CaJAZ6	CaJAZ8	CaJAZ10	CaJAZ12a	CaJAZ12b
-10PEHVPSBD	1	1	1	1	3	2	3	0	2	0
-300CORE	0	0	0	0	3	0	0	0	0	0
-300ELEMENT	2	0	1	2	1	0	2	0	3	1
2SSEEDPROTBANAPA	1	0	0	0	1	0	0	0	0	0
AACACOREOSGLUB1	1	1	1	1	1	0	0	0	1	0
ABRELATERD1	3	2	0	2	1	1	1	0	0	0
ABRERATCAL	1	1	0	0	2	1	1	0	0	0
ACGTABOX	0	0	0	0	0	0	0	0	1	0
ACGTABREMOTIFA2OSEM	0	0	0	1	0	0	0	0	0	0
ACGTATERD1	3	3	0	5	4	1	1	0	4	1
ACGTTBOX	0	0	0	1	0	0	0	0	2	0
AGL2ATCONSENSUS	0	1	0	0	0	0	0	0	0	0
AMYBOX1	2	1	2	1	3	2	1	0	2	1
AMYBOX2	0	0	1	0	0	0	1	0	1	0
ANAERO1CONSENSUS	1	5	4	1	0	2	1	1	1	1
ANAERO2CONSENSUS	0	0	0	1	0	1	0	0	1	0
ANAERO3CONSENSUS	0	0	0	0	0	0	1	0	0	0
ANAERO4CONSENSUS	0	1	0	0	0	0	0	0	0	0
ARFAT	1	0	2	1	3	1	1	0	1	1
ARR1AT	8	5	12	6	8	4	7	2	10	5
ASF1MOTIFCAMV	1	1	0	2	1	0	0	0	1	0
ATHB6COREAT	0	0	0	0	0	1	0	0	0	0
AUXREPSIAA4	0	1	0	0	0	0	0	0	0	0
AUXRETGA1GMGH3	0	0	0	1	0	0	0	0	0	0
AUXRETGA2GMGH3	0	0	0	1	0	0	0	0	0	0
BIHD1OS	2	1	3	3	1	2	4	1	3	1
BOXCPSAS1	0	0	0	0	0	0	1	0	0	0
BOXIINTPATPB	2	1	1	2	4	0	1	0	1	1
BOXIIPCCHS	0	0	0	1	0	0	0	0	0	0
BOXLCOREDCPAL	0	0	1	1	1	0	2	0	0	1
BP5OSWX	0	0	0	0	1	0	0	0	0	0
CAATBOX1	20	13	15	14	15	11	12	2	7	12
CACGCAATGMGH3	0	1	0	0	0	0	0	0	0	0
CACGTGMOTIF	1	2	0	0	1	1	0	0	0	0
CACTFTPPCA1	20	13	11	13	12	17	10	4	12	8
CANBNNAPA	1	1	0	1	1	0	0	0	1	1
CAREOSREP1	0	2	0	1	1	0	0	0	0	0
CARGCW8GAT	1	2	3	4	2	1	3	0	2	3
CARGNCAT	0	0	0	1	0	0	0	0	0	0
CATATGGMSAUR	0	0	3	0	0	0	0	0	0	0
CBFHV	0	1	0	0	1	0	0	0	0	0
CCA1ATLHCB1	0	0	1	0	0	1	0	0	1	0
CCAATBOX1	1	2	1	5	2	1	1	0	5	4
CDA1ATCAB2	1	0	0	0	0	0	0	0	0	0
CELLCYCLESC	0	0	0	0	0	0	0	0	0	1
CEREGLUBOX2PSLEGA	0	1	0	0	0	0	0	0	0	1
CGACGOSAMY3	0	1	1	1	2	0	0	0	0	0
CGCGBOXAT	0	1	0	0	3	0	0	0	0	0

CIACADIANLELHC	0	1	1	0	2	1	3	1	0	1
CPBCSPOR	1	2	0	1	1	2	1	0	1	1
CRTDREHVCBF2	0	0	0	0	1	0	0	0	0	0
CTRMCAMV35S	0	0	0	4	0	0	0	0	0	0
CURECORECR	3	5	2	1	4	3	2	2	4	1
DOFCOREZM	18	6	23	23	13	9	10	4	12	16
DPBFCOREDCDC3	1	1	1	1	3	2	1	0	1	1
DRECRTCOREAT	0	1	0	0	0	0	0	0	0	0
E2FBNTRNR	1	0	0	0	0	0	0	0	0	0
E2FCONSENSUS	1	0	0	0	1	0	0	0	0	0
EBOXBNNAPA	5	10	10	8	9	4	2	0	5	3
EECCRCAH1	3	2	2	1	2	2	1	1	1	2
ELRECOREPCRP1	1	0	0	1	1	0	0	0	1	1
EMHVCHORD	0	0	0	0	1	0	0	0	0	0
ERELEE4	0	2	0	1	1	1	1	0	1	1
EVENINGAT	1	0	0	0	0	0	0	0	0	0
GAGA8HVBKN3	0	0	0	1	0	0	0	0	0	0
GARE1OSREP1	0	0	0	1	3	0	0	0	1	0
GARE2OSREP1	0	1	0	0	1	0	0	0	0	1
GAREAT	2	1	2	1	0	2	1	0	1	1
GATABOX	6	8	8	4	9	6	7	1	11	4
GGTCCCATGMSAUR	0	1	0	0	0	0	0	0	0	0
GMHDLGMVSPB	0	0	0	1	0	0	0	0	0	0
GT1CONSENSUS	18	10	8	10	18	8	8	1	7	11
GT1CORE	1	0	0	1	1	0	1	0	1	1
GT1GMSCAM4	8	3	3	3	6	4	3	0	3	7
GT1MOTIFPSRBCS	1	0	0	1	0	1	0	0	0	0
GTGANTG10	8	4	4	5	8	4	5	1	8	3
HDZIP2ATATHB2	1	0	1	0	0	0	1	0	0	0
HEXAMERATH4	0	0	1	0	1	0	0	0	0	0
HEXAT	0	0	0	1	0	0	0	0	0	0
HEXMOTIFTAH3H4	0	0	0	1	0	0	0	0	1	0
IBOX	0	0	1	0	1	0	0	0	1	0
IBOXCORE	4	2	3	3	4	3	2	0	1	1
IBOXCORENT	0	0	0	0	0	0	0	0	1	0
INRNTPSADB	4	2	4	2	1	1	4	1	3	7
INTRONLOWER	0	0	1	0	0	0	0	0	1	0
INTRONUPPER	0	0	0	1	0	0	0	0	0	1
IRO2OS	0	0	0	0	1	0	0	0	0	0
L1BOXATPDF1	0	0	0	0	1	0	1	0	1	0
LEAFYATAG	0	0	0	1	0	0	0	0	0	0
LECPLEACS2	4	2	1	0	0	2	4	0	1	0
LRENPCABE	0	0	0	1	0	0	0	0	0	0
LTRE1HVBLT49	0	0	0	0	0	0	0	0	0	1
LTRECOREATCOR15	0	1	0	0	1	0	0	0	0	0
MARABOX1	0	1	1	0	1	3	1	0	0	0
MARARS	0	0	1	0	0	1	1	0	0	0
MARTBOX	2	1	0	0	1	2	4	0	1	1
MYB1AT	1	3	4	1	1	2	0	0	2	2
MYB1LEPR	1	0	1	0	0	0	1	0	0	0
MYB2AT	2	1	0	1	0	0	0	1	0	0
MYB2CONSENSUSAT	2	1	0	1	1	1	0	1	0	0
MYBATRD22	0	0	1	1	0	1	0	0	0	1
MYBCORE	2	1	0	2	3	1	0	1	2	1

MYBCOREATCYCB1	1	2	0	0	1	1	0	1	2	1
MYBGAHV	2	1	2	0	0	2	1	0	1	1
MYBPLANT	0	0	1	0	0	0	1	0	1	1
MYBPZM	0	0	2	0	1	0	2	0	0	1
MYBST1	1	0	3	2	2	0	2	0	1	1
MYCATERD1	0	0	1	0	2	1	0	0	0	1
MYCATRD22	0	0	1	0	1	1	0	0	0	1
MYCCONSENSUSAT	5	10	10	8	9	4	2	0	5	3
NAPINMOTIFBN	0	0	0	1	0	1	1	0	1	0
NODCON1GM	2	2	5	2	3	1	1	0	1	1
NODCON2GM	3	2	1	1	1	1	3	1	2	5
NRRBNEXTA	0	0	0	0	0	0	0	0	0	1
NTBBF1ARROLB	1	1	0	0	3	2	1	1	1	1
OSE1ROOTNODULE	2	2	5	2	3	1	1	0	1	1
OSE2ROOTNODULE	3	2	1	1	2	1	3	1	2	5
P1BS	1	0	1	0	0	0	0	0	1	0
POLASIG1	12	9	8	4	6	5	13	0	4	5
POLASIG2	5	2	5	0	0	1	4	0	2	1
POLASIG3	7	6	3	4	1	10	5	0	2	2
POLLEN1LELAT52	9	6	7	17	3	6	1	0	4	5
PREATPRODH	1	1	1	0	1	0	1	0	2	0
PRECONSCRHSP70A	0	1	1	0	2	1	1	0	1	0
PROLAMINBOXOSGLUB1	0	0	1	0	1	0	0	0	0	0
PYRIMIDINEBOXHVEPB1	0	0	0	0	2	0	1	0	0	0
PYRIMIDINEBOXOSRAMY1A	2	1	1	1	2	0	0	0	0	1
QARBNEXTA	0	0	0	0	0	0	1	0	0	0
QELEMENTZMZM13	0	1	1	0	0	0	1	0	1	1
RAV1AAT	7	3	6	3	2	2	3	1	3	4
RAV1BAT	0	1	1	1	0	0	0	0	0	0
RBCSCONSENSUS	0	0	1	0	0	0	0	0	1	0
REALPHALGLHCB21	1	1	3	1	2	2	1	0	1	3
REBETALGLHCB21	0	0	0	0	1	0	1	0	1	0
RHERPATEXPA7	3	1	0	1	3	1	0	0	1	0
ROOTMOTIFTAPOX1	19	13	9	3	6	21	21	1	10	7
RYREPEATBNNAPA	0	0	1	0	0	0	1	0	1	1
RYREPEATGMGY2	0	0	1	0	0	0	1	0	1	1
RYREPEATLEGUMINBOX	0	0	1	0	0	0	1	0	1	1
RYREPEATVFLEB4	0	0	0	0	0	0	0	0	0	1
S1FBOXSORPS1L21	0	1	1	1	1	0	1	0	1	1
SBOXATRBCS	1	0	0	0	0	0	0	0	0	0
SEBFCONSSTPR10A	1	0	1	1	2	1	1	0	1	1
SEF1MOTIF	1	2	1	1	1	6	1	0	1	0
SEF3MOTIFGM	0	1	0	3	2	0	0	0	0	1
SEF4MOTIFGM7S	3	1	7	1	1	2	6	1	2	3
SITEIIATCYTC	0	1	2	0	1	0	0	0	0	0
SORLIP1AT	0	0	1	1	1	0	0	0	1	0
SORLIP2AT	0	0	2	1	1	0	0	0	0	0
SORLIP5AT	0	2	0	0	0	0	1	0	0	0
SORLREP3AT	1	1	1	0	1	0	0	0	0	0
SP8BFIBSP8BIB	1	0	0	1	0	0	1	0	1	0
SPHCOREZMC1	0	0	0	0	0	0	0	0	0	1
SREATMSD	1	0	1	1	0	0	1	0	0	0
SURE1STPAT21	0	0	0	1	0	0	0	0	0	0
SURE2STPAT21	0	0	0	0	0	1	0	0	1	0

SURECOREATSULTR11	2	1	1	2	3	1	2	0	1	1
SV40COREENHAN	0	0	0	1	1	0	0	0	0	0
T/GBOXATPIN2	2	0	0	0	1	0	1	0	0	0
TAAAGSTKST1	6	4	3	3	6	1	2	2	2	3
TATABOX2	2	3	3	2	1	4	2	0	0	2
TATABOX3	1	1	3	1	2	2	2	0	1	1
TATABOX4	3	1	4	1	1	1	4	0	0	1
TATABOX5	5	9	1	1	5	14	12	0	3	2
TATABOXOSPAL	1	2	3	1	0	4	3	0	2	0
TATAPVTRNALEU	1	0	3	1	1	1	1	0	0	1
TATCCACHVAL21	0	0	0	0	1	0	0	0	0	1
TATCCAOSAMY	0	0	1	2	1	0	1	0	1	1
TATCCAYMOTIFOSRAMY3D	0	0	1	0	1	0	1	0	1	1
TBOXATGAPB	0	2	1	1	2	2	1	0	0	2
TGACGTVMAMY	0	0	0	2	0	0	0	0	1	0
TGTCACACMCUCUMISIN	0	1	1	0	0	1	0	0	0	0
TRANSINITDICOTS	0	0	0	0	0	0	0	1	0	0
TRANSINITMONOCOTS	0	0	0	0	0	0	0	1	0	0
UP1ATMSD	0	0	2	0	0	0	0	0	0	0
UPRMOTIFIAT	0	0	0	1	0	0	0	0	0	0
WBBOXPCWRKY1	0	1	0	1	0	0	0	0	0	1
WBOXATNPR1	1	1	1	5	2	1	0	1	1	1
WBOXHVISO1	2	2	1	1	2	0	1	0	2	1
WBOXNTCHN48	0	1	1	1	1	1	1	0	0	0
WBOXNTERF3	3	3	3	2	2	1	2	0	2	1
WRECSAA01	0	0	0	1	0	0	0	0	0	0
WRKY71OS	3	2	6	9	6	2	2	1	5	2
WUSATAg	0	1	2	0	0	0	0	0	0	0
XYLAT	0	0	2	0	0	0	0	0	0	0

Table S6. *JAZ* genes expression patterns under individual nutrient deficiencies

	**Gene expression using qRT-PCR**				
	**N**	**P**	**K**	**Fe**	**Zn**
Gene name	7d/15d	7d/15d	7d/15d	7d/15d	7d/15d
OsJAZ1	nn	nd	ud	dd	dd
OsJAZ2	uu	uu	nd	nn	nd
OsJAZ3	uu	ud	un	dd	dd
OsJAZ4	nu	un	un	dd	nd
OsJAZ5	nu	ud	un	nd	ud
OsJAZ6	uu	uu	uu	nd	nd
OsJAZ7	uu	nd	ud	nn	nd
OsJAZ8	un	ud	ud	nn	dn
OsJAZ9	nd	ud	ud	ud	dd
OsJAZ12	un	un	un	nd	nd
OsJAZ13	nn	dn	nd	nd	ud
OsJAZ14	nn	dn	nd	nd	nd
OsJAZ15	un	nn	un	nu	dn
CaJAZ1a	un	ud	nu	du	nn
CaJAZ1b	nn	nd	nn	nu	nd
CaJAZ3a	nn	nd	du	uu	nd
CaJAZ3b	nn	uu	un	dn	nn
CaJAZ3c	nu	ud	nn	dd	nd
CaJAZ6	du	nd	nn	du	nn
CaJAZ8	dd	ud	nn	dd	ud
CaJAZ10	uu	ud	nd	nn	nd
CaJAZ12a	nu	ud	nu	dn	nd
CaJAZ12b	nu	ud	nn	nn	nd

U, upregulated; D, downregulated; n, non-significant change

Supplementary text 1

>JAZ1
MSSSMECSEFVGSRRFTGKKPSFSQTCSRLSQYLKENGSFGDLSLGMACKPDVNGTLGNSRQPTTTMSLFPCEASNMDSMVQDVK
PTNLFPRQPSFSSSSSSLPKEDVLKMTQTTRSVKPESQTAPLTIFYAGQVIVFNDFSAEKAKEVINLASKGTANSLAKNQTDIRS
NIATIANQVPHPRKTTTQEPIQSSPTPLTELPIARRASLHRFLEKRKDRVTSKAPYQLCDPAKASSNPQTTGNMSWLGLAAEI
>JAZ2
MSSFSAECWDFSGRKPSFSQTCTRLSRYLKEKGSFGDLSLGMTCKPDVNGGSRQPTMMNLFPCEASGMDSSAGQEDIKPKTMFPR
QSSFSSSSSSGTKEDVQMIKETTKSVKPESQSAPLTIFYGGRVMVFDDFSAEKAKEVIDLANKGSAKSFTCFTAEVNNNHSAYSQ
KEIASSPNPVCSPAKTAAQEPIQPNPASLACELPIARRASLHRFLEKRKDRITSKAPYQIDGSAEASSKPTNPAWLSSR
>JAZ3
MERDFLGLGSKNSPITVKEETSESSRDSAPNRGMNWSFSNKVSASSSQFLSFRPTQEDRHRKSGNYHLPHSGSFMPSSVADVYDS
TRKAPYSSVQGVRMFPNSNQHEETNAVSMSMPGFQSHHYAPGGRSFMNNNNNSQPLVGVPIMAPPISILPPPGSIVGTTDIRSSS
KPIGSPAQLTIFYAGSVCVYDDISPEKAKAIMLLAGNGSSMPQVFSPPQTHQQVVHHTRASVDSSAMPPSFMPTISYLSPEAGSS
TNGLGATKATRGLTSTYHNNQANGSNINCPVPVSCSTNVMAPTVALPLARKASLARFLEKRKERVTSVSPYCLDKKSSTDCRRSM
SECISSSLSSAT

>JAZ4
MERDFLGLGSKLSPITVKEETNEDSAPSRGMMDWSFSSKVGSGPQFLSFGTSQQETRVNTVNDHLLSSAAMDQNQRTYFSSLQED
RVFPGSSQQDQTTITVSMSEPNYINSFINHQHLGGSPIMAPPVSVFPAPTTIRSSSKPLPPQLTIFYAGSVLVYQDIAPEKAQAI
MLLAGNGPHAKPVSQPKPQKLVHHSLPTTDPPTMPPSFLPSISYIVSETRSSGSNGVTGLGPTKTKASLASTRNNQTAAFSMAPT
VGLPQTRKASLARFLEKRKERVINVSPYYVDNKSSIDCRTLMSECVSCPPAHHLH
>JAZ5
MSSSNENAKAQAPEKSDFTRRCSLLSRYLKEKGSFGNIDLGLYRKPDSSLALPGKFDPPGKQNAMHKAGHSKGEPSTSSGGKVKD
VADLSESQPGSSQLTIFFGGKVLVYNEFPVDKAKEIMEVAKQAKPVTEINIQTPINDENNNNKSSMVLPDLNEPTDNNHLTKEQQ
QQQEQNQIVERIARRASLHRFFAKRKDRAVARAPYQVNQNAGHHRYPPKPEIVTGQPLEAGQSSQRPPDNAIGQTMAHIKSDGDK
DDIMKIEEGQSSKDLDLRL
>JAZ6
MSTGQAPEKSNFSQRCSLLSRYLKEKGSFGNINMGLARKSDLELAGKFDLKGQQNVIKKVETSETRPFKLIQKFSIGEASTSTED
KAIYIDLSEPAKVAPESGNSQLTIFFGGKVMVFNEFPEDKAKEIMEVAKEANHVAVDSKNSQSHMNLDKSNVVIPDLNEPTSSGN
NEDQETGQQHQVVERIARRASLHRFFAKRKDRAVARAPYQVNQHGSHLPPKPEMVAPSIKSGQSSQHIATPPKPKAHNHMPMEVD
KKEGQSSKNLELKL
>JAZ7
MIIIIKNCDKPLLNFKEMEMQTKCDLELRLLTSSYDSDFHSSLDESSSSEISQPKQESQILTIFYNGHMCVSSDLTHLEANAILS
LASRDVEEKSLSLRSSDGSDPPTIPNNSTRFHYQKASMKRSLHSFLQKRSLRIQATSPYHRYR
>JAZ8
MKLQQNCDLELRLFPTSYDSDSSDTTSVVESTSSGNPQPNEESQRITIFYNGKMCFSSDVTHLQARSIISIASREMKTKSSSNGS
DPPNKSTSFHHNQLPNPKASMKKSLQSFLQKRKIRIQATSPYHSRR
>JAZ9
MERDFLGLSDKQYLSNNVKHEVNDDAVEERGLSTKAAREWGKSKVFATSSFMPSSDFQEAKAFPGAYQWGSVSAANVFRRCQFGG
AFQNATPLLLGGSVPLPTHPSLVPRVASSGSSPQLTIFYGGTISVFNDISPDKAQAIMLCAGNGLKGETGDSKPVREAERMYGKQ
IHNTAATSSSSATHTDNFSRCRDTPVAATNAMSMIESFNAAPRNMIPSVPQARKASLARFLEKRKERLMSAMPYKKMLLDLSTGE
SSGMNYSSTSPT
>JAZ10
MSKATIELDFLGLEKKQTNNAPKPKFQKFLDRRRSFRDIQGAISKIDPEIIKSLLASTGNNSDSSAKSRSVPSTPREDQPQIPIS
PVHASLARSSTELVSGTVPMTIFYNGSVSVFQVSRNKAGEIMKVANEAASKKDESSMETDLSVILPTTLRPKLFGQNLEGDLPIA
RRKSLQRFLEKRKERLVSTSPYYPTSA
>JAZ11
MAEVNGDFPVPSFADGTGSVSAGLDLLVERSIHEARSTEPDASTQLTIIFGGSCRVFNGVPAQKVQEIIRIAFAGKQTKNVTGIN
PALNRALSFSTVADLPIARRRSLQRFLEKRRDRSTKPDGSMILPSQLTIIFGGSFSVFDGIPAEKVQEILHIAAAAKATETINLT
SINPALKRAISFSNASTVACVSTADVPIARRRSLQRFFEKRRHRFVHTKPYSATTSEADKNETSPIVT
>JAZ12
MTKVKDEPRASVEGGCGVADGDGGAAEIGGTGSVEKSINEVRSTEIQTAEPTVPPNQLTIFFGGSVTVFDGLPSEKVQEILRIAA
KAMETKNSTSISPVSSPALNRAPSFSSTSNVASPAAQPFPIQPISFCRSTADLPIARRHSLQRFLEKRRDRLVNKNPYPTSDFKK
TDVPTGNVSIKEEFPTA
>Bra031065
MSSPMESSDFAATRRFSRKPSFSQTCSRLSQYLKENGSFGDLSLGMACKPEVNGISRQPTTTMSLFPCEASNMEPIGQDVKPKNL
FPRQPSFSSSSSSLPKEDILKMTQATSSTRSVKPEPQTAPLTIFYGGQVIVFNDFSAEKAKEVMDLASKGTANTFTGFTSNVNNN
IQSVYTTNLANNQTEMRSNIAPIPNQLPHLMKTTTQNPVQSSSTAMACELPIARRASLHRFLAKRKDRVTSKAPYQLNDPAKASS
KPQTGDNTTSWLGLAAEM
>Bra016520
MMSSSMECSDSAATRRFSRKPSFSLTCSRLSQYLKENGSFGDLSLGMSCKPEVNGISRQPTTTMSLFPCEAAQDVKPKNLFPRQP
SFSSSSSSLPKKEEVLKMTQTTTTRSVRPEPQTAPLTIFYNGEVIVFNDFSAEKAKEVMDLASKGTANSFTGFTSTVNLPKYQTE
VRTNISPTLDQVTHLMKPAAQEPILSSSAAMACELPIARRASLHRFLAKRKDRVTSKAPYQLSDPAKASSKPQTGDNNTTSWLGL
AAQI
>Bra025713
MSSSMECSATRRSSSSGKPSFSLTCSRLSQYLKENGSFGDLSLGMSCKPETNGMSRKPTTTMSLFPCEASNVGSMAAAQDVKPKN
LFPRQPSFSSSSSSIPKEVVPKMTQTTTTRSLKPEPQTAPLTIFYGGQVIVFNDFSAEKAKEVMNLASKGTANTFTGFKSTLNNN
IAPTPNQVPHLMKTASQDPKQTSSAAMACELPIARRASLHRFLAKRKDRVTSKAPYQLSDPAKAFSKPQTGNSTTSWLGLAADM
>Bra008172
MSSSAEYREFSGRKLQKKPSFSQTCSRLSRYLKEKGSFGDLSLGMTCNPDVTGVFAVSRQPTMMNLFPCEEASPTQDVKPTHKVP
RQSSFSSSSSAGAKGEVEKIIETKSVKVESQSAQLTIFYGGQVMVFDDFPAEKVKQVIDLANKGSDYAQNIAKNQKEIASTTPNP
VPSLAKTAAAPELVQTNTSSLACELPIARRASLHRFLEKRKDRITSKAPYQIDGSTEASSRPDTSWLGSQ
>Bra015880
MSSSAECWEFSGERKRLLEKKPSFSQTCSRLSRYLKEKGSFGDLSLGMTCNGGLAVTRQQPTMMNLFPVEDSSDVKQKNDVIPRQ
LSFSSSSSSGAKEEVEKITETKSVKVESQSSVPLTIFYGGQVMVFDDFPAEKAKQVIDLAHKGSAKSFTAELNRNQSAYTQKEIA
STTPVPVPSPVKTTAPEPTQTNKSSLACELPIARRASLHRFLEKRKDRISSKAPYQIDGSTEASSKPNTALLGSQ
>Bra003778
MSSSAECWEFSGPKLPEKPSFSQTCSRLSRYLKEKGSCGDLSFSMTSKPDVNGINSKAAQDVKLQNDMFPCQSSFSSSFGVKEEV
VKITETKPVKPESQSAPLTLFYSGQVMLFDDFPAEKAKQVIDLANKGNANGFTAELNNNQSAYTKNIAKNQKEIASIPRPVPSPA
KSPAQEPIQTNTSSLASELPIARRASLHRFLEKRKDRITSKGPYQKEGSTEA
>Bra016056

MSTGQAPEKSNFNRRCSLLSRYLKEKGSFGDIVIGLARKSDLELAGKNDHRAQQNAIEKANISESRPFKLTQKQLSVGETSISSR
GKAIDVVDLSEPRNVPEPKNSQLTIFFGSKVIVYNEFPEDKAKEIIEAAKEANPVAVDSKKTQNHMNLDINISNKSNVVIPDLNE
PTSSGNNDDHQTKEQHQVVERIARRASLHRFFAKRKDRAVARAPYQVNQNGGHVPPKPQMVGPSVEAGQHSRQPATPSKPQRHND
MSMEVDEEERCSKDLELKL
>Bra016604
MSRNEDGEAPPPEKSNFTRRCSLLSRYLNEKGSFGNIDLGLVRKPGPDLGLPGNSDQQEKQNVMHKANSELKALNVLGEPSSSFG
GKAKATNLSEPSEPISSQLTIFFGGKVLVYNEFPSDKAKEIIQVAKEAKSVTDINIQTQINVQKDHNKSNIVLPDLNEPTDTADV
NQQQQQQNQLVERIARRASLHRFFAKRKDRAVARAPYQVNQNGGGHHYPPKPETVPGQQLEQGQSSQPQRPAQPKPECDKDMLME
VKEEGQCSKDLELRL
>Bra025977
MSRNENAKAQPLEKSNFTRRCSLLSRYLKEKGSLGNINLGLIRKPNPPGKQHKADSETKTLDVFQRVLKGEPSPGKANEDSNLSS
QLTIFFGGHVLVYNEFPTDKAKEILEVAKQAKPVTDINIKTQINVENNDNKSNMVLPDLNEPTNSVDIINQQNQVVERIARRASL
HRFFAKRKDRAVARAPYQVNQNVGQHHYPPKPETAHGRSLKSGQSSKAPEEDVAQTMSQPKPEGDKYMSIETEEEGQCSKDLQLR
L
>Bra030986
MSRKENAKALGPPPEKSSFTRRCSLLSRYLKEKGSFGNINLDLIRKPDSDLGLPGYSCPPGKQNAMQKAVSETKALDVCQRDSKA
EPSPPSGGKAKDTNLSKPASDSGSSQLTIFFGGQVLVYNEFPADKAKEIMEVAKKAKPVTEVNIQTQINVENNNTNNIQTQINVE
NNNNNKSNMVLPDLNEPTDSMDINPQQQQENQVVERIARRASLHRFFAKRKDRAVARAPYQVNQNGGHHHYPPKPETAHGQPLES
GQSSKRPENAVAQTMSHPKPVGDKNTSIKIEEEGQCSKDLELRL
>Bra008033
MSNGKAPEKSSFSRRCSLFSRYLKEKGNLGNIDIGLSRNLDLELVRKSDLSGQQNEIKKADISETRPFALSQKVLVGEASTSSGG
KPRFVDLSEPASLVVPEPGNSQLTIFFRGKVMVYDEFPEDKAKEIMAAAREAHHVAVDSKNTQNLDMNMSNKTNVVIPDLNEPTS
SGTNNDDHQTGQQHQVVERIARRASLHRFFAKRKDRAVARAPYQVNQSGGHLPPKPQKVGPSVESGQPSRQPETPSKPKRHNDAS
MEVDGEEGRCSKDLELKL
>Bra020135
MVKVEVEPRAPVEGGCGDILGCGVVDGDGDEENHVVEIAGNGTVNGTIDGSVAGEGGFSAEEPVHEARSTDAPSSDVPDPSTILP
NQLTIFFGGKVCVFDGIPAEKIQEIIRIAAAATAKSIETKNSTSVKPVLSPALNRAPSFSSTSTGASPAAPSLPVNPIPFCRSPA
DLPIARRHSLQRFLEKRRDRLVNKNPYPASDMKKTDVPTDIASIKEESPIA
>Bra022254
MERDFLGLGSKNSPITVKEETSESSRDSAPNRGMNWSFSKKGSAASSQFLSFRPSQDDRHRKPGNYHLPHSGSFMPSSVADVYDS
NRNTPYSSVQGARMFPNSHQQESITVSMARPGLQSHYPPGGKSFMSNGINSQPFVGVPIMAPPISVLPAPGSIVGTTDIRSSSK
PLGSPAQLTVFYAGSVCVYDDISPDKAKAIMLLAGNGSSMPQAFSPPQTHQQVVHHARASVDSSAMPPSFMPTVSYLSPEAGSST
NVLGARGFASTYHNNQTNASTVKPQTVALPQARKASLARFLEKRKERVTSVSPYCLDKKSPTDCRTPISECISSSFSSAT
>Bra002338
MVKVEEEARASVEGGCGVGGEEIGGNGTVHGSIAGSVAGEGAEKPIHGAGCIEVPSSEPDAPTTRPNQLTIFFGGKVRVFDGIPA
DKIQEIIRIAAAAAKSIETKNSANTSPVASPALNRAPSLSSTSNAAASPAAQSFPVHPISFCRSAADLPIARRHSLQRFLEKRRD
RLVSKNPYPASDKKTDVPRDDASIKEEYPTA
>Bra008846
MSKVTTELDFFGLEKKQTNNGPKPKFKKLLDRGRSFREIQGAISKMDPEIIKSLLATGANPSDTSTRSLSVPSTPKEDHQRILIS
PVHAPLTRPSTELVSGTVPMTIFYNGTVSVFQVSPNKAEDIMKVAMETSPKKDKSMEKDLSVIPPTTLRTKLFGQNIDGDLPIAR
RKSLQRFLEKRKERVVSTSPYFPTSA
>Bra032362
MEKNCDLELRLFPTSSCITDSDNSVVESRSSGNSLPKEEETQRLTIFYNGKMCVYSNVTHHQAKSIISMAGREMEEKVSSNVSDP
RNRSTRLNNYHQQLPNPKASMKRSLQSFLQKRQIRLQAASPYHQHSRR
>Bra003947
MERDFLGFSDKQYLNNVDDDRVGERGLCSCLVTCSSTKAARQWGPESAASIHRRSQYSGAFQNANPQLTIFYAGTVCVFNDISPD
KAQAIMLCAGNGLKIDNGESRLKKPLIETERVYGKQFHNAATAAASSSSATYCDNFSRCGDRPVGATNAMSMIESFNVDPGYMMP
SVPQARKASLARFLEKRRERLMNAMPYKKMLLDLLTRESYGMNYSSASHT
>Bra010794
MEKNCDLELRLFPTSSYDESDTSVVESRSSGNSLPKEEESQRITIFYNGKMCVSSNVTHLQAKSIISIASREMEERSSSNGSDPR
NRLTRLHHHQLPNPKASMKRSLQSFLQKRRIRIQAASPYHQHSRR
>Bra006190
MSRVTTELDFFGLEKKQTNNVPKPKFKKFLDRRRSFRDIQGAISKIHPEIIKSLLASGANHADSSTISPSVPSTPKADYPQIPIS
PVQAPLTMQTEHSSGTVPMTIFYNGTVSVYQVSPNQADDILKVVMETAPKKDKSIVKDHLVIPPTTLRTKLFGKNLEGDLPIQRT
RSLQRFLEKRKERLVSISPYFPTSG
>Bra005407
MEMQSNCDLELRLLPPRDPRSSEKLQPKQEPQKMVIFYNGRVFVSSDLTHLQAKAILSLASGDMKEKSLSLESSDGSDPSTVPNI
LTRKASMKRSLRSFLQKRNVRIQASCPYHHS
>Bra021281
MERDFLGLGSKNSPITVKEETSESSRDSAPSRGMKWSFPNKASATSSPQFLTFRPSQENRHRNLGNYHLPHSGSFMPSSVADVYD
SSYRSTPYSSVQGVRMFPSSKQHEEAISVSMSRPSLQSHYASGGTSFINNSVNSQPLVGVPIMAPPVSVLPPPGSIVGTTDIRCS
SKPSGSSPAQLTIFYAGSVCVYNDISPEKAKAIMLLAGNGSPMPQVFSPPQTHQQVVHHARASVDSSAMPPSFMPTVSYLSPEAG
SSSNGFGAAKAARDFTTTYLSNQTNASNINSSVAASCSANVPQTVALPQARKASIARFLEKRKERVTSLSPYCLDKKSSTDCRTP
MSECISSSLSSAT
>Bra019227

MFPQLEINPYQIGSTLGEDHVSASASRIPYIDEIANHPESIYAASGLIPDGSQLLDSPAPEGANQLTVSYRGQVYVFDSVGPEKV
DAVLLLLGGSTAAPQGMEIAQQNHHMPVVEHQSRCSHPHRAQSLDRFRKKRSARCFANQVRYGVRQEVALRMPRNKGQFSSATTA
DGAYNSGTDQDNAHDDGRPELSCTHCGVSSTCTPMMRRGPSGPRTLCNACGLFWVSRGTLRDLSKKTEDNQVAMIEPGELGSDAD
ANNSNY
>Bra014292
MDDGELHGSNGRMHIGETQDPLHHVHYGHHALQHIHNGSGMVDDHADTGGGGGMSDGVDTDVPSHPGIITDNRGEVVDRGSEQGD
QLTLSFQGQVYVFDSVLPEKVQAVLLLLGGREVPQAPPAGLGASHQNNRGLPGTPQRCSMPQRLASLVRFREKRKERNFDKKIRY
TVRKEVALRMQRNKGQFTSAKSNNGEAAADGSSWESSQTWAIEGSEAQNQEISCRHCGIGEKSTPMMRRGPEGPRTLCNACGLMW
ANKAS
>Bra023900
MRSFLRFNLDTSFLSIVVFGEFIVDDKRGDEVKKFWLRNIFVLLCSRFTKLQEEIVRPRSGDAAPFVVKPTLKMSETMDDDLHGN
NGGMHNGGGVQDPNHSMHVQYDHHGMMDEQHADDVMNEGLEADIPSHLGNTSDHRGEVVDRGSENGDQLTLSFQGQVYVFDRVLP
EKVQAVLLLLGGREVPNTVPTNVGSPHQNNRGLSGTPQRFSARHRQASLLRFREKRKGRNFDKTIRYTVRKEVALRMQRKKGQFT
SAKSSNEDSASNGSDWGSGQSWALEGTEAQKPEALCRHCGTSEKSTPMMRRGPEGPRTLCNACGLMWANKGALRDLSKAPPPPTA
QNLPANKIDVSEKHQFFLLYYIA
>Ca_01810.1
MDGIRGGDSRMQISDGQHPVHVPYEHHGLHHMSNGNGMDDDHNNGRDSNCGGSESVEGDI
PSNHGNLHDNHNLMMDQGNDIGDQLTLSFQGQVYVFDSVSPEKVQSVLLLLGGREMHPTL
PSVPISPDENNRGFIGTPQKFSVPQRLASLNRFREKRKERNFDKKIRYTVRKEVALRMQR
NKGQFTSSKSNHDESASAAMNGGTNEGLIADNNGSQQHDIVCRHCGISEKCTPMMRRGPE
GPRTLCNACGLMWANKGALRDLSRAATLPVAHNSPLNKNENKNSETNQIVLRDAAESSS
>Ca_06739.1
MERDFMGLNSKEPLSFVKEEINDVGCKNSSFMKGSAAQWPFYNKVSNPTHLMSFKVSQDD
NKTKILSDPITSAAGFMSVLSPTAFDSSHKRSAAEHQKCLNHDGQGGFHFSLTPYPIQHD
VKMFSVSNNHFVTTGQNMNGYNVTLPLLGGIPVTLPQSSLPTVGAVAGITESCNVKPTGP
SSRLTIFYAGTVNVFDDISAEKAQAIMLLAGNGLSMASNIAQTKVAAPGSKLAAGDGVPV
SQHANTPPSSGHPSPISISSHAGTQSGCGSTSNDEFLAVKTTGVLATLVSNLELPKVVNA
ATMLASAVPQARKASLARFLEKRKERVMNAAPYNFNKKSEECAAA
>Ca_07919.1
MSTFPNTVSDSQRSGKAPEKFKFSQTCSLLSQFLKEKRISGDSTPGLFGKIKPKASTKDL
LGNKQKLRWRSTNSETPQLTIFYAGKMLVFDAFRPEKATEIMELATKLASENSSREENPT
SAPITSEKLKDSKVPQPKTALETPRENQVIGSDMRYPRRASLLKFLEKRKERVISRGPYQ
INNHKIEGSSSGGEPKEQCSKHFDLNI
>Ca_08149.1
MSSSSENSGFSGHKPAKLPEKSNFSHTCNLLSQYIKENGSFGGLTLGKPCTVETNGSPET
SCNSGTTMELFPTNMTPQTQNLKTLNLLSPNDVPALENSSVFKEPKTAQLTMFYGGKIIV
LDEFPANKVEELISFARTTKWSTYASYNQTQPSVIPNLFPQAPSRLIVCEQPIARKASLH
RFLEKRKDRIAAKAPYQKSNPISAPVKPVKSIPWLGLGATSTQV
>Ca_08181.1
MASADSINNNVGQRSDNINSFRHQDSVNNFSVNWPMAVTGQNAVTPQFAMLYNGSMCVYD
GIPAEKVHEIMMMASANAKSSEMKSGIPFTSLISTSPSSPQGGTSNNLASLQSVSFPVEK
SSICRIQEFPITRRQSLQMFLEKRKIRLGSKAPYTSSTSKKVNNVENNFSSVLRLLEGK
>Ca_08733.1
MEREFLGLNSKNIAWFNMKGDASNKPKDPVRSSGMQWSFSNKASTVPQFLSFKNNTHEDR
SRNTTMDPLASSGYMTISTKDAFDSNQKSFLAVTQESLSIGKQVTNKLGMTIYPMQCSDH
AQSICNQETRIFSVSNQSNQMCSVLQSNHATNGINMVNSVIKSQTLGSKSSATPLSVLPS
IGSLIGSTDLRNRNCSKSNGTPTQLTIFYGGSICVYDDISPQKAQAIMLLAGNGPKLQPE
ISVPSEKDGFIISQSYPSPLPHANSQPRGGSSSNNEHSIIRPIVPSIVPINHLESSIVAT
SLRSTPTKVIQPVGLPQARKASLTRFLEKRKERAMSTSPYYMCKKSSECNTLGSDSTSFS
IDFSASSPQLATNLPLRTTCMEV
>Ca_17203.1
MRQVHVWIGGFTQVSAVKWPFWNKVSAHSYLMPFNVSEEEKRATAGGLQKSFKHDGQGGI
HYSLNPYPVQHNVNYANRPHDVKMFSVSAGSPFLKNHFATVGQNMNGANVMQPLFGGLLP
VTAPHSVLPIVGTVTSSAEPCVKPPAPAPQLTIFYGGTVYVFNDITPETAQAIMLLAGNG
VSASLDGAQPEVQAPISKFASGDDVPMSPPADIPPCSGISSPLSVSSHTGPPFGSGASSS
DEFLDAKPSKGPTPTISVSKVETPKIVNATTMFPSAIPQARKASLARFLEKRKERVMSAA
PYNLNKKSEDAPTPNSMVANVSATTGTKTPSAKQG
>Ca_18081.1
MSTSSEHSEISGQKPARSPEKSTFSQTCSLLSQYIKEKGSFKDLSLGMTCNTDPIGSPET
SSHSATTMNFFPNKENNLTPKNLTTMDLLTPQAALNNSNAIKGPKAAQLTMFYGGQVIVF
DDFPADRAHELMSFASKGISQSHNNSVFTYTQSQPSFPINLVRTSADSTTPIVPSVNIVA
NTGAGSVLEHPQVPSRPIVCDLPIARKASLHRFLEKRKDRIAAKAPYQRTNTMEHVNKAT
ESMSWLGLGAKPTQI
>Ca_18578.1
MDGVTVKVEPEQFMVLESSSIAADCVDGVSSNMGDVSMMNLSGNKSMPSSGLNAVIPNTS

QLTIFYNGSICIYDGIPAEKVHEIMLIAAASAKSTEMKKIGKQSPILSTVPTRPSFPHGT
IDNIASPQALCFPAKNSSICKLQARRHSLQRFLEKRRDRLGSKAPYPSSPSTKVADNLEN
NLCADNSPDSVSLKRPDEKFQPTISAS
>Ca_19748.1
MRKKLNLELCLFPSSNSDLRCPTKEANESEVKDEQQHRPLTIVYDGKVCVCDATEIQAKS
ILMLANKEMEERVRTPRSEPSSPTLLVSSHSHYHNHLYTPAGTSLSMKKSLQRFLQKRKN
RIQEASPYHLKLNDQN
>Lus10011929
MESETGQSAEEQVANNKVEVKKEEAAENEGEETGIIGGGADQKSGSEPTAALPSSNSSSSSRDEALAKSGIVDTSVPAEPEQLTI
FYGGSVIVLDGIPADKAREIMAIAAAAAKAVKSTDIKKIETATPTPASPATAPAVTGTGTGTPLLSRSATMQSTTATPTGLASEQ
YAAHKNSLCKMQAELPIARRHSLQRFFEKRRDRMMTKSPYSIPSSPSAAKLEDNQKMAAAAAAAATTTPNHIVLLV
>Lus10027639
MESETGQSPEEQVVNNKVEVKKEESAELEGEETEIVGGGADHKSGSEPTAALPSSNSSSSSRNEALAKSGIVDTSVPAEPEQLTI
FYGGSVIVLDGIPADKAREIMAIAAAAAKAVKSTDIKKIETATPTPAPASPAAAPGGTGTGTPLLSRSATMQSTTATPTGLASEQ
YAAHKNSLCKMQAELPIARRHSLQRFFEKRRDRMMTKSPYSVPSPSSAKLEDNQKMAAAAASTTPNHMV
>Lus10039911
MSSSPEIVEFTGKKLTAEKPSFSQKCSMLSQYLKEKGNFGDLTLGIMTSPADAHPSSGNDMLRHSPAMTLFPLSNGHANHQMGCF
LPPPPSSHQQPMNLFPQKAGFYDSPTLLEPSANKPTSATSQLTIFYGGQVIVFNDFPADKAEEVMLMASRGSAKSQTAAVETNPT
FAPSVTAKTPIVSSSTTSVATPVIATSGIVTSFANNVIQDCKQVQQPHKPIACDLPIARRASLHRFLEKRKDRITAKAPYQTSNG
SASSQGTHTPESKLASEESNNSWLGLVSQR
>Lus10027648
MSSSPEIVEFAGKKLTADKPSFSQKCTMLSQYLKEKGNFGDLTLGIMTSPADAHPASGNDMLRHSPAMTLFPLSNVHANHQMSCF
LPPPPSSHQQPMNLFPQKAGFYDSPKLLEPSANKPTSATSQLTIFYGGQVIVFNDFPADKAEEVMLMASRGSAKSQTAAVETNPA
FAPSITAKTPIVSSSTSSVATPVIPTSGIVTSFANNMIQDCKQVQQPHKPIACDLPIARRASLHRFLEKRKDRITARAPYQTSNG
SASSQGTHTPESKLGSAESNNSWLGLVSQSPPSANC
>Lus10031333
MERDFMGLGSSIHLTTTTVKAEAGDSTSAAAGKDSGMLLLFFFDIDDDDCESRGCRFLLRRRRQGFRDMLVSNRASMRIQVRNGS
MINNMRNSGAHQWSFSNKVSAVPQFLSFQTAPDQENPRRSIFDPVASSSSFLSISNADAFDSANRRGSSSIMQKIWNFENKDESS
RRARPVIDAFPVGCSKDIRTFPLSNRQQHQPFPVSMSTPSLQSHFTSTVHSGYYVNSMATQQLGGVPIAAPVIGTTELRNSPNFS
APSAQLTIFYAGSVCVYDDVSPEQAQAIMSMARTDSSAPQTTTFTPTTLHTQPAIHNHLATDDHIVRNRPSQTPPGSSTASPVSV
SSSTNNESRTARSPVASTSIHSSSIPSSSSRPASAAPIPASVGLPQVRIPQARKASLARFLEKRKERVTSTASPYYDISKKSAEG
HVCV
>Lus10001803
MSRGGATVELDLFRGINNTCTHVVDGGRPPSESRFHRLLRRQRSHRDIQLGAISKIDPQVLKSVFNNYASNQSHTTTPPATLPLY
PTAAPCENDTAPLTIFYNGSVVVFDLPRDKAENILKLAENNGLPSSPACTPAAVPSSLADHVDSVTGAELPIARRKSLQRFLEKR
KESRKEMTVIRYPPQQWQRVRSVVYGGGGKLAQIISTIHTRQNPH
>Lus10013166
MEKPGSSFAQTCNLLSQFLKSGKANLGDINLAAKLGHHQSFKSGGAPTTTLNLLPSIGNDDGNSKKARDLMDSLPQFVAAGSPPA
METKGSALPTPAQMTIFYGGRVIVFDDFPAEKAKEVMALASGNGAIIKNNNSNNNNSSDERNVNSSASAGSGAASSSSSPEASQI
ERLPLRPIPSDLPIARKASLHRFLEKRKDRVTSRGPYHSPSSAAAAAKPAGGAGSNNSNNKWLDRQMSQSSKQLELSL
>Lus10008129
MAAAGNRYSTERHSSGSAGSSFAQTCNLLSQFLKSGKANLGDISLAAKLDHHQSFKSGGAPTATLNLLPSIGNDDGNSKKARDLM
DSLPRFVAAGSPAMEIKGSVLPAPAQMTIFYGGRVIVFDDFPAEKAKEVMALASGKGATIINNNNNNSNSADERNVNSSASAGSG
AASSSSSPEASHNNNNNGGQIERLPFRPPHQPIPSDLPIARRASLHRFLEKRKDRVTSRGPYHFPSSPAAAKPVGGAGSSNNKNN
PWLDRQMSQSSKQLELSL
>GRMZM2G126507_P01
MAKSGASFPESSWMERDFLAAIGKEQQHPHKEEAGAEESAYFGGAGAAAAAPAMDWSFASKPGAAPALMSFRSASFPQFSSFDGA
KNPAPRILTHQRSFGPDSTHYAAAHRTQHALNGARVTPVSSPFNQNSPMFRVQSSPSLPNGTAFKQPPFAINNNAAASSTVGFYG
TRDVVRPKTAQLTIFYAGSVNVFDNVSAEKAQELMLLASRGSLPSSAPVARKPEAPILAPAKVTAPEVLH
ATQMLFQKPQHVSPPSSAISKPIPGILQAASLPRSASSSNLDSPFPKSSVPFPVSPVSQAPRAQPATIAATTAAAIMPRAVPQAR
KASLARFLEKRKERVTTAAPYPSAKSPMESSDTFGSGSANDKSSCTDIALSSNHEESLCLGQPRNISFIQESPSTKLQI
>GRMZM2G065896_P01
MAAEPAAADHDLRPPLADGAAAAGVGAASLAAAAGAAEALMSATSEQLTLVYQGDVYVFDPVPPQKVQAVLLVLGGYEVPPGLVN
MAVSSANDEKNTTVAARRVASLMRFREKRKERCFDKRIRYSVRKEVAQKMKRRKGQFAGRSDFGDGACSSAACGSPANGEDDHFR
ETHCQNCGISSRLTPAMRRGPAGPRSLCNACGLMWANKGTLRSPLNAPKMTQQLLANPCNMVDTDDKNSNVLPVEHNQATPKTDS
MMPKEEQKLDIRLPTEEDTKAVS
>GRMZM2G145458_P01
MAAAGSVQGHGARFAAACGVLSRYVKAAAVATTTTVELRPAGTVGVLPLMPGADLSTQEEREAGAGPGPSPSPSAQLTISYGGRV
VVLDDVPADKAAEVVRLAAAQGAPRALRAPPTKADDLPMARKVSLQQFMERRKGRVATRGSPYRRPASLPDHLTLTL
>GRMZM2G036351_P01
MAMAAADGKSRRFALACGVLSQYVKAEQQQQQMAAPRAPATTTLSLMPGADVGADEQQEAAAAGAEEMPGPAPAAAPPLTIFYG
GRVVVFEDFPAEKAAEVMRLAAGDDLPIARKASLQRFLAKRKDRLVERAPYARPSSPAEAVKPASGSASASWGLRSTEADRLTI
AL
>GRMZM2G054689_P01

MPGPAPAVAPPLTIFYGGMVVVFEDFPAEKAAEVMCLAAGDDLPIARKASLQRFLAKRKDRLVERAPYARPSSPAEEPEKKTVKP
ASALASWLGLDSTEADCLTITLFCHRLVLRFCPPPTVRGYGPGGVRRLGERLDDGVRNGGATDVFTKSPRWHMRVVLTLPWSLLT
KSPRWHMRVAFACSVCGQCTTRAINPHAYTNETVFVQGVQKHEEASKVKDDSVLRYQLILLIFLNISGKFYTCSCQLILVYLT
>GRMZM2G063632_P01
MVDQYVYDSVPPEKAQAIMLIAAAAAAAAATKGSAATAFNPPMVHTATVAPAAVFSPVLTRSPSLQSTSVAAGQAQVVADPSSIS
KLHADLPIARRHSLQRFLEKRRDRVVRKAPYSPAKSFDGMESAGMEMTADGKQGARPSILKGWGAAKSEV
>GRMZM2G338829_P01
MPGADVGADEQQEPAAARAEEMPEPAPAPAAAPPLTIFYGGTVVVFEDFPAEKTAEVMRPAAGDDLPIARKASLPWFLTKRKDRL
VERAPYARPSSPAKEPEKKTCSVEM
>GRMZM2G173596_P01
MAAAAPSGGTGKNTAATATTPSRFAAACGALSQYVKAAEAERTRARPPVRRPLPLMPGADVDQDEPETAAQLIIVYGGRALVLDD
VAADKAADLLRLAAAAAARGGTEQPLCSLADLPVARKASLQRFMEKRKDRVAARAEPYRRRRPVGDRRNDLALEL
>GRMZM2G101769_P01
MAASARPGERATSFAVACSLLSRFVRQNGAAPAQLGLGIKGEVEQQRTPATINLLPGADGEETERRKETMELFPQSAGFGVKDAA
AAPREQENKEKPKQLTIFYGGKVLVFDDFPADKAKDLMQLASKGGPVVQNVVLPQPSAPAAAVTDKAVPVPVISLPAAQADAKKP
TRTNASDMPIMRKASLHRFLEKRKDRLNANAPYQTSPSDAAPVKKEPESQAWLGLGPNAVKSNLNLS
>GRMZM2G005954_P01
MAGSAPATAMDKTSFATTCSLLSQYVKEKKGGLLQGLGALAMAPAAGEGAFRPPTTMNLLSALDDAPAEERSEKATAGEPKHQDK
CTGGNPREEAAGEEEEEAQQLTIFYGGRVVVFDMFPSAKVEDLLQIMSPGGDGVDRAGGATVPTRSLHRPSHDSLSDLPIARRNS
LHRFLEKRKDRITAKAPYQQVNSSVVGVEASKQAAGAGVEKPWLGLGQEATTAKLEM
>GRMZM2G114681_P01
MDWSFASKPCAAPALMSFRSAAREEPSFPQFSALDGTKNTAPRMLTHQRSFGPDSTQYAALHRAQNGARVVPVSSPFSQSNPMFR
VQSSPSLPNSTAFKQPPFAISNAVASSTVGSYGGTRDAVRPRTAQLTIFYAGSVNVFNNVSAEKAQELMFLASRGSSAPVACKPE
APPTLAPAKVTAPEVLLPAKQMLFQKPQHLSPPPSSVPGILQSAALPRSASSSSNLDSPAPKSSVPLAVPPVSQAPPATLIATTT
AAAIMPRAVPQARKASLARFLEKRKERVTTAAPYPSAKSPLESSDTFGSGSASANANDKSSCTDIALSSNHEESLCLGGQPRSII
SFSEESPSTKLQI
>GRMZM2G445634_P01
MAMAAAAEGKSRRFALACGVLSQYVKAEQQMAAAGAPAPRAPATTLSLMPGADVGAEQEQAAARGQETAGSASTAAPLTIFYGGR
VVVFDDFPAEKAAEVMRLAAGAERPAAPAPAPAPRDDLPIARKASLQRFLAKRKDRLVERAPYARPSPSEEAEKAKPASSWLGLG
GTDAERLNIAL
>GRMZM2G327263_P01
MESKEENVALTVIQRDNTLSIDESLLMSLSHNKPKTSVTTTIVPKDYICTQGDLALIDWIKEIPCEPRVEVVLIDDAFVERKWME
CLFEPDAYLGDEVIDCYINLIKAQEHLKCRSGGRVHIENAFQFNFLKRDGDVDTKTDELYPSKDMAQISSAERRVLLYLDHDMVF
IPINIREMHWYLAVINARNMEIQVLDSLGTSSGRNDLIDTIKGLQRQIDMVSQRKELKDHRWPDLRVASWPLREIEMEYAKQTDS
SSCGLFLLNYIEYWTGDELSDNFTQDDMSHFRKKLAAILLSSDINKRKGCPLYKYDKEVDAGCSSDVQILDSPTNPKKRKLLCVS
EKSEVLMEDDDGPITQADLEKWFVHDWDKRTPIKISTDECTNEFLLSGLSTKDMPVTKADLIDVLCDYIMTIQDDTALEMTWVRS
FNPFKIEISVKDLQNVLRVNLDMTLKCFDMAVRLLAIKESHMSKDEMIKDKKHYMDTRFWRMVGFGKLPKYHQDPTAEELANTLD
CWPSLNYYITGCKYVLMPWKFNGCYALFVIDHGKKHVTFIDFTPTQDWCKHMPYKRFAEAIIMASKKYKIAYNKKRSGWADDIFK
WEHTIRSGLPMDLKGVNTSYFVLQAMVMWGSGRRMEFNRWTSLDLAKMSREIRGSPEILFAREVARACRMGNFISFFRLARKATY
LEACLMHAHFAKEWLEANKDYRAIVDGANIALYQPNFAEGGFSLTQLMENPSNRHLIETWRTNGALYTSPSGSNDDWSAMLPMSN
PSANPTQLTIFYGGSVCVYDSVPPEKAQAIMLIAAAAAAAAATKGSAATAFNPPMSTSVAAGQAQVVADPSSISKLQADLPIARR
HSLHRFLEKRRDRVVSKAPYSPAKSFDGMESAGMEMTVDGKQGARPSILKAQAPVAAVVAAISSIRFSSSLPALVPPPPPLHDEN
PFAALLASEPPPPEPLRQVLATGDVHSALRGLPGLARQLFRWAETTPCGFPRSASAFAAVLIPLARANHIRAAYPVSLRALHLDL
LLPLVSLLSAPLSTAPRSLLSLLLRLSTKYSKECKARDATLDTCSTLCLSAFREMASHGVAPDVKDCNRVLRVLRDAARWDDICA
VHEEMLELGIEPSIVTYNTLLDSFLKEGRKDKVAMLLKEMETRGSGCLPNDVTYNVVITGLTRKGDLEEAAELVEGMRLSKKASS
FTYNPLITGLLARGCVKKVYDLQLEMENEGIMPTVVTYNAMIHGLLQSGLVEAAQVKFAEMRAMGLLPDVITYNSLLNGYCKAGN
LKEALLLFGDLRRAGLAPTVLTYNILIDGYCRLGDLEEARILKEEMGEQGCLPNVCTYTILMKGSLNVRSLAMAREFFDEMLSKG
LQPDCFAYNTRICAELILGDIARAFELREVLMLEGISSDTVTYNILIHGLCKTGNLKDAKELQMKMVRGDRWSSWALHFKLETKR
EFEITFSELSCWVLSKRIVKHKHAFLLLSSRVRKMLDLRCKPVAEFVKASVKRRLSGKAQQGVLVALGWRGASGRSAQSSGRVRQ
QHRDMKRENHRRGREKNDNTGGEGERGERRRGGQRIEEGGGARGVGERERQRGGEACCADRVRVVAERDMRSVG
>GRMZM2G145412_P01
MAATAVTTPVGTGGAAAARSRFAAACGALSQYVKAAESERMHARPPAPLRPLPLMPGADVDASPADLDLPQAAPAPAPAQLTIVY
GGRVLVLADVPADKAAGLLRLAAGAAAERAETEAIGAKRRDHVSGSASAGADLPVARKASLQRFMEKRKARVAAVRTEPYRRPDV
SDSCPDNLKLAL
>GRMZM2G382794_P01
MPGADVGADEQQEPAAARAEEMPEPAPAPAAPPPLTIFYGGTVVVFEDFPAEKTAEVMRLAAGDDLPIARKASLPWFLAKRKDRL
VERAPYARPSSPAKEPEKKTFTFCLRIHMTGMAYDPLRTHLERRAAEQLDIFKEMVSLRVFERIFSELLYCEHWTTHYVFLQDYK
FILFFR
>GRMZM2G117513_P01
MAPAESGEKATSFAMACSLLSRYVRQNGAAAADLGLAIRAESDAKRTSTDMEKGETGKETMDLFPQIAGFGSEAATKEAPDAREP
EKRQLTIFYGGKVLVFDDFPAEKAKDLMQMASKGSPVAQNPGLLPPSTAATVTDSTKIAAVPAAPIAVANAQKSAADIPQAPKAS
LRRFLEKRKDRLTAKAPYPGSPSDVTPVKKEMSDGQPWLGLGPQIANPDLSLSKEASQ
>GRMZM2G314145_P01
MVVVFEDFPAEKAAEVMRLATGDDLSIARKASLQRFLAKRKACLVEHAPYARPSSPAEEPEKKTVKPASTLASWLGLGSTEADRL
TIALFCGSAHTRQVGPSLSVCFLLQLARRTAPAAPLPSFLSCSLPGLPHRLPQIVRDLGPPSPHSLPPPQPPSLGNLRLTLSSSA

PSTAPSLPMVPPDATRKGEGCGWGRSTSSRHWRSTWRWTARTTSSPQKGQLWHFGINGDYCWNFLVSETILFSLTQQRTCSGSSD
CF
>GRMZM2G116614_P01
MAGHAPARDKTTTGFAATCSLLSQFLKEKKGGLQGLGGLAMAPAPAAGAGAFRPPTTMNLLSALDAAKATVGEPEGHGQRTGGNP
REAAGEEAQQLTIFYGGKVVVFDRFPSAKVKDLLQIVSPPGADAVVDGAGAGAAVPTQNLPRPSHDSLSADLPIARRNSLHRFLE
KRKDRITAKAPYQVNSSVGAEASKAEKPWLGLGQEQEGSDGRQAGEEM
>AC197764.4_FGP003
MSAATGYSRFLRQYMREPQRHQLVEAEETDSGRTMQLFPTRAGGGTSSSSHEQSDDGAQAANKATLSIFYEGRMLVFEEFPADKA
KALMQLAAGSSGSSSAAAPNNKDAPVPVVRVRRVPEQPAATAPLAVVPSDLLPIARKVSLQRFLQKRKERIAATEPYPEPEKDDV
PATATATATGWQATALKDKPAPASWLGL
>GRMZM2G066020_P01
MPGADVGADEQQEPAAARAEEMPEPTPAPAAAPPLTIFYGGTVVVFEDFPAEKTAEVMRPAAGDDLPIARKASLPWFLAKRKDRL
VERAPYARPSSPAKEPEKKTNEHPNLCSPRFPRVWKSSGIAYDPLRTHLERRAAEQLDIFKEMVSLRVFERIFSELLYCEHWTTH
YVFLQDYKFILFFRCSKLSMIRKYLLFLCTKSKLQFEMMMCLCEVNADNNTIGCLRRPRSKWSSTSSTEILGASTTPVNIDKWIK
QQAIWIKSRGQS
>GRMZM2G086920_P01
MDLLERNIKTETEETRKEQERKEEEAEAEAEAGERKTQEPPQQGQGLSLSLANGSGRSGMLPMSNVSANPSQLTIFYGGSVCVYD
SVPPEKAQAIMLIAAAAAAAAATKSSAAPTVKPPMVPAATVAPAAVVSPVLTRSPSLQSTSVATGQPQVVAEPSSICKLQADLPI
ARRHSLQRFLEKRRDRVVSKAPYSTAKSSDGMESPRIEVTAEGKAQ
>GRMZM2G145407_P01
MPSTAPVELDFLGLRPAAAAAAEDDHHHGSSTTSAAAAASSSVRGMKTSAIASIGAHKLRRVIAGDEATKQHQQQQAPMTVFYGG
AVATFDGVSQDTAEAIMKMAMEVTASSGGRIVRRDAFRGNLTKDMPLTRTKSLQQFLQKRKERLSGPGPYLPASGGRSSRVGAAT
MTFHVKEEAA
>GRMZM2G143402_P01
MDRLEERNIKKGEREMEEARKEEERKEEAGDRNTQEPQQGQGLSLSLANGSDSGRSGMLPMSNPSANPTQLTIFYGGSVCVYDSV
PPEKAQAIMLIAAAAAAAAATKGSAATAFNPPMSTSVAAGQAQVVADPSSISKLQADLPIARRHSLQRFLEKRRDRVVSKAPYSP
AKSFDGMESAGMEMTADGKQGARPSILKGWGAAKSEV
>GRMZM2G151519_P01
MERDFLGAIDRAWKCGEAAKEESRSEPDSPAATRWQFAARSGVAPAFMSFRTEATGEGSEAFSVSELRPAGDAFDGIKKQSSMLS
IYQQQRQFGHNSSQATAQQQYPAAAHRQRSQVADYGAAAPHRLPGGGGGGCRLVQPVSVRHPVPFHQANLMVRSSVSQSFHNQQQ
PFTMSNNGFGGSTAGVYGARNPRNQASTQLTIFYNGSVNVFDNVPVDKAKELMMLASRASVPGATPSSEPDSPPVPAPAPAKVNV
AEVFPGARQIAVQKPEPCVPHLSSAATASPVRIVVPQAVAPSRSTSHCATEACGSKPAAAPTSQTVSSSRQLAAASAAAAVTPRA
VPQARKASLARFLEKRKERVASVEPYLTSNSKSPLESSDAVGSASAPTKSSSTDVAPASSHNGGGAELVRHGGYPRSISFSTNLQ
I
>GRMZM2G058479_P01
MSHHDGSKPYQPRRGPERHPQPADGIAAPPPAAVAPSVEHLVAAAAEAEALNRFAAEQQQQLQGHEQEVGEEEEEEDEQEDEMEE
EDEDEHEGQHGGIGGEHVPMDADAAAAAAAAAVSQMDPHSALVAGTVPPMATNQLTLSFQGEVYVFDSVSPDKVQAVLLLLGGRE
LSSLGGASSSAPYSKRLNYPHRVASLMRFREKRKERNFDKKIRYSVRKEVALRMQRNRGQFTSSKPKPDEIAASEMASADGSPNW
ALVEGRPPSAAECHHCGTNATATPMMRRGPDGPRTLCNACGLMWANKGLLRDVTKSPVPLQATQSAPHLDGGNGSAMSAPGSELE
NAAAAMTNGHESSSSGV
>GRMZM2G080509_P01
MSHSHHDGSKPYQPRRGPERPPQPADGIAVPPPAAVAPSVEHLVAAAAEAEALSRLGAEQQQLLQGHEQEVGEEEGEDEEEDEME
DDDDDDDEQEGQHGGIGVEHVPMDADAAAAAAVAAAGAQMDPHSVLVPGTVPPMATNQLTLSFQGEVYVFDSVSPDKVQAVLLLL
GGRELSSLSGASSSAPYSKRLNFPHRVASLMRFREKRKERNFDKKIRYNVRKEVALRMQRNRGQFTSSKPKPDEIAASEMAAADG
SLNWALVEGRPPSAAECHHCGINATATPMMRRGPDGPRTLCNACGLMWANKGLLRDLSKSPVPLHSIQQSAPILNGGNGSAMSAL
GSELENAAAAMGNGHEP
>GRMZM2G089736_P01
MAASARPGERATSFAVACSLLSRFVRQNGVAAADLGLRIKGEVEQQRTPATTNSLPGAEGEEVERRKETMELFPQSVGFSIKDAA
APREEQGDKEKPKQLTIFYGGKVLVFDDFPADKAKDLMQLASKGSPVVQNVALPQPSAAAAVTTDKAVLDPVISLAAAKKPARTN
ASDMPIMRKASLHRFLEKRKDRLNAKTPYQTAPSDAAPVKKEPESQPWLGLGPNAVDSSLNLS
>cassava4.1_015456m
MEAEADSYNEVKPKAGDCQEVSINKSVMDAATGADGDTGSLLLLRTNSSRPTAMATSGPNAAVPASDQLTIFYGGSVLVFDAIPA
ETAREIMLIAAAAAAAVKPAEVKKTVSGSPAGGTPVLTRSPSLQSATSALASPRAQPYPVHQGSSLCKLQAELPIARRHSLQRFF
EKRRDRLCSKSPYPTPPATKMAETTKPDFSAEVSPDAGCLGKPLAPEKEIQPKVAANVA
>cassava4.1_015933m
MEGDSDSFKEVKPKAGEEQPAINGADENMGSPMETNSSKPESMATSGPNATIPTSDQLTIFYGGSVLVFDAIPAAKVREIMLIAA
AAGKPADMKKAVSVSPAGGTPVLTRSPSLQSTTSALPSPQPQAKLHPVHQGSFLCKLQAELPIARRHSLQRFFEKRRDRLYSKSP
YLSSAERKMAETIKPDFSAQVSPDAGCFGKPLAPEKETLPKVAASVA
>cassava4.1_014096m
MANLPQKSSGNGKSVPEKSNFAQTCNLLSQYMKERVSFGDLSLGINGKPESKGPEASRPPATTLNLLTNIENPAETSRQNSVPSA
NIKPKEFFPQFVASASPNPTEDDSTVNKPADLRKSSKADPGTAQLTIFYAGQVIVYEDFAADKAKEIMALASKGSSSSKNGCTVS
TSAVDKSNNFTDPSNNNAPRGIQLHPQANGSDLPIARRASLHRFLEKRKDRVASKAPYQVNNPSSPARPTPDDESNPFIIDLEAQ
SSKQFDLNL
>cassava4.1_009349m

MMERDFLGLGSKNFPVTVKEEVTVTDGYKESVPMRGSAMQWSFSNKVSAIPQILSFKSGVEEKPRKPTHDRIASSGFIPISTADA
FDSNHKSYNSMVQKNMTLDKQGARQYTMTAYAAQHVDSHLFHHSQQTRIFPVTNHQNQTITISLSNPVLQSHLASVGNNVGGNSM
NSQSLAGVPSISPVSLHPTPSSIVGTTNLRNGPKPSGTPAQLTIFYGGSVCVYDDVSPEKAQAIMLLAGNGSSITQNKAVSSAQV
QAPIRSPSAGDGFIVNRIYASAPCLGLPSPISVTSSSANDLATGKPVGALVSANNHIESTKTVSSVGSGSATMIPAVAVPQARKA
SLARFLEKRKERVMNTLPYNVSKKSPDCSSATQCDSVSYSINSLSSPSHQ
>cassava4.1_008790m
MERDFMGLNLKEPLPLVKEEVNSDGYKEIGFNKGSGIHWPFSNKVSALPHLNSFKASQEDKAKRLVSDSSLSPGFLSISTADAFD
SKQKQFMAETQKSFNHDRQSGSHFTLTAYPVQHGVHSMHHPHDMKMFPFSNYASSISMSNPFFKNYQATSGQNIAGATLNPQLLG
GIPVVSPQTNLPTVGFVTGMTESCVKASRSPPQMTIFYAGTVNVYDDVSPEKVQAIMFLAGNGSSISSNMAQSNIQVQTPSSKPI
ATDVSPVNLSVTIPPCSRLSSPVSFSSQTGAQSGSGSTSTEEIMAAKNTGVATTPVSKLETPKISSAMGTVTATSMMPSAVPQAR
KASLARFLEKRKERMMISAPYNLGKKSAESAIQNPVMVSSATSAVGTCYLPASKEGNDNKQVRGNHCN
>cassava4.1_009865m
MERDFMGLNSKESSAVVKEEVNSDGYKEIGFSKGSGVHWPFSNKISALPHLNSVKVAQEDKIERVLTDSSVSPGFLSISTADSFD
HNQKQSMAETPTSNHDRQSGTHFTFTAYPVQHDVHSVHHPYDMKMFPFSNHATSISLSNPFFKNYYATSGKNTAGAVAKPEFGGF
PVTTPQTIIPTIGSVSGMSESCVKASGSPAQLTIFYAGTVNVYDDISPEKAQAIMFMAGKSSSIACNMLQSKCQVQPPSSKSIAT
DVSPAKHGATTPPCSRLSSPLSVSSQTGAQSGSGSTSTEEIVATKTTEVATTPVSKLDTQKLTSAIGSVAATTLMPSAVPQARKA
SLARFLEKRKERAMSAAPYNLGKKSPESAIQNPME
>cassava4.1_008816m
MERDFMGLNLKEPLPLVKEEVNSDGYKEIGFNKGSGIHWPFSNKVSALPHLNSFKASQEDKAKRLVSDSSLSPGFLSISTADAFD
SKQKQFMAETQKSFNHDRQSGSHFTLTAYPVQHGVHSMHHPHDMKMFPFSNYASSISMSNPFFKNYQATSGQNIAGATLNPQLLG
GIPVVSPQTNLPTVGFVTGMTESCVKASRSPPQMTIFYAGTVNVYDDVSPEKVQAIMFLAGNGSSISSNMAQSNIQVQTPSSKPI
ATDVSPVNLSVTIPPCSRLSSPVSFSSQTGAQSGSGSTSTEEIMAAKNTGVATTPVSKLETPKISSAMGTVTATSMMPSVPQARK
ASLARFLEKRKERMMISAPYNLGKKSAESAIQNPVMVSSATSAVGTCYLPASKEGNDNKQVRGNHCN
>cassava4.1_008829m
MERDFMGLNLKEPLPLVKEEVNSDGYKEIGFNKGSGIHWPFSNKVSALPHLNSFKASQEDKAKRLVSDSSLSPGFLSISTADAFD
SKQKQFMAETQKSFNHDRQSGSHFTLTAYPVQHGVHSMHHPHDMKMFPFSNYASSISMSNPFFKNYQATSGQNIAGATLNPQLLG
GIPVVSPQTNLPTVGFVTGMTESCVKASRSPPQMTIFYAGTVNVYDDVSPEKVQAIMFLAGNGSSISSNMAQSNIQVQTPSSKPI
ATDVSPVNLSVTIPPCSRLSSPVSFSSQTGAQSGSGSTSTEEIMAAKNTGVATTPVSKLETPKISSAMGTVTATSMMPSVPQARK
ASLARFLEKRKERMMISAPYNLGKKSAESAIQNPVMVSSATSAVGTCYLPASKEGNDNKQVRGNHCN
>cassava4.1_009889m
MERDFMGLNSKESSAVVKEEVNSDGYKEIGFSKGSGVHWPFSNKISALPHLNSVKVAQEDKIERVLTDSSVSPGFLSISTADSFD
HNQKQSMAETPTSNHDRQSGTHFTFTAYPVQHDVHSVHHPYDMKMFPFSNHATSISLSNPFFKNYYATSGKNTAGAVAKPEFGGF
PVTTPQTIIPTIGSVSGMSESCVKASGSPAQLTIFYAGTVNVYDDISPEKAQAIMFMAGKSSSIACNMLQSKCQVQPPSSKSIAT
DVSPAKHGATTPPCSRLSSPLSVSSQTGAQSGSGSTSTEEIVATKTTEVATTPVSKLDTQKLTSAIGSVAATTLMPSVPQARKAS
LARFLEKRKERAMSAAPYNLGKKSPESAIQNPME
>cassava4.1_014779m
MAIRIRKNMIPDKQGAKHYAVQHFDAYPVHRPQQMRIFPVSNHSQTITACHFASSGQNVGDNSLNSQSVGRVPIISSVSVHPTPS
SIVRTTELRNVSKSSVATAQLTIFYAGSVCVYDDICPEKAQAIMLLAGHGSSVTQDKTVSPAQVQAPIQGPSAYVGNKTHTCSGL
PSPIAVTSTSNNELATVKLMGPLASTNNSTEPPKTVGSSSATLIPTVAAPQARKASLARFLEKRKERVMNASPYNAS
>cassava4.1_013329m
MERDFLGLGLKNVQVTVKKEVADGYKDSAVPMRGSTMQLSFSNKKNMIPDKQGAKHYAVQHFDAYPVHRPQQMRIFPVSNHSQTI
TACHFASSGQNVGDNSLNSQSVGRVPIISSVSVHPTPSSIVRTTELRNVSKSSVATAQLTIFYAGSVCVYDDICPEKAQAIMLLA
GHGSSVTQDKTVSPAQVQAPIQGPSAYVGNKTHTCSGLPSPIAVTSTSNNELATVKLMGPLASTNNSTEPPKTVGSSSATLIPTV
AAPQARKASLARFLEKRKERVMNASPYNAS
>cassava4.1_014775m
MITYPLKNMIPDKQGAKHYAVQHFDAYPVHRPQQMRIFPVSNHSQTITACHFASSGQNVGDNSLNSQSVGRVPIISSVSVHPTPS
SIVRTTELRNVSKSSVATAQLTIFYAGSVCVYDDICPEKAQAIMLLAGHGSSVTQDKTVSPAQVQAPIQGPSAYVGNKTHTCSGL
PSPIAVTSTSNNELATVKLMGPLASTNNSTEPPKTVGSSSATLIPTVAAPQARKASLARFLEKRKERVMNASPYNAS
>cassava4.1_011377m
MERDFLGLGLKNVQVTVKKEVADGYKDSAVPMRGSTMQLSFSNKVSAIPQFLSFKSGVEEKPRKTTHDHISSGFSPISTADAFDS
NQNPYSCMIHKNMIPDKQGAKHYAVQHFDAYPVHRPQQMRIFPVSNHSQTITACHFASSGQNVGDNSLNSQSVGRVPIISSVSVH
PTPSSIVRTTELRNVSKSSVATAQLTIFYAGSVCVYDDICPEKAQAIMLLAGHGSSVTQDKTVSPAQVQAPIQGPSAYVGNKTHT
CSGLPSPIAVTSTSNNELATVKLMGPLASTNNSTEPPKTVGSSSATLIPTVAAPQARKASLARFLEKRKERVMNASPYNAS
>cassava4.1_011418m
MERDFLGLGLKNVQVTVKKEVADGYKDSVPMRGSTMQLSFSNKVSAIPQFLSFKSGVEEKPRKTTHDHISSGFSPISTADAFDSN
QNPYSCMIHKNMIPDKQGAKHYAVQHFDAYPVHRPQQMRIFPVSNHSQTITACHFASSGQNVGDNSLNSQSVGRVPIISSVSVHP
TPSSIVRTTELRNVSKSSVATAQLTIFYAGSVCVYDDICPEKAQAIMLLAGHGSSVTQDKTVSPAQVQAPIQGPSAYVGNKTHTC
SGLPSPIAVTSTSNNELATVKLMGPLASTNNSTEPPKTVGSSSATLIPTVAAPQARKASLARFLEKRKERVMNASPYNAS
>cassava4.1_013723m
MAGSPEFVEFAGLRAARFPEKSGFSQTCSLLSQYIKENGSFGDLSLGMTCSSEGNGTPEVRQAATTMNLFPVNERIDYVSSRNRA
TPRTNFISMDLFPQQAGFAPSVPKEDVQKSLDSSFSKAASPEPQNAQMTIFYGGQVIVFNDFPADKAKEVMLLAGKGSSQSLTGF
LPSVPVKSHPVFAPNVAKTPESISSVPPSSNAVPNFGNNLIQDRMQPPTQAIASDLPIARRASLHRFLEKRKDRITARAPYQTIG
FSGSPSPSKPAASKWLLNLGQ
>cassava4.1_031135m

MANLVQKSGKAASPEKSNFAQTCNRFSQYLKERGSFGDLSLGINGNLEAKAPEASRPPATTLNLLSNIENSAEVSSQKAMPFPNI
KPTDYFSQSVGFASTNPIQDSIAKSAGLRKSSRAADPGTAQLTIFYAGQVIAYDDFPADKAKEIMALASKASANTQNGFSTAAST
SAIDNIKSTLQLQPQAIGSGLPIVTRVSLHRFFEKRKERVASKAPYQLNNPSSSPPARPRRDEQIDLEDQSWQQLELKL
>cassava4.1_013620m
MAGSPEFVEFAARKASSFSQTCSLLSQYIKEKGSLGDLSLGMPCSGEGNGNGTPELRQASTTMNLFPMNEKQVDVCSRNMATART
NVRSMDLFPQQAGFAPSAPKEDVQKSLDSSINKAATPEPQTAPMTIFYAGQVIVFNDFPADKAKEVMLLASKGSSQSLTGFPSDS
VKSHPEGDPNVVKAPVESTNPVPPSSNAIPNFGNNLIQERVQPPSRSIGSDLPIARRASLHRFLEKRKDRITASARAPYQTVGLS
ASSSKPAESKSWLGLAGQSLQ
>cassava4.1_016877m
MSRATVELDFFGMEKQISAEPRFPKFLNRQRSFRDIQSAISKINPQVLKSVIASGSANLQNPENGYQCDSKKLFSVPSTPKEEQT
PFPHLPVYSPLQRPAVENPTETAPLTIFYNGTVAVFDVPRDTAESILKLVENGFSKSVESTNQQEVLKNTLDGDLPIARRKSLQR
FLEKRKERLTSLSPYACPPDCRL
>cassava4.1_016821m
MSRATVELDFFGMEKQISAEPRFPKFLNRQRSFRDIQSAISKINPQVLKSVIASGSANLQNPENGYQCDSKKLFSVPSTPKEEQT
PFPHLPVYSPLQRPAVENPTETAPLTIFYNGTVAVFDVPRDTAESILKLVENGFSKSVESTNQQEVLKNTLDGADLPIARRKSLQ
RFLEKRKERLTSLSPYACPPDCRL
>Medtr4g124360.1
MTTSNSINNIDINSISDQESVKHFSAANWPLSTSGLNAMTPGTTQFAILYNGSMCVYDGIPAEKVHEIMMMASANAKSSEMKSGI
PFNSLFSSTTPSSPQGNSDNLPSPPSVGFPAAEKSSICRMQEFPLARRQSLQRFLEKRRIRVRSKAPYTSSSSKAANNSDNNFSL
VMVSLEGK
>Medtr2g044910.1
MDGVTVKFEPEQFTVLESSPIAVDGASSNMVEGSMMNSSANKSMPASGMNPVIANTTQLTIFYNGSICIYDGIPAEKVQEIMRIA
AAAAKSSETKKIVKQSPAPSPVPTRPSSPHGTADNIASSQALPFPAKSSICRMQEFPIARRHSLQMFLQKRRDRLGSKAPYPSSP
KTKVADNMENNFGADNSPDSVSMKEPKEEFQPTISAS
>Medtr5g013530.1
MNHHNITPMNFQQFPHLFLQEIPILGNSSVMKANIKKEEPSCAQMTIFYDGKVIVFDDVPADKAKDIMDFSTKGIASTSQNHNNN
YAYSSFLSRNSLQDYPQVPSIPVIYDLPMTRKASLHRFLEKRKDRIAAKAPYQTSNPAAFLNKPIDESMSWLSLAPQSECSSTSV
LFL
>Medtr2g042900.1
MSTSSEYSEVSGNKPPAKSPEKTTFSQTCSLLSQYIKEKGCFKDLSLGITCNNNNTDPSGSSETSSQSATTMNLFPTMENNLSQK
NLTTMDLLTPQAALNNSNAIKGPKAAQLTMFYNGQVIVFDDFPADKAQELMAFANKGISQSQNNSVYTYTQSQPSFPPNLVRTSV
NTTTPIVPTVNIIPSTATGTGSMNEHLQVPSRPNLCDLPIMRKASLHRFLEKRKDRIAANAPYQINKPAESMSWLVGAKSTQI
>AC233676_11.1
MERDFLSLCSKESSPEINNEGSKNSGFSNVSAVKWPFLNKVAVHSYLTPFKVSEDDKAKMISSGFIQNSFKHDGQVGIHFSVNQY
PVQHNVNFMNRHHDVKMFPISNQANSLSAVHPLLKNHLATFGQNINGANAKQSLLGGLPVTAPHSVLPIVGTVAGLVEPCEKPSA
PAPQLTMFYGGTVNIFNDITPEKAQAIMLLAGSGLSAASNRAQPEVQASSSKFASGDDGLPISPPVYIPPCSGISSPLSVSSHTG
PQPGSGSSSSDEFMAAKTSRGPTPTTSACKVVTPKVVNATTMIPSAIPQARKASLARFLEKRKERVMSTAPYNLNKKSEDAQMPN
SMGANISATTGTANMLVANQG
>Medtr6g069870.1
MKAANIVYVFGIHSWFASPLLCLGVGVNINYVLKFDAVRSSGMQWSFSNKVSNLPQFMSFKNNTHEDRSRNNVMDPVASSGYMTI
STKDAFDSNQKSFLGVTQENLAIKQVGNKHGITIYPIQSSDAQSVCNQEARTISVSNQSNHVITGINMVNSVTNSQTFGSKSSAT
PLSVLPSKGPIVGSTDLRSRNCSKSNGTTPAQLTIFYGGTVCVYDDISPEKAKAIMLLAGNGTKMQQEISIPSKKDNFIISQPYP
SPLPSPIPMTSHASTQPRGGSSSNNEVTIIRTLGHSIAKSSHNDLSHLSSLPSPLPMTSHASSYPKGGSSSNNEVTIIRSLGPSN
APTNHLESPIVATSVGLTPTNVIQPVGLPQARKASLTRFLEKRKERAMSTSPYYMSKISPECSTGSDNASFSIDFSGSSTQPPTN
LPLRRTCMEVIR
>Medtr5g013520.1
MTPMNFQQLPHLFLQEIPILGNSSVMKANIKKEEPKCAQMTILYDGKVIVFDDVPADKAKDIMDFSTKGITSTSQHHNNNYAYSS
FLARNSLQDCYQVPSIPAIYDLPMTRKASLHRFLEKRKDRIAAKAPYQTSNPTTINKPIDESMTWLSLAPQSPQHKSECSSSSTH
>Medtr4g124960.1
MSTSSDISGLSGNKLTKSSEKPTFSQTCNLLRQYLKEKKGSFEGFNLHTPETNGSSPGSSSHSGITMDLFPTNVTPKNLTTMDFF
FPRVVNPMVKEPETAQLTMFYNGQVIVLDDFPAEKVEELKSFARTQTQHSDVPTMIPQQPPSLIDMPIARKASLRRFMEKRKDRV
SVYSPYQRICPDSAAPEKHAESAPWLVLGAKST
>Medtr4g124950.1
MDLFPTNVTPKNVTPVDSLSPRINNSMVKELETAQLTLFYNGEVIVLDDFPAEKVEELKSFASTLKLGLQLFLIGKASLLRFMEK
RNVRVSAKSPYQRSDFDSAPKKTY
>Pp3c2_590V1.1
MSDAEPLVHPQSLQLHHSLHPHNLNHGLSHAQHAMHEMHVHGHGEADGHGHVRVDQRVQGHLEGDHAHGNHGHHGHGMHNNEENE
AEVEDHDDDAADEEGLDEANMHSDGGGNPNDGPAPLTVRTQSSTQLTLSYQGEVYVFDAVPPEKVQAVLLLLGGREIPPGMSGVN
ISNNHHHHKGLTDLPARMNMPQRLASLTRFREKRKERCYDKKIRYTVRKEVAQRMQRKKGQFASSRPSQEEGAPVANWDGTQALG
QPVGAGGVQPEVICVHCGIGERSTPMMRRGPAGPRTLCNACGLMWANKGVLRDLSKNLSIAPGVQQQLILQSQQIILQQLQHQVA
GSQHSAENTSQVVDVQGSLQGEVDVQKNIAGIVAAGGGPVLAAG
>Pp3c17_23200V1.1
MRTEEVDVGGMSNADHLVHAQSLQLHHNIHQHSLNHALGHAQHGMHEMHVHGHGHGEADGQGRVDQRVQGHLEGDHVHGHHGHGM
HRGEENAAGVEEHDDDAGDEEGLDEADMHSDGGGGNPGDPPVALAARTQGSTQLTLSYQGEVYVFDTVPPEKVQAVLLLLGGREI
PPGMSGGNVSSHHHHKGMPELPTRMNMPQRLASLTRFREKRKERCYDKKIRYTVRKEVAQRMHRKKGQFASSRPTQEEGAPAANW

DGTQASGQLLGPGGVQPEVMCVHCGIGERSTPMMRRGPAGPRTLCNACGLMWANKGVLRDLSKNLPLAAGGQQQLMLHPPQIILE
QQQHQIAGLQHSVENVSRVVDVQMGSLQRGVDDQKNMASMVAVGGGPVVAAG
>Pp3c14_24090V1.1
MRPEEVDVGGMSDVDHLVHGQSLQLHHNIHQHSLDHALGHAQHGMHEMHVHGHGHGEADVQGRVDQRVQRHLEGDHVHGHNGHHG
HGHGMHHNEENGAGVEDHDDDAGDEEGLDEAEMHSDGGGNPGDAPPALAIRTQGSTQLTLSYQGEVYVFDAVPPEKVQAVLLLLG
GREIPPGMSGGNVSSHHHHKGMPELPSRMNMPQRLASLTRFREKRKERCYDKKIRYTVRKEVAQRMQRKKGQFASSRPTQEEGSP
VSNWDGTQASGQPLGPGVQPEVSCVHCGIGERSTPMMRRGPAGPRTLCNACGLMWANKGVLRDLSKNLPMTAGGQQQLMLHPQQD
TDLSAATAGPSGGLAA
>Pp3c1_40750V1.1
MPSEGVDVGGMSDGDHLVHAQSLQLHHNLHPQSLSHGLGHAQHAMREMHVHGHGESEGHGQARGDQQVQDHLEVDQVHNHHGHHG
HGMHNKEENGVEVEEHDDDAGDEEGLDEADMHSDGGGNPGDGPTPLAVRTQGSTQLTLSYQGEVYVFDTVPPKKVQAVLLLLGGR
EIPPGMSGVNMFSHHHKGLTELPARMNMPQRLASLTRFREKRKERCYDKKIRYTVRKEVAQRMQRKKGQFASSRPTQEEGAPVAN
WDGTLAPGQTVAPGVHPEVTCVHCGIGERSTPMMRRGPAGPRTLCNACGLMWANKGVLRDLSKNLSITAGGQQQLMLHPQQILQQ
QQHQVAGLQGSVDNAPQGVDVQGSQQREGDVQKDMASIVTADGGPVLAAG
>Potri.006G217200.1
MTFVASDLVPMGGQSNSCKEMKPQEDIEVKKEQEVTNGCGENLVSCKEGADLLEKKGVHPLWTPSSRPTIMATSGPNATIPTPDQ
LTIFYGGSVVVFDAIPAEKVQEIMLIAAAAAAAVKPVDMKKSGSPDGTPVLTRSPSMQSTAAPHAQAYSRQNSFCRMQAELPIAR
RHSLQRFFEKRRDRLVSKSPYPTSPEGKEADTTKPGISAAPSPDAGCFGKSLASDELQPKMSSAVVVKIFWLLLDALDSMYNFSL
KLVGFCSHVCPLQQKALVMSSEPAPFGRLLVLL
>Potri.018G047100.1
MEAQQPDSRKEMKPREDMEVKKEQEVMGSCKEGAGLLEKNGVHHLWAPSYWPMTMATSRPNVTIPTPDQLTIFYGGSVVVFDSIP
AEKVHEIMLIAAAAVKPGDMKKSGSPTGTPVLTRSPSMQSTAAPQGQTYSRQNSICRMQAELPIARRQSLQRFFKKRRDRLVSKS
PYPTSPAGKEADTTEPGISAAPPPDAGCFGKPLASEELQPKVAANVV
>Potri.008G133400.1
MHHPHDVKMFPVSNHAIPISMGNHFFKNHYPATGQNMAGTTTKPQLLGGIPVTAPHSILPMVGSVAGVTDSSVRASGSPAQLTIF
YAGSVNVYDDISPEKAQAIMFLAGNGPSISSNLAQPIVQVQASSSKPAADLSPVNQPIMSTPPCSRLSSPSHTGAQSGSGSTST
EEIMATKTTGALTTHVTKPEHTKTANVVGSVTTTTMIPSVPQARKASLARFLEKRKERVMNAAPYNLNKKSPHFTNPEPY
>Potri.015G035800.1
MERDFLGLGSKNNPVTIKEEATDTPLKDSVPMRGSGMQWSFSNKVSAIPQFLSFKSSMEDKPRKAVHDPMASSSSGYMSISTADA
FDSNQKSYSALIQKNMALDKQAGNHYAMTTYGKQHFDAYFANRPQDMRMFPISSQQNQTINVSMSSPILQSLFPPTGHSMITSNS
IVSKPLGGVPVITPASALPTPSSVIGTTDLRDVAKSSGAPAQLTIFYAGSVSVYDDVSPEKAQAIMLLAGNGGSSGTQNKPISTP
QAQAQAPIPGPPVGDIFVGNKINTTAPCSGMPSPISVTSSSTNDLAIVKPVVNLAPSVKHIEPTKPASSVGPTSATLVPAVAVPQ
ARKASLARFLEKRKERVMQTSPYNGSKKSPEGGAHRFDGMSLSMSTSSSFPLPASN
>Potri.012G044900.1
MRGSGMQWSFSNKVSAIPQFLSFKSSLEDKPRKAVHDPVASSSGLMSISTADAFDSNQKTYSGLVQKNMAIDKQAGNHYPVTTYG
TQQFDAYSVNRPQDTRMFSISGQQNQTITVSMSSPILQSHFPPTGHNMLSNSIVPKPLGGVPVITPTSALPTLSSIIGTTDLRDG
TKSSGAPAQLTIFYAGSVCVYDDVSPEKAQAIMFLAGNGGSSGTPNKPISTPQAQAPIRRPPVSDIFAGNKSNTTAPISCIPSPI
SVTSSNTNDLATVKPVVSLASSVKQTEPPKPLNSPGPTSATLVPAVAVPQARKASLARFLEKRKERVTQTSPYNVSKRSPESGSH
GCDGASLSMNFSSSYPLPASN
>Potri.006G139400.1
MSGSTEFVENMGKMCEKPSFSQTCSLLSQYLKERGSFGDLNLGMASNSESTPNKNGPSEMLRRSPSTMNLFPVSEKPGHISCQNM
GAPRNFTSMDLFPQQAGFAPKEDVPMKLDSRCVKSATAEPQTAQMTIFYAGRVIVFNDFPADKAKEVMLLASKGSSQIQNAFPSI
PANSHPALAPNISKTPIESTISIPSSSNALPNFGNNLIQESMQPAPQPIANDLPIARRASLHRFLEKRKDRIIAKAPYQINPAAT
TSKPAESEFSWLGLAAPSTTH
>Potri.003G165000.1
MKMSRGTVELDFFGMSKENRSSSSKSKCFNRQRSFRDIQSAISKINPELLKSVIASGSASNKATPANGNQLSNKAFSVPSTPKQD
LPPFPALPVYFPLPRLNLENPPETAPLTIFYNGTVAVFDVPRDKAENILKLAEKGFSKTVVESVADPRTDHQQKLLESLDGDLPI
ARRKSLQRFLEKRKERLTSATPYACTPKTRF
>Potri.001G062500.1
MSRAAAVELDFFAMGKENKSSPSKSKFLNRQRSFRDIQSAISKINPELLKSVIASGSAATKTTPENGNQFSNKSFSVPSTPKQEQ
PSFPALPLYSPLPRPNMMIPPETAPLTIFYNGTVAVFNVPRDKAENILKLAEKRISKTSAEPMTDPKTDQQQLFESLDGADLPIA
RRKSLQRFLEKRKGRLTSVSPYVCNT
>Potri.003G068900.1
MANMAQKSGKPQDQISNFAQKCNLLSQYLKERGSFGDISLGINGKAEIKGLETPSSPATTLNLLNNMEISSDQITSRQNAMASAN
MMKFMDFFPQFVGSGPPDSTDDAINKADHLRKSSPMDPETAQMTIFYAGKVSVFNDFPADKAKEIMALAAKGSSISTDGCPSSAP
AIRKVSSTNSVAALDSNKGQERLQLQSQANASDVPHARRASLHRFFSKRKDRVAARAPYQINNPTPDHPRPPRSEEDSNPFLALD
EGQSSEQLELKL
>Potri.001G166200.1
MANLAQNSCKSPDQISSFAKKCNLLSQYLKEKGSFGDISLGINGKAPEVKGPETSDLPATTLNLLTNMENSSEHITFRQKPVASS
NMMKYMDFFPQFVGFSPSNYTEDAINKADNHLRKSSTMDPGPTQMTIFYAGKLTVFNDIPAGKAEEIMALATKGSSISPNGFPSD
PSIIKVNSANSVAALDSNNAQQRLHLQSEAPNGSDVPHATRASLHRFFSKRKERVTARAPYQTHNPTHDLPSSSRPKEDSNSLLA
LNEGQSSKQLELKL
>LOC_Os04g55920.1 OsJAZ1
MDLLEKKNIKKGGEVEEEVARKGEERKEEEVVVEEKSHQQQQQQGEEELVGLSLAGGRPK
VFPMSSPPPNPSQLTIFYGGSVCVYDSVPPEKAQAIMLIAAAAAAAASATKSNAAIAVKP

PVMPAANATQAAVSPVLTRSLSLQSTSVATGQPQVAADPSSICKLQADLPIARRHSLQRF
LEKRRDSRLVSKAPYPTKSSEGMEASGMEVTAEGKAQ
>LOC_Os07g05830.1 OsJAZ2
MAEERRRDDGGDVEVELSLRLRTGDDSTSADPAPATVAAEARRNLTIFYNGRMCAVNVTE
LQARTIISMASQGNFGKQQQQQIQGRDDHHYHQGESSSGGGVSTAAARHCDVAGSSSSHS
GSGSGSATPPRPALVSPRAGLQAAAAAAPTMNQPPAASGLSMKRSLQRFLEKRKTRAAAP
LYARR
>LOC_Os08g33160.1 OsJAZ3
MERDFLGAIWRKEEAAGKPEEHSVSRDADYRGGGGGASAAMQWQFPATKVGAASSAFMSF
RSSAAAAREEDPKEAAVFDRFSLSGFRPPPRPSPGDAFDGAAAMKQRQFGFNGRQQYAAA
AQHGHREQGVDSYGVAAPHHFPSPSPSPRHPVPFGHANPMLRVHSLPNVAGGSPYRNQSF
SVGNSVAGSTVGVYGGPRDLQNPKVTQMTIFYDGLVNVFDNIPVEKAQELMLLASRASIP
SPPSAARKSDSPISAAAKLTVPEALPARQIVVQKPEASVPLVSGVSNPITIVSQAVTLPK
SFSSSNDSAGPKSGGLPLAVTPLSQASPSQPIPVATTNASAIMPRAVPQARKASLARFLE
KRKERVSSVAPYPSSKSPLESSDTIGSPSTPSKSSCTDITPSTNNCEDSLCLGQPRNISF
SSQEPPSTKLQI
>LOC_Os09g23660.1 OsJAZ4
MERDFLGAIGKDEEQRRHAEERKESDYFGAGGGAAAAAMDWSFASRAALMSFRSSSSAAA
AAAREETRELAFPHFSALDGAKMQQASHVLARQKSFGAESHGIPQYAAAAAVHGAHRGQP
PHVLNGARVIPASSPFNPNNPMFRVQSSPNLPNAVGAGGGAFKQPPFAMGNAVAGSTVGV
YGTRDMPKAKAAQLTIFYAGSVNVFNNVSPEKAQELMFLASRGSLPSAPTTVARMPEAHV
FPPAKVTVPEVSPTKPMMLQKPQLVSSPVPAISKPISVVSQATSLPRSASSSNVDSNVTK
SSGPLVVPPTSLPPPAQPETLATTTAAAIMPRAVPQARKASLARFLEKRKERVTTVAPYP
LAKSPLESSDTMGSANDNKSSCTDIALSSNRDESLSLGQPRTISFCEESPSTKLQI
>LOC_Os04g32480.1 OsJAZ5
MSTRAPVELDFLGLRAAAADADDRHAKSGGSSASSSSSIRGMETSAIARIGPHLLRRVIA
AAGPPPPPSTAPVPEEMPGAAAAAAPMTLFYNGSVAVFDVSHDKAEAIMRMATEATKAKG
LARGNAIVGNFAKEPLTRTKSLQRFLSKRKERLTSLGPYQVGGPAAVGATTSTTTKSFLA
KEEEHTAS
>LOC_Os03g28940.1 OsJAZ6
MASAKSGERGSSSFAMACSLLSRYVRQNGAAAGELGLGIRGEADANKGKETMELFPQNSG
FGSEAAAVKETPDAREQEKRQLTIFYGGKVLVFDDFPAEKAKDLMQMASKSSSTAQNCVL
LPSSATATVADNTKVSAVPAPASALPVAQANAPKPVRPNAADLPQARKASLHRFLEKRKD
RLQAKAPYQGSPSDASPVKKELQESQPWLGLGPQVAAPDLSLRQESSQ
>LOC_Os07g42370.1 OsJAZ7
MAASARPVGVGGERATSFAMACSLLSRYVRQNGAAAAELGLGIRGEGEAPRAAPATMSLL
PGEAERKKETMELFPQSAGFGQQDAITADSAADAREQEPEKRQLTIFYGGKVLVFNDFPA
DKAKGLMQLASKGSPVAPQNAAAPAPAAVTDNTKAPMAVPAPVSSLPTAQADAQKPARAN
ASDMPIARKASLHRFLEKRKDRLNAKTPYQASPSDATPVKKEPESQPWLGLGPNAVVKPI
ERGQ
>LOC_Os09g26780.1 OsJAZ8
MAGRATATATAAGKDRSSFAVTCSLLSQFLKEKKGGGGGLQGLGLGLRPAPAAPPAAGAG
GAFRPPPTTMNLLSGLDAPAVEVEPNTAETAADELPLIKAPADQQSDESASEAAGEKAQQ
LTIFYGGKVVVFENFPSTKVKDLLQIVSTGDGVDKNTGTAATQSLPRPAHNSLPDLPIAR
RNSLHRFLEKRKGRMNANAPYQANCTAAPSKQANGDKSWLGFGQEMTIKQEI
>LOC_Os03g08310.1 OsJAZ9
MASTDPMTRRFAVACGVLSQYVKANSSQPSTAAPVAQGVSGLMAAAAAAAAAPVVQEPGC
EVDGGGQQFTIFYAGKVVVIDRCTPAMAAELMRFASAAQGGGGAPEAPPALVDMPIARKA
SLKRFLAKRKATPASARSSYVVRAAAAEEEQPPAKKAKAAVERREDWLALGSLGHMHSR
>LOC_Os03g08330.1 OsJAZ10
MAMEGKSRRFAVACGVLSQYVRAEQKMAAAAGAAPARAVTTLSLMPGAEVVVEEEERREV
GEEEAGPATAPAAPLTIFYGGRMVVFEDFPADKAAEVMRMASSGMAAAPAQREGAALADM
PIMRKASLQRFFAKRKDRLAATTPYARPSPAETKASEPEEKKTPTSWLDLAASASAAARR
DSLTIAL
>LOC_Os03g08320.1 OsJAZ11
MAGSSEQQLVANAAATTVAGNGSRFAVTCGLLRQYMKEHSGSNGGGGFLPAVTAMSLMTG
GADAEEEAPEVRKTMELFPQQAGTLKDTQERKEITEKAQLTIFYGGSVVVFDDFPAEKAG
ELMKLAGSRDSTAAAAVSDAGAAAGQPCLPDMPIARKVSLQRFLEKRKNRIVVAEPLPES
EKKEAESSKRAKKDDGGASWLQVNPTLSL
>LOC_Os10g25290.1 OsJAZ12
MAAAGSSSRFAVTCGLLSQYMRERQQPQPPVTVLEAVAEEEEEEDARTMQLFPPRAAAAD
GVATPSAGTAPLTIFYDGRMVVVDDVPVEKAAELMRLAGSACSPPQPAHAAALPEMPIAR
KASLQRFLQKRKHRITTTSEPYKKAAVASPAPEKSFAVAPVKDEPATWLGL
>LOC_Os10g25230.1 OsJAZ13
MAAEAAATSRFAAACGALSQYVRAADNVHRARTAAAAAAVRPLPLMPGADVAGDEREEEG

GGAAASSAAAQMTIFYGGRVLVLDECPADRAAALLRLAASSRGVPRDDLASTAAAAGESA
DLPVARKASLQRFMEKRKGRLAARGQPYRRHDAAAAARGDHLALAL
>LOC_Os10g25250.1 OsJAZ14
MAVSDHHCGGGGRSWRFAVACGVLSRCVKAEAAAAANGRHRHHPTMLLMPGADVEPDVRE
EAAAAAQLKIMYGGRMLVFDDFFPAGGAVVELVRAAARAGQDVRRAGAARRRVGDSRGLD
AGLPVVRKVSLQRFVEKRRRMRVYHILYTDKSSHHVPGPGRYRSWQCRIIIAAVAGAGGF
VVACGVLSRCVKAEAAAAAANGRRHHHHHHTTMLLMPGADVEPDVREEAAAAAQLKIMYG
GRMLVFDDFFPAGGAVVELVRAAARAGRDDDGARARRRPAGGEEGVAAAVRGEEKSQAAR
GDGAVHTRHSPPMLPARTPGSGRTDDAAFY
>LOC_Os03g27900.1 OsJAZ15
MDAVGAAGGGAMLPAAARRGQPPQPPCMTTAPEQQAAAGGAVIWPAAAAAEAKEKMVVDA
RTMQLFPTRSADGVVVSPAPAPAAAQERRRPEVHVTPSVPATAPTAPLTIVYGGQVLVFE
HYTAEAAEKLVQRTQHLLAAAAGGGGGNKNNNVTVVTPPPDEPPMLLPPPQMPAASGVSA
GGVMPIARKASLQRFLQKRKQK
>29653.m000290
MRIFPVSNQQNQTITVSMSNPVLQSHFTSTGHNMISNAMNSLSLEGVQSLSPASVHPTPSSIVGTTDLRNGSKSPGAPAQLTIFY
GGSVCVYDDISPEKAQAMMLLAGHGSSVTQNKMFSTAQVQAPITRASAGDGYIENKVHTTSPCSGLPSPISVTSSSPNELAAVRS
VGALASGSNQTETPRAITSVGPGSATLIPAVAVPQARKASLARFLEKRKERVMNASPYNVSKKSPDCAASGSGNVSLSISSFSSS
SHQ
>29962.m000052
MERDFLGLNCQDSLAVVKEEVNSDGYKEIGFSKVSGIQWPFLNKVSSLPHLMPFKAAQEDKTKRIVSDSIVPSGFLSIATVDALD
PSQKQVAEIQRSFNLERQGGNHFTLASYPLQHDVHSVHHPDVKVFPVPNYTSSISTSHSFFKNHYATTGPSMVATTTKPQFLGGI
PVTAPQTILPNISSVNRKFDSCVKTSGSPAQLTIFYGGTVNVYDDISPEKAQAIMFLAGNGFSIPSNMSQPKIQVQAPNSKAVAT
VVSPVNQPVITPPCSRLSSPLSVSSHTGAQSGSGSTSTEEIVAVKPSGVPTTPVSKPDTPKLASAMGSAPAAIMMPSVPQARKAS
LARFLEKRKERMMSAAPYNLCKNSLESSIQNPMD
>30128.m009047
MWNATVELDFFGMEKDNCSKSRFPKFNRQRSFRDIQSAISKINPELLRSVIVSGSANQKNAENGNQFDSKMFPALPVFSPLPRPT
LETPPQTAPLTIFYNGAVAVFDVPRDKAETILKLAENGVSKSAESTSQKHLLDNLDGDLPIARRKSLQRFLEKRKERLTSASPYG
WLPDSHL
>29765.m000754
MEESEVFIRRKHSGGPLLPPKFGSHIPRHASFLEQKLLPGIRSHPMQSQSNGKSSSTAQLTIFYAGEVNVYDNIPADKAQAIMLL
AGESCVSKPMATEKPKAEVKKPTDSTSACKLQTDLPIARKLSLQHFLEKRRRRRTGKSPYSTQHQESEPREDNKDSNDQPISLSP
FASRLG
>29739.m003659
MEGESFSQEEVKVKAVLEEVKKGEEVVNGGDGNMGSCTETLDPWTSALRPASSGPNATISTPNQLTIFYGGNVLVFDAIPAEKVR
EILFIAAAAAAAVKPADTKKTAAVSPASNTPVLTRSPSLQSTTSALPSPQAQLYPIHQASSLCKMQAEFPIARRNSLQRFFEKRR
DRLGSKSPYPTPAAKMAGAKKPDLSTEVSRDASSIAQDKEIQREVAANLA
>29739.m003727
MAGSPEFVGLSGQKTARLSEKNSFSQKCSLLSQYLKEKGSFGDLSLGITCNNSNNNADAKINTGNGNGASDMIKQTTTMNLFPMS
EKHVDVPNRNMVTNCRSMDLFPQQSGFVTTTPEPKEDMQKRADSSVHKPASPESQNAQLTIFYAGQVIVFNDFPADKAKEVMLLA
TKGNSLNRFPSVPVKSHPPAFAPSVSKAPAESNSSLSSASNAVLNFSNNLIQERKLTPPPTIGSDLPIARRASLHRFLEKRKERI
TASAPYQTSGLPAIPPKPAAESKSWLGLAAQSSH
>29727.m000494
MAHLVQRSGKAVATEKTNFAQTCNLLSQYLKERGSFGNITYGITSKPEANKGPEASRTPATTLNLLPSMENPAENSSRQDYVPST
NIKPMELFPQLVGFSSQNPVEGSTNKAADLRKSSKGDSTTAQMTIFYGGQVMVFDDFPAEKAKEIIALASKGTSNTTNGFTTASA
VEKANQSAIAPPPNKVREGLQLRPQADDSDLPIARRASLHRFFEKRKDRAAAKAPYQINNPSSSPPPPPPPRPRHEGERNIVFID
LECQTSEQQLDLKL
>Solyc07g042170.2.1
MASSEIVDSGRFAGQKSHFSHTCNLLSQYLKEKKGSLGDLSLDMHRNFDSAGSTTMDLLPMIEKSGELVQKSMNLFPQGGMKAES
EPEKAQMTIFYGGQVIVFNDFPADKAKEIMLMASTSKGNNPAKPLESAADLVVPSFGKTSIQENQMPNQPIVSDLPIARRASLTR
FLEKRKDRLTAKVPYHREEAAAPKKEEHKAPWLGLGGQFAVKTEQY
SEPEKAQMTIFYGGQVIVFNDFPADKAKEIMLMASTPIARRASLTRFLEKRKDRLTAKVPYHR
>Solyc12g009220.1.1
MGSSENMDSGKVTGQKSQFSQTCNLLSQFLKKKGSVGDLNNLGIYKTTFESTGSQQTATTTTTTMNLLPMIEKSSDSSSSSSSVE
TNPQKPMNLFPQEFDFSKEQSTKKTESWKPDQPEKAQMTIFYGGQVIVFDDFPADKANEIMKLANKQNPTNNFTYPMIKNQKTAD
QSGVSFGNKLIQELPKLSMPQPSVADLPIARRNSLTRFLEKRKDRVTSIAPYQISNNKKSKNEDNKAWLGLGAQFVKTEQYFDQP
EKAQMTIFYGGQVIVFDDFPADKANEIMKLANKPIARRNSLTRFLEKRKDRVTSIAPYQI
>Solyc12g049400.1.1
MSNRQLCSLDSEKSHLMNTCNLLTQFFNGKANINDLNLTISNNGEAKASATKDLLTNMEELSTKTTEQDQKLIDHVPKSAINKAS
GSKEIPHKEQKLAQLSIFYGGKVVVFDDFPAEKARAVMLLASKGISNNSCAIFQTPTTTQTNGSNNFDLPIARRSSLYRFLEKRK
DRDTARAPYQMHNPLQSSSRTRGDHFDLNF
KEQKLAQLSIFYGGKVVVFDDFPAEKARAVMLLASKPIARRSSLYRFLEKRKDRDTARAPYQM
>Solyc03g122190.2.1
MSNLCDARRRNGNGKAPERSSFVQTCNLLSQFIKGKATIRDLNLGIAGQPEAAGKTETATMDLLTVMEKPSIDLTKEEHKSVDLV
TTESSREKEAAVNEPSTSKEAPKEPKAAQLTMFYDGKVIVFDDFPADKARAVMLLASKGCPQSSFGTFQAINIDKINTCSPAPAS

LTSNRTDSVAPQQQHLQIKPDSCSAAPQQHKHNSPPLHVCSSTKTDQLKLGSVSSAPLVEQEQHKQIQSQAAEISSSSELPIARR
SSLHRFLEKRKDRATVRAPYQVVRNNPLLPSSSNTNGESSSKDSEDQLDLNFKLKEPKAAQLTMFYDGKVIVFDDFPADKARAVM
LLASKPIARRSSLHRFLEKRKDRATVRAPYQV
>Solyc01g103600.2.1
MRIPRHDDTKDPVSQRVTSESEQLTIFYAGIVHVYDNISVQKAESIMNLASENCNAKEIKPTQKSQVPHHVYKFQAELPIARRKS
LKRFFEKRHSRITSKQPYASPECDHDQSENWNDTKKINITHAQEVTSESEQLTIFYAGIVHVYDNISVQKAESIMNLASEPIARR
KSLKRFFEKRHSRITSKQPYAS
>Solyc06g068930.1.1
MHWSYSNKAHPQYHLSLKDQENIIINNNNNNNKPKIGFESLASAGLVTITTTTELFDTIHRPYTTQFGAHHVPTRNGVVGTTELRG
TPRPSPGPAQLTMFYAGSVCVYDNISPEKAQAIMLLAGNTPISTTIRNSPSLDHHHHHHHNNNNNNNNNNSTNETTIIRSIGVLKS
HELSKIVTSQESRQPPNHNLSAVPQARKASLARFLERRKERVVSASPYGNGKQSSQHMMNFTINSSGSSTSLPAAN
PSPGPAQLTMFYAGSVCVYDNISPEKAQAIMLLAGNPQARKASLARFLERRKERVVSASPYG
>PGSC0003DMP400005281
MGSSENVDSGKVTGQKSQFSQTCNLLSQFLKKKGSVGDLNNLGIYKTTFEPTGSQQTATTTTTTTMNLLPMIEKSSDSSSSSSSVE
TNPQKPMNLFPQEFDFSKEQSTKKTESWKFDQPEKAQMTIFYGGQVIVFDDFPADKANEIMKLANKQNPTNNFTYTMMKNQKTSD
QSGANFGNKLIQELPKCQVSMPQPSVADLPIARRNSLTRFLEKRKDRVTSIAPYQISSNKKSKNEENKAWLGLGAQFVKTEQYF
>PGSC0003DMP400005280
MNLLPMIEKSSDSSSSSSSVETNPQKPMNLFPQEFDFSKEQSTKKTESWKFDQPEKAQMTIFYGGQVIVFDDFPADKANEIMKLA
NKQNPTNNFTYTMMKNQKTSDQSGANFGNKLIQELPKCQVSMPQPSVADLPIARRNSLTRFLEKRKDRVTSIAPYQISSNKKSKN
EENKAWLGLGAQFVKTEQYF
>PGSC0003DMP400039689
MSNLCDGRRRTGDGKAPERSSFVQTCNLLSQFIKGKATIRDLNLGIAGQTEAAGKTETATMDLLTVMEKPSIDLTKEEHKSVDLV
TTESSREKEAAVNEPSTSKEAPKEPKAAQLTMFYDGKVIVFDDFPADKARAVMLLASKGCHQSSFGTFQTTNIDKINTCTTAPAS
LTSNRTDSVAPQQQHLQIKPDSSSAAPQQHKHNLPPLHVCSSTKTDQLKRGSVSSAPLVEQEQHKQIQSQAAGISSSSELPIARR
SSLHRFLEKRKDRAIVRAPYQVVHNNPLLPSSSNTNGESSSKDSEDQLDLNFKL
>PGSC0003DMP400007763
MSNRPLSSLDSEKSHLMKTCNLLTQYFNGKADLKNLNLTISNNGEAKASATKDLLTNMEESSTKTTEQDQKLIDHVPKSAINKAS
SSKEIPNKEQKLAQLSIFYGGKVVVFDDFPAEKARAVMLLASKGISNNSCAIFQTTTTTQTNGANNFDLPIARRSSLYRFLEKRK
DRDTARAPYQMHNPLQSSSRTREDHFDLNF
>PGSC0003DMP400007762
MEESSTKTTEQDQKLIDHVPKSAINKASSSKEIPNKEQKLAQLSIFYGGKVVVFDDFPAEKARAVMLLASKGISNNSCAIFQTTT
TTQTNGANNFDLPIARRSSLYRFLEKRKDRDTARAPYQMHNPLQSSSRTREDHFDLNF
>PGSC0003DMP400050165
MGLTVKQEILEEPATDPAGSTSSAMHWSFSNKAHPQYHLSFKGQENNNNNNKPKIGFESLASAGLVTITTTTDIVDTIHRPYSTQI
GAHHVPTRNGVVGTTELRSAPRTSPGPAQLTMFYAGSVCVYDNISPEKAQAIMLLAGNAPISTTIRNFPSHDHNNNNTNETTITR
SIRVLKSPSNTTEVSKIESRQPPSHNLSAVPQARRASLARFLERRKERVVNASPYGNGKQSSQHMMNFTINSSGSSTPLPATN
>PGSC0003DMP400055225
MERDFMGLNIKDSLAVVKEEPVESSKDSGFRWPMSSKVGVPHFMSLNSAHDENTFKALSATDGVDAGVKRQSGELQMKQVLGGIP
VTAPHSMLPSRGSVAGTTEPWFNSKSCAAPAQLTIFYGGTVNVFEDISPEKAQAIMFLAGHGCAPPNVVQPRFQLQASASKPAAA
DGVCVNQTPNMLPASGLSSPMSVSSHPIGQSDGSSGNKDDMKMSKTANSSVTPLVKLDTSKIVTSLGPVGATTIMTAAVPQARKA
SLARFLEKRKERVMNLAPYCLSKKSPEYSTPESNGVGFSATSSPLLAGKET
>PGSC0003DMP400055226
MERDFMGLNIKDSLAVVKEEPVESSKDSGFRWPMSSKVGVPHFMSLNSAHDENTFKALSATDGVDAGVKRQSGELQNVHAIHLPY
DVKMLPFNMNNPSYKTHFGGSGQMKQVLGGIPVTAPHSMLPSRGSVAGTTEPWFNSKSCAAPAQLTIFYGGTVNVFEDISPEKAQ
AIMFLAGHGCAPPNVVQPRFQLQASASKPAAADGVCVNQTPNMLPASGLSSPMSVSSHPIGQSDGSSGNKDDMKMSKTANSSVTP
LVKLDTSKIVTSLGPVGATTIMTAAVPQARKASLARFLEKRKERVMNLAPYCLSKKSPEYSTPESNGVGFSATSSPLLAGKET
>PGSC0003DMP400027452
MDSRMEIDFMDLNSKPQLSEMEKQHKRASGMKWPFSLADLATHHENTFFQNYNSTPIVSINSKNSSLNNYKSTNDPQYFGGAFPL
VAKTSTYGSRKNYDNCSPNESTLTIFYMGEVHIFPGISPEKAELIIDLVSKSTTLHMDEILEKVMNKEKYEENKSDPSNASTNYA
KGALAMARRATLARFLEKRKHRLIKARPYLYGENLSKFPFDIQQEEEETASSSVHWEN
>PGSC0003DMP400002154
MRRKCNLELRLVPPAPYFDSMGNKESIDEEKEQQQLTIFYDGKVVVSDATEVQAKAIIHLASREMKENTKTLSSLSEPPSPLLQS
QTEVSMKKSLQRFLQKRKSRTQAISPYHQQQ
>PGSC0003DMP400015429
MRRNCNLELRLMPPSLSTFSPNNCNNTSYFSMEEDKESTELENKSQPLTIFYNGKFVVSHVTDLQAKAIIYLASRETEEKTNKTS
SPMSEPSSPLLQPQTVKKSLQRFLQKRKNRIETTSPYHH
>PGSC0003DMP400015428
MRRNCNLELTLMPPSISDNFSSKNCTMEDQQLENKQSQQLTIFYNGKFVVSDATELQAKAIIYLASRGMEVKTNKMSDPSSPLLQ
PQTGLFMKRSLQGFLQKRKRRVQATSPYHH
>PGSC0003DMP400024951
MERDFMGLTVKQEVLEEPIDPAPLRSSAMQWSFSNNVTAHPQYLSFKSAPEDKPKIGFDSLASTGLVTITTTEAVDSSHRTYSDV
TQKNMMLERQGGTHYTTTTFSPHHYDAHSVHRSHGVRVLPLASPTNQISVSMTMPGHKSFISPVGQNLITTVNQLPGAGALVASP
ISAIPSSSTVVGTTDLRGAPKTPPGPAQLTIFYGGSVCVYDNVSPEKAQAIMLLAGNAPPVAPNATSTLSPVQAPIPKSLAIDPF
VVNQCRNTTPTLASPISITSHGGTQSARVSRNTNGVTIIKSIGVLPSPSLKAEPSKVASSIGSFPASLVPSAVPQARKASLARFL
EKRKERVISASPYPPNSKQSPECSTPELGSRSLSMNSSGSCPPHIINLVK

>PGSC0003DMP400002158
MRRKSNLELTLSPSNFLMEDQQLENEQSQQLTIFYNGQFVVSHVTQLQAKAIIYRASREMEEKSNKMSEPSSPLLQPQTGRFMDK
SLQRFLQKRKNRIQTTSPYHH
