# Supplementary material for: JAZ Repressors: Potential Involvement in Nutrients Deficiency Response in Rice and Chickpea
Source: Front Plant Sci. 2015 Nov 10;6:975. doi: 10.3389/fpls.2015.00975 (PMC4639613; doi:10.3389/fpls.2015.00975)
Supplement: Supplementary file 2 [file Image1.PDF]

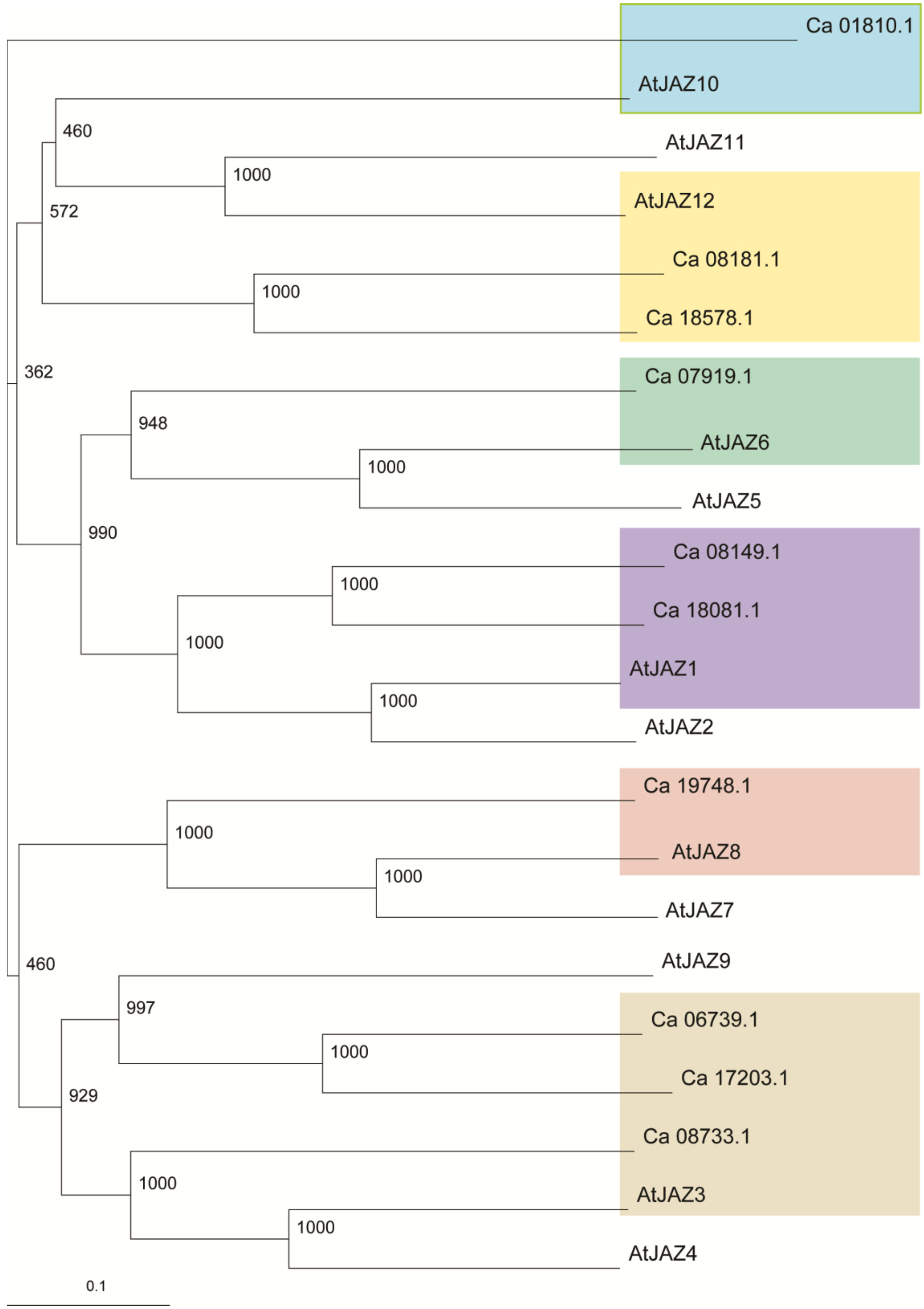


**Figure S1**. Phylogenetic tree of CaJAZ and AtJAZ proteins. Amino acid sequences of JAZ proteins were aligned in ClustalX and phylogenetic tree was constructed using NJ method. Scale bar represents amino acid substitution rate, bootstrap values are mentioned at each node. Colored blocks shows the homolog proteins.

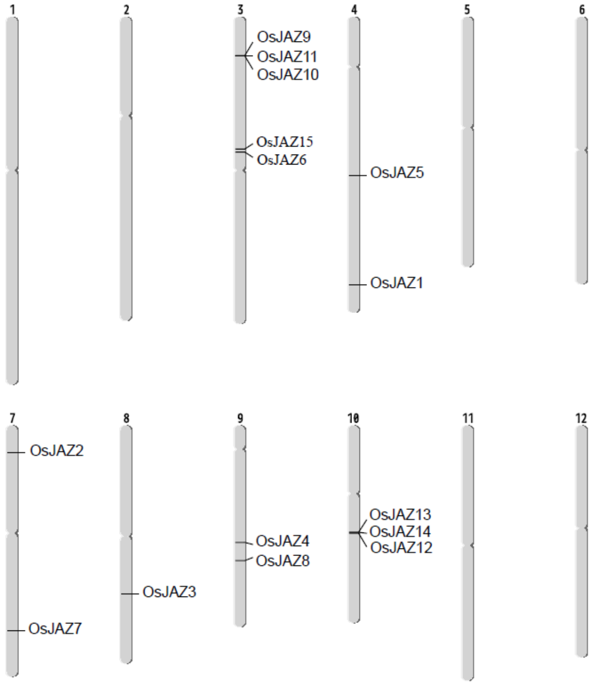


**Figure S2.** Chromosomal localization of *JAZ* genes in rice. Rice JAZ genomic sequence was used for analyzing chromosomal localizations in Oryzabase database.

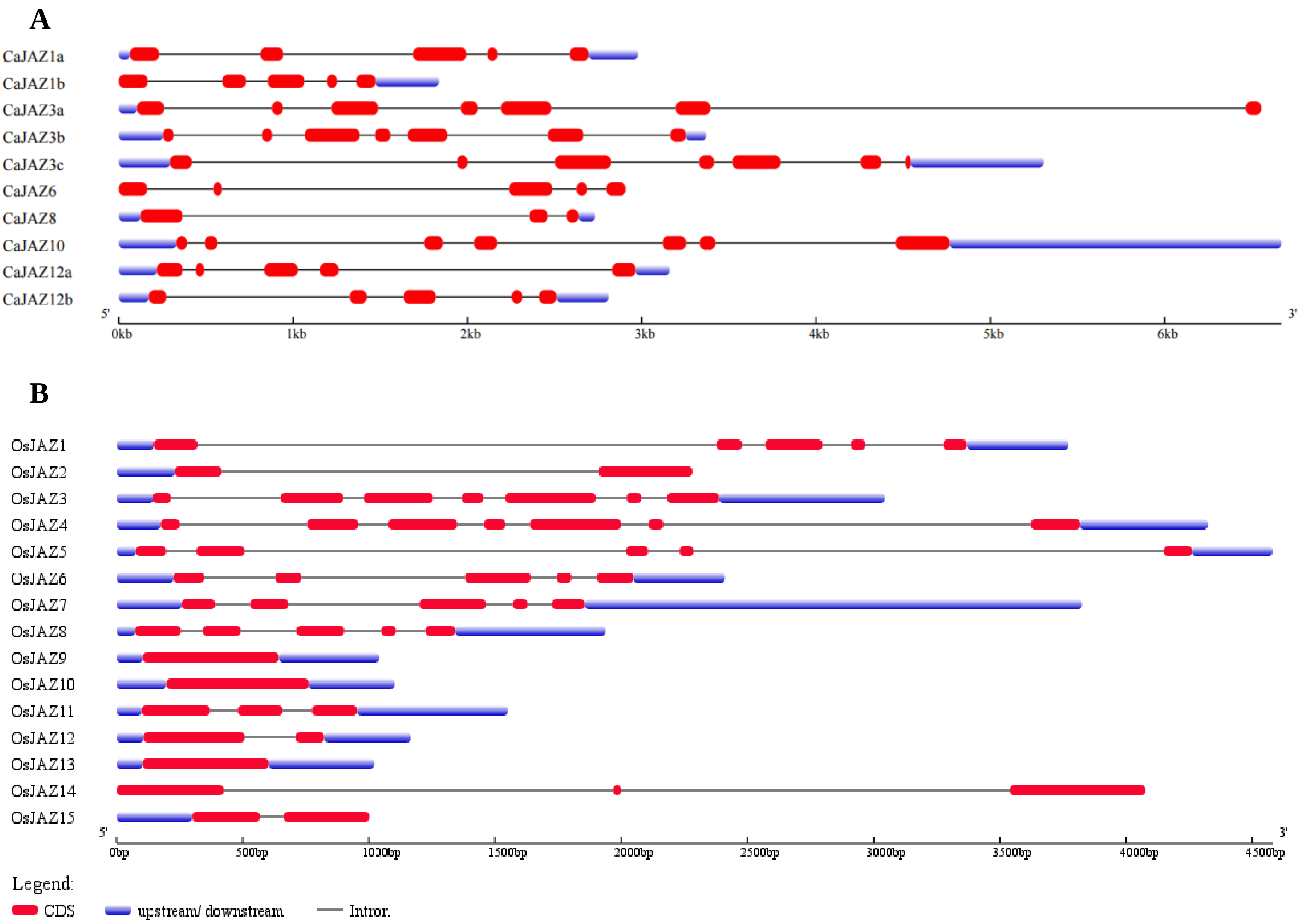


**Figure S3.** Schematic representation of **A)** Chickpea and **B)** Rice *JAZ* genes showing exons, introns and UTR's with red boxes, single line and blue boxes, respectively from 5' to 3' direction. Schematic were generated using GSDS (http://gsds.cbi.pku.edu.cn/) server.

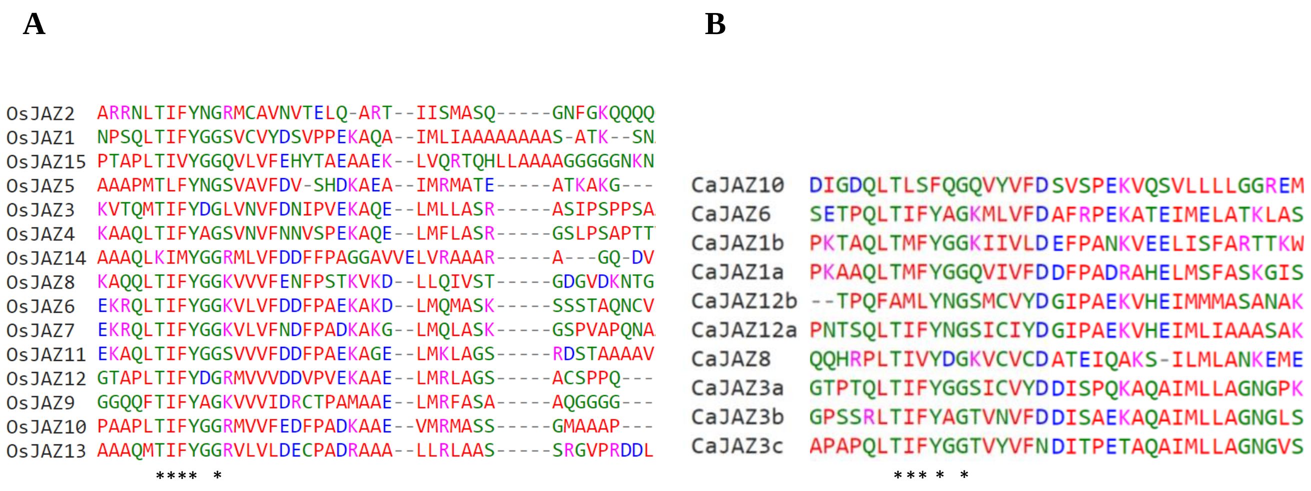


**Figure S4**. Protein sequence alignment of N terminal region of rice *JAZs* **A)** Rice and **B)** chickpea showing conserved TIFY domain. * indicate the conserved amino acids within TIFY motif.

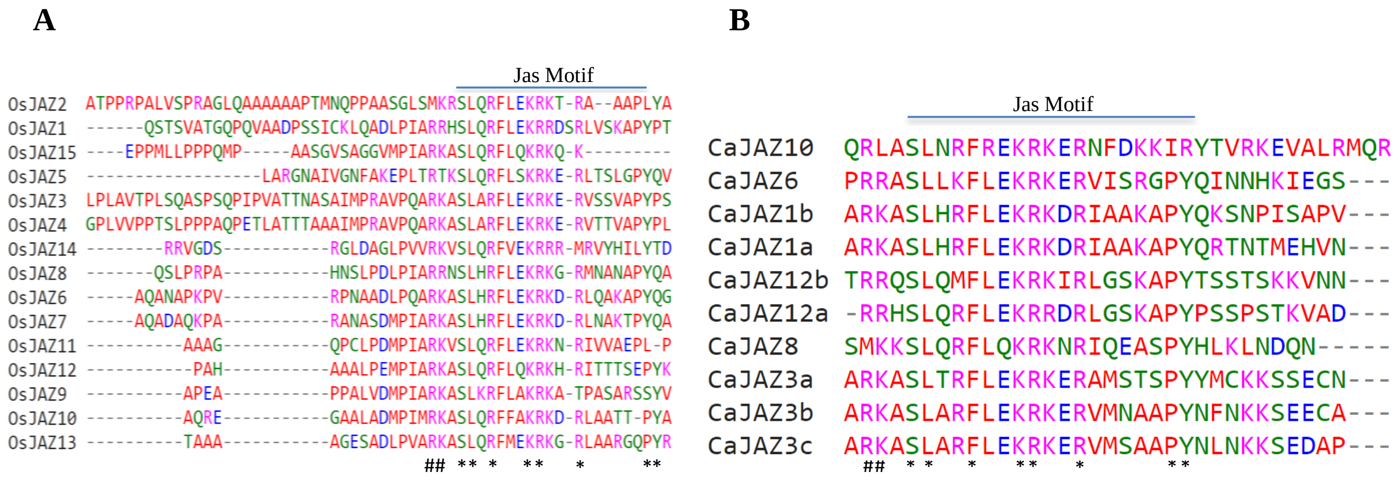


**Figure S5.** Multiple sequence alignment of A) rice and B) chickpea *JAZs* showing conserved JAZ degron and Jas motif. * indicates the conserved amino acids within Jas motif while # indicates the conserved basic amino acids within JAZ degron.

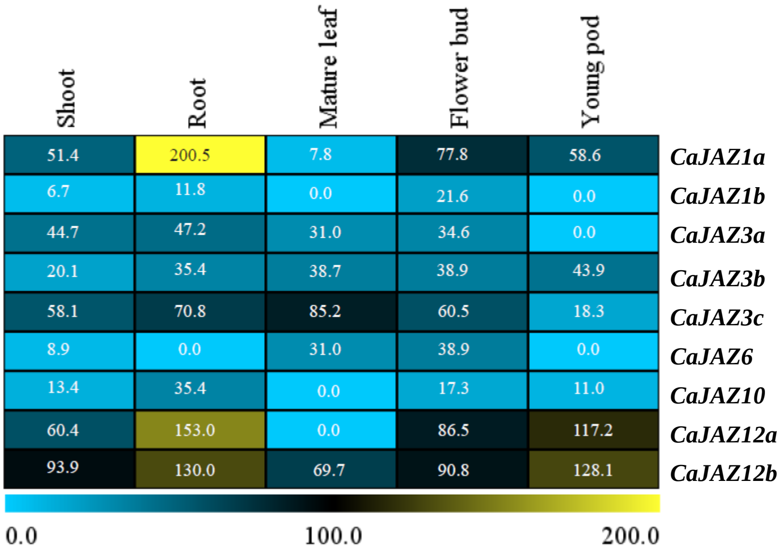


**Figure S6**. Tissue-specific (Shoot, Root, Mature leaf, Flower bud and Young pods) expression of CaJAZ genes, retrieved from CTDB. Expression level are represented in RPM (Reads per million) values, Color scale bar represents RPM values of lowest, middle and highest expressing *JAZ* genes.

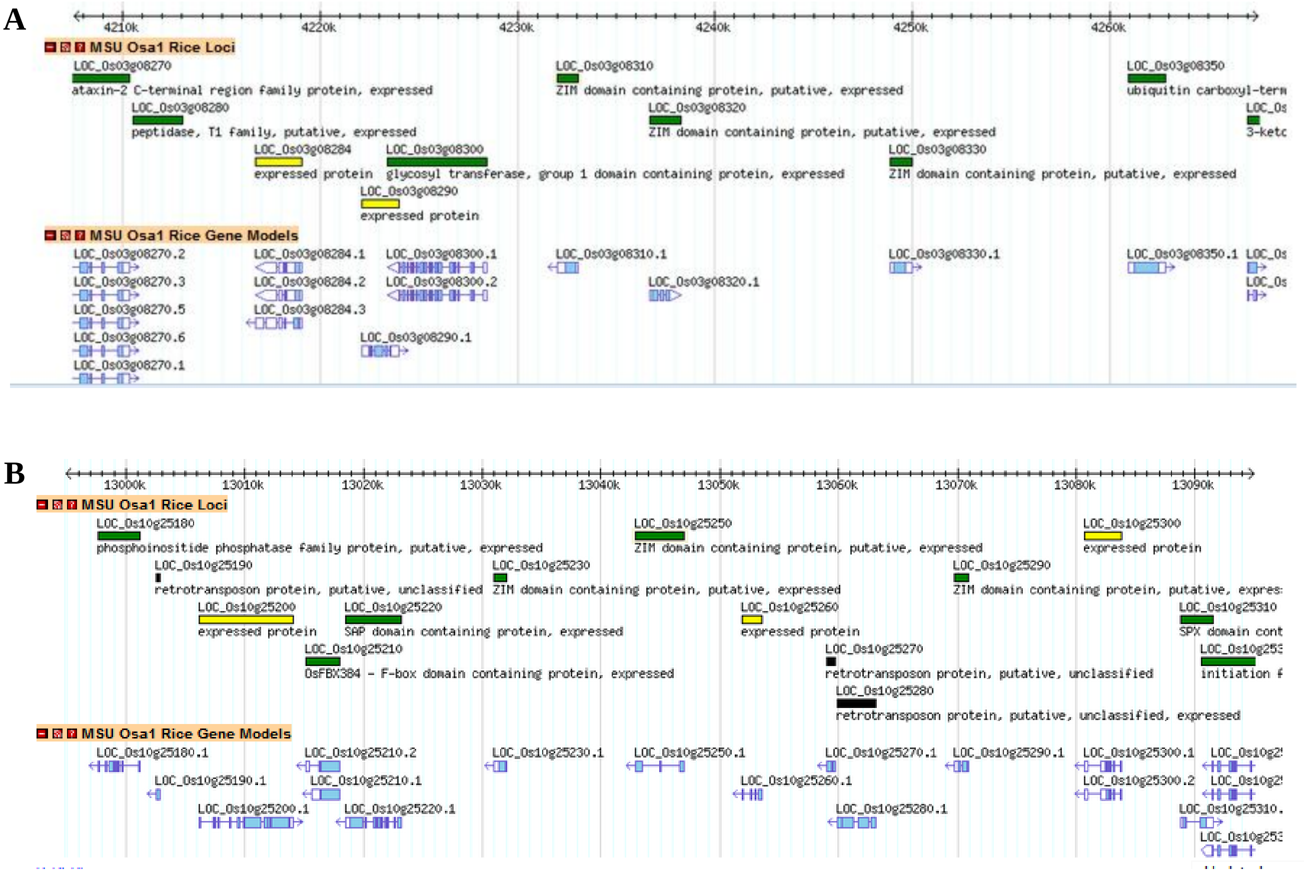


**Figure S7**. Genome browser snapshots of RGAP showing **A)**LOC_Os03g08310-30 and **B)** LOC_Os10g25230-90 rice JAZ genes clusters.

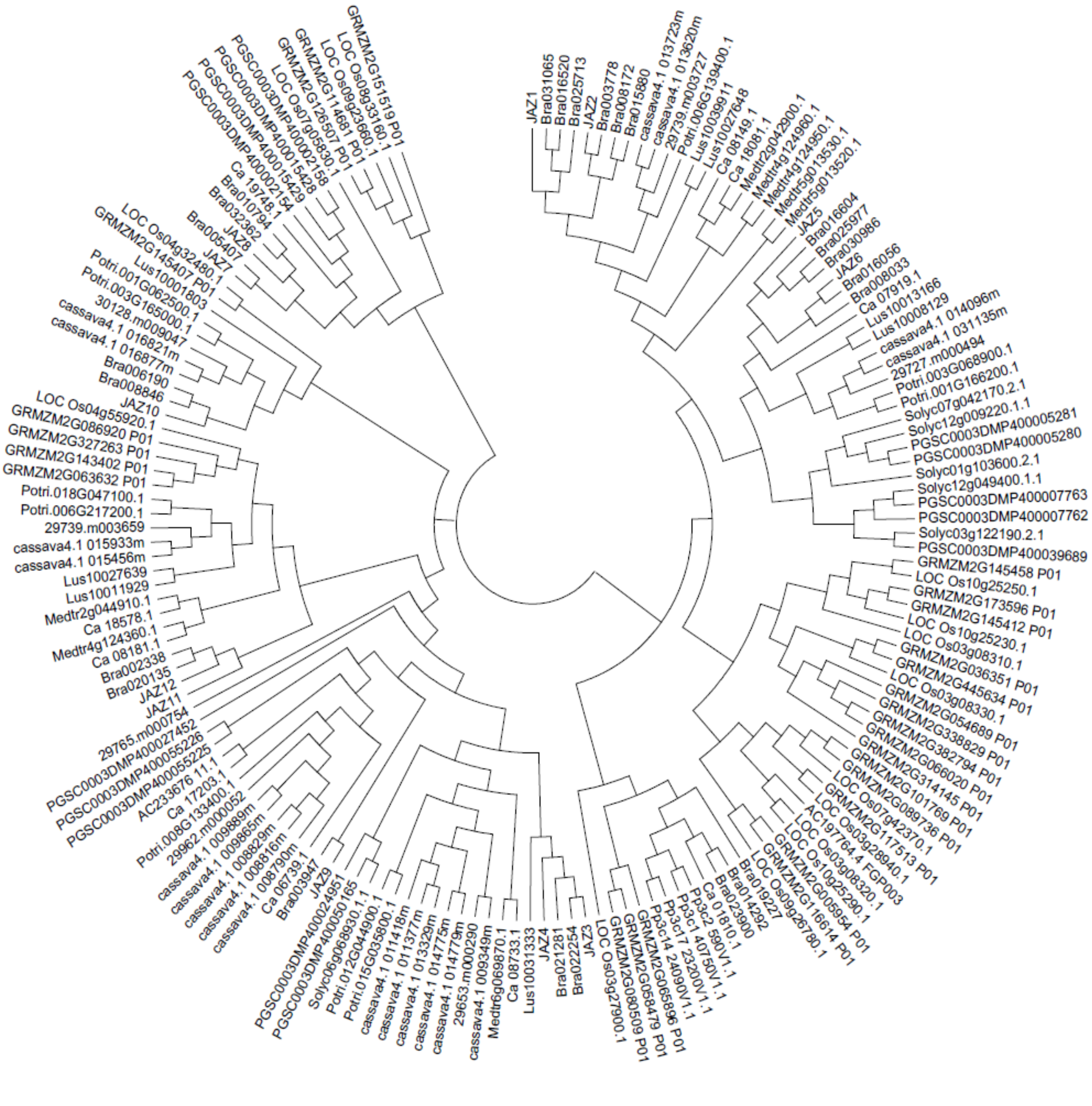

**Figure S8.** Phylogenetic Analysis of JAZ proteins (with intact TIFY and Jas motifs) found in different organisms. 165 JAZ proteins from different organisms were used for alignment and phylogenetic tree was visualised using MEGA 6.06 software.

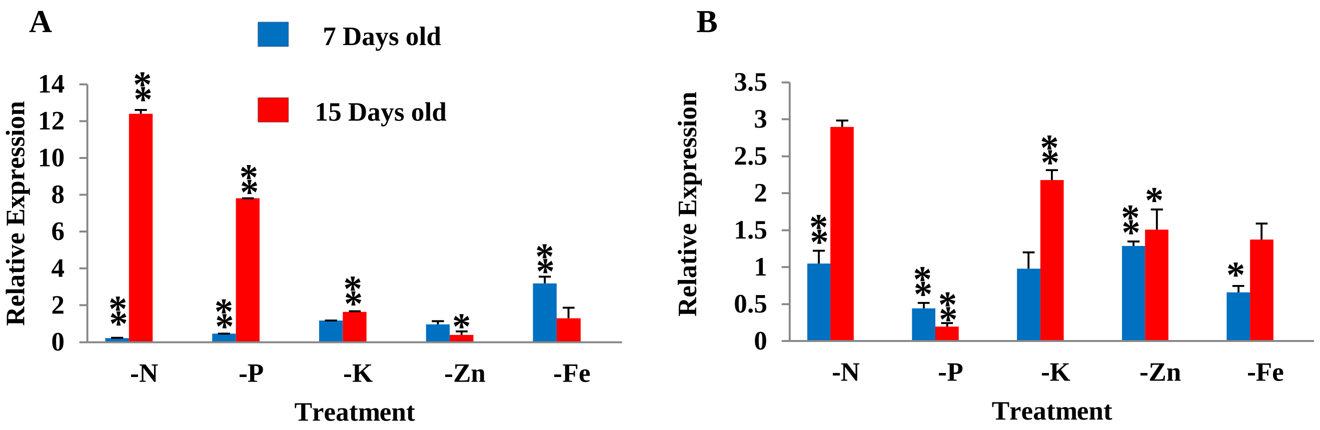


**Figure S9**. Relative expression profile of JA biosynthetic gene A) *OsAOS2* B) CaAOS1 under N, P, K Zn and Fe deficiency after 7 (early response) and 15 days (late response) of respective stress treatment in rice and chickpea roots respectively. qRT-PCR was used for quantitation of gene expression. Each bar shows the average of three biological replicates. Relative mRNA levels in treated plants were calculated using unstressed plants as control. *Ubiquitn 5* (for rice) and *EF1a* (for chickpea) gene was taken as endogenous control. (*p< 0.05, **p< 0.01).
